# Integrated metabolomics data analysis to generate mechanistic hypotheses with MetaProViz

**DOI:** 10.1038/s44320-026-00231-8

**Published:** 2026-08-17

**Authors:** Christina Schmidt, Jannik Franken, Denes Turei, Dimitrios Prymidis, Macabe Daley, Christian Frezza, Julio Saez-Rodriguez

**Affiliations:** 1 Heidelberg University, Faculty of Medicine, and Heidelberg University Hospital, Institute for Computational Biomedicine, Heidelberg, Germany; 2 University of Cologne, Faculty of Medicine and University Hospital Cologne, Institute for Metabolomics in Ageing, Cluster of Excellence Cellular Stress Responses in Aging-associated Diseases (CECAD), Cologne, Germany; 3 German Cancer Research Center (DKFZ), Division of Theoretical Systems Biology, Heidelberg, Germany; 4 University of Cologne, Faculty of Mathematics and Natural Sciences, Institute of Genetics, Cluster of Excellence Cellular Stress Responses in Aging-associated Diseases (CECAD), Cologne, Germany; 5 University of Cologne, Center for Molecular Medicine (CMMC), Cologne, Germany; 6 European Molecular Biology Laboratory, European Bioinformatics Institute (EMBL-EBI), Hinxton, UK

**Keywords:** Computational Biology, Metabolism

## Abstract

The lack of standardised workflows and ambiguous metabolite annotations hampers metabolomics integration with prior knowledge, thus limiting the extraction of meaningful biological insights. We present MetaProViz (**Meta**bolomics **Pr**ocessing, functi**o**nal analysis and **Vi**suali**z**ation), an open-source Bioconductor R package for metabolomics data analysis that integrates prior knowledge to generate mechanistic hypotheses (https://saezlab.github.io/MetaProViz/). MetaProViz operates on annotated intensity values and offers a flexible framework consisting of five modules: processing, differential analysis, prior knowledge integration, functional analysis and visualisation, applicable to intracellular and exometabolomics experiments. To improve functional analysis, we created the **Met**abolism **Sig**nature **D**ata**b**ase (MetSigDB), a collection of annotated metabolite sets. MetSigDB includes pathway-metabolite, metabolite-receptor, metabolite-transporter sets, and chemical class-metabolite sets. MetaProViz enables the conversion of gene sets to metabolite sets, metabolite identifier expansion and analyses mapping ambiguities. The MetaProViz functional analysis toolkit includes sample metadata analysis, enrichment analysis and biologically informed clustering. By applying MetaProViz to kidney cancer metabolomics data, we identified increased methionine usage in line with decreased methionine levels in tumour samples. In summary, MetaProViz facilitates and improves the analysis and interpretation of metabolomics data.

## Synopsis

MetaProViz is an open-source Bioconductor R package that integrates curated prior knowledge and metabolite annotation handling to enable reproducible metabolomics analysis, improve functional interpretation, and generation of mechanistic hypotheses from intracellular and extracellular metabolomics.

- Developed as a modular package combining processing, differential analysis, prior knowledge integration, functional analysis, and visualisation for intra and extracellular metabolomics data.
- Established a curated resource, MetSigDB, comprising pathway-, cancer-, chemical class-, receptor-, and transporter-based metabolite sets to enhance functional enrichment and biological interpretation.
- Implemented ambiguity-aware analysis improving integration with prior knowledge by systematically quantifying ambiguities in metabolite identifier mappings and enabling ID translation, ID traversion and completion of ID annotation.
- Enabled metabolomics consumption- and release analysis and link of extracellular changes to intracellular mechanisms, illustrated by connecting increased methionine usage in ccRCC cells to survival-associated enzymes/transporters consistent with DNA-hypermethylation signatures.
- Implemented metadata analysis to identify main metabolite drivers based on patient metadata, which revealed an age-specific metabolic signature of dipeptides in ccRCC tumour samples.

## Abstract (web summary)

MetaProViz is an open-source Bioconductor R package that integrates curated prior knowledge and metabolite annotation handling to enable reproducible metabolomics analysis, improve functional interpretation, and generation of mechanistic hypotheses from intracellular and extracellular metabolomics.

## Graphical abstract

**Figure:**
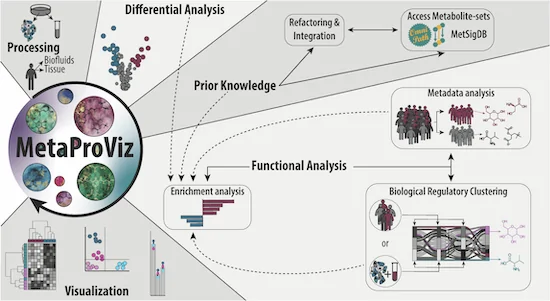

**Figure:**
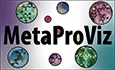

## Introduction

Advances in liquid chromatography–mass spectrometry (LC-MS) enabled the detection of thousands of metabolites from single biological samples, greatly expanding the depth of metabolomics datasets (Anh et al, 2024).

A broad range of tools has been developed to facilitate different steps in metabolomics data analysis (Spicer et al, 2017). The analysis of metabolomics data encompasses several steps, starting with pre-processing (including feature assembly and annotation, feature filtering, etc.), followed by normalisation of intensities and statistical analysis, culminating in functional and mechanistic analyses to ultimately interpret the data (Chen et al, 2022). There are tools that focus on specific steps of feature annotation (i.e. CAMERA (Kuhl et al, 2012), XCMS2 (Smith et al, 2006), and CliqueMS (Senan et al, 2019)) or the pre-processing of feature intensities, which encompasses feature selection, missing value imputation (MVI) (Wei et al, 2018) and data normalisation. The variety of MVI, normalisation and quality control techniques complicates the choice of methods that maximise data utility while minimising bias (Spicer et al, 2017). In experimental design, quality control samples are key for an effective removal of a large part of the technical variation (Mattoli et al, 2023). Yet, many tools lack implementations or reasonable defaults for these critical steps, ultimately requiring advanced expertise to navigate the analysis landscape. Furthermore, many tools implement workflows (Spicer et al, 2017), typically spanning from feature annotation to functional analysis. However, most workflows are only accessible as web apps (Spicer et al, 2017), with the notable exception of MetaboAnalystR 4.0 (Pang et al, 2024). Their user-friendly interfaces often hide key settings, hindering compliance with today’s reproducibility standards (Chetnik et al, 2022).

Functional interpretation is further hindered by the lack of unambiguous and reproducible metabolite annotations (Chaleckis et al, 2019). Since high-throughput MS methods are unable to distinguish fine structural details (e.g., stereoisomers or constitutional isomers) (Sumner et al, 2007; Salek et al, 2013; Calderón and Lämmerhofer, 2022), features do not correspond to fully defined structures. This is in discrepancy with assigning one particular, unambiguous IUPAC (International Union of Pure and Applied Chemistry) name or InChI (International Chemical Identifier) in addition to metabolite identifiers (IDs) as recommended by the Metabolomics Standards Initiative (MSI) (Sumner et al, 2007; Salek et al, 2013; Calderón and Lämmerhofer, 2022). Ultimately, this necessitates the assignment of multiple database entries to one feature, or one database entry representing all possible structures at once, since databases can have entries with different degrees of ambiguity (Pham et al, 2019) (i.e. CHEBI:16449 covers all varieties of alanine). This complexity in metabolite identifier assignment makes it difficult to link prior knowledge to features. Coupled with the challenge of defining the background set (Wieder et al, 2021), this undermines the robustness of enrichment analysis, like over-representation analysis (ORA). Moreover, the distinct nature of metabolites compared to genes often renders standard analytical approaches inappropriate, resulting in misleading or nonsensical results (Lee et al, 2025).

Here we present MetaProViz, **Meta**bolomics **Pr**ocessing, functi**o**nal analysis and **Vi**suali**z**ation, an R package to facilitate the analysis and biological interpretation of metabolomics data. MetaProViz operates on annotated intensity values and covers processing (i.e. total ion count (TIC) normalisation, feature filtering, etc.), quality control, differential metabolite analysis, prior knowledge integration, functional analysis and visualisation. MetaProViz includes a collection of annotated metabolite sets named **Met**abolism **Sig**nature **D**ata**b**ase (MetSigDB) with classical pathway-metabolite sets, chemical class-metabolite sets, cancer-metabolite sets, metabolite-receptor and metabolite-transporter sets. Furthermore, it enables the conversion of gene sets to metabolite sets. MetaProViz translates between metabolite IDs, defines mapping ambiguities between metabolite IDs of different databases and adds additional synonym IDs to identified features. MetaProViz also supports exometabolomics, the measurement of consumption and release of metabolites by cells (Silva and Northen, 2015; Stindt and McClean, 2022). Together with functional analysis, these tools help harness the potential of metabolomics data by enabling the generation of mechanistic hypotheses and improving biological interpretability. Through safeguards like automated checks and informative messages, MetaProViz addresses common pitfalls, improves decision-making and prevents mis- and overinterpretation, ultimately making metabolomics analysis more accessible.

## Results

### The MetaProViz software toolbox

**Figure 1:**
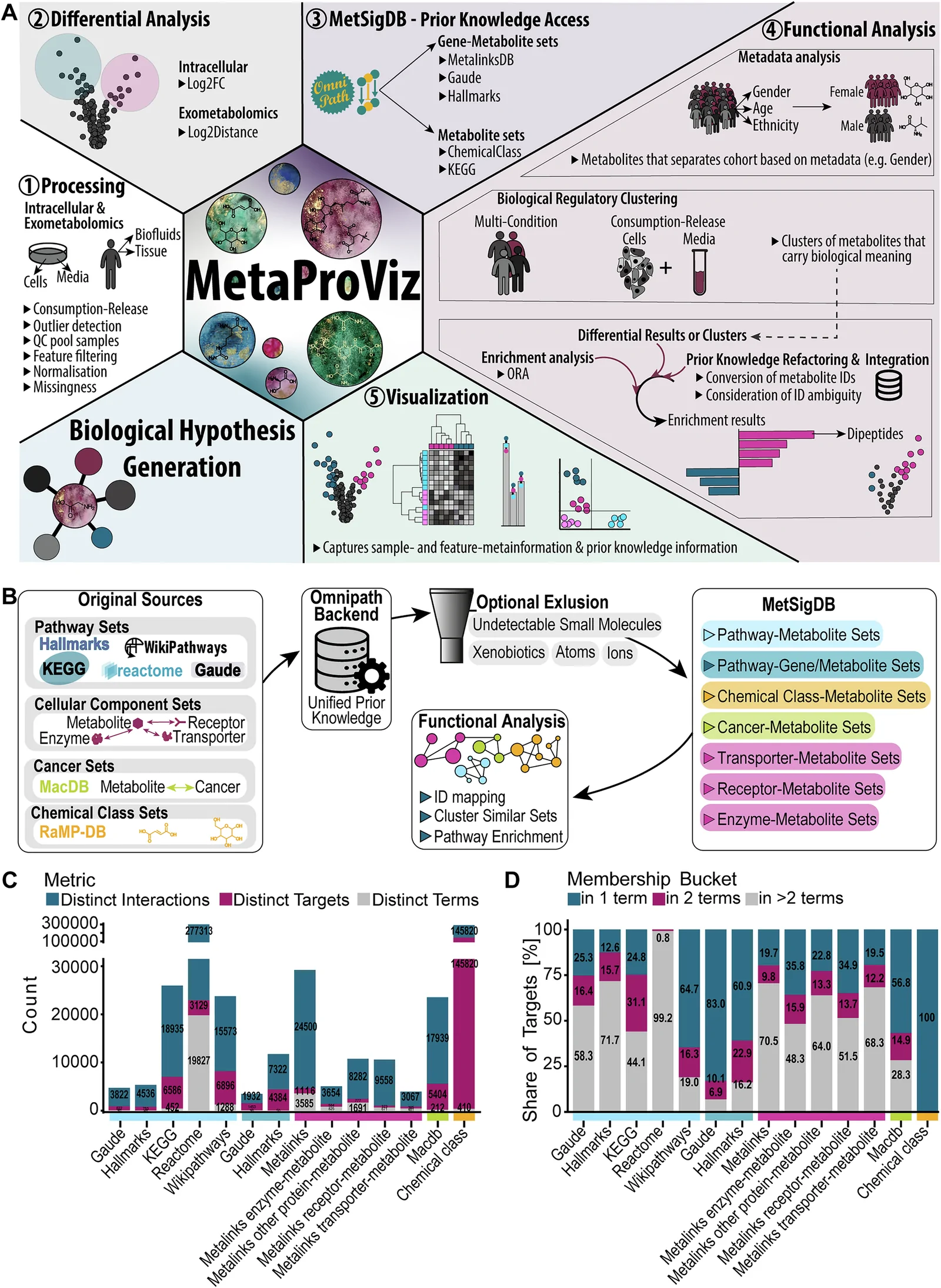
The contents and functionalities of MetaProViz. (**A**) Overview of the five MetaProViz modules (1–5) and their main functionalities to ultimately aid biological hypothesis generation. Results of modules 1–4 can be visualised using the visualisation module 5. Differential results from module 2 and prior knowledge from module 3 can be used together within module 4 to perform over-representation analysis. All modules can be used interactively as building blocks for a pipeline. PCA principal component analysis, QC quality control, FC fold change, ID identifier. (**B**) Overview of MetSigDB including original sources unified in Omnipath, groups of metabolites for exclusion and the final MetSigDB metabolite sets that can be used for functional enrichment. (**C**, **D**) Metabolite sets are displayed on the *x* axis including: pathway-metabolite sets (light blue), pathway-gene/metabolite sets (dark blue), metabolite-protein sets for transporter-, receptor- and enzyme-metabolite interactions (pink), cancer-metabolite sets (green) and chemical class-metabolite sets (yellow). Prior to plotting, metabolites from the exclusion list were removed. (**C**) Shows the number of distinct interactions, targets and terms for each metabolite set (Dataset EV1, sheet 3) and (**D**) shows the percentages of shared targets between sets within a metabolite set (e.g., shared metabolites between two pathways in KEGG) (Dataset EV1, sheet 5). Source data are available online for this figure.

MetaProViz consists of five main modules: (1) pre-processing, (2) differential metabolite analysis (DMA), (3) prior knowledge access, integration and optimisation, (4) functional analysis, and (5) visualisation (Fig. 1A). Each component can be used interactively or as building blocks within larger workflows. MetaProViz requires annotated and quantified features from peak integration as input, i.e., a matrix of intensities with annotated metabolites as column names and samples as row names. This format is commonly provided by facilities following feature assembly and annotation tailored to their extraction methods, instrumentation, analytes, and internal standards.

To guide the user, MetaProViz features different vignettes with example data, logs and return user messages, enabling even inexperienced users to make informed decisions. MetaProViz example datasets cover different experimental contexts, offering a robust foundation for testing and validating analysis workflows. The datasets have been sourced from kidney cancer studies (“Methods”, Appendix Fig. S1a): (1) intracellular metabolomics of human renal epithelial cells (HK2) and clear-cell renal cell carcinoma (ccRCC) cell lines 786-O, 786-M1A, and 786-M2A, (2) corresponding cell culture media profiling (exometabolomics), which additionally includes OS-RC-2, OS-LM1, and RFX-631 ccRCC cell lines (Sciacovelli et al, 2022), (3) patient tissue metabolomics from 138 matched ccRCC and adjacent normal tissue pairs with extensive patient metadata (Ricketts et al, 2018), and (4) transcriptomics and proteomics data from ccRCC tissue (Clark et al, 2019; Mora et al, 2024) to showcase the integration of metabolomics with other omics data. During the analysis, MetaProViz ensures reproducibility by capturing parameters, operations, warnings, and errors in a log file, while reporting key parameters, best practices, and implications of analytical choices. All tables and figures are saved in the desired format, organised into subdirectories reflecting the workflow, and with easy-to-identify file names (Appendix Fig. S1b). These outputs include the final tables, intermediate results, prior knowledge and quality controls. The results are also returned as plot objects and data frames, enabling their downstream use. To create figures, MetaProViz uses the ggplot2 package (Wickham, 2016) primarily with custom adjustments to labels, colours, and resolution for optimal slide and print quality. These publication-ready plots save users time by providing high-quality visualisations that meet professional standards.

MetaProViz has built-in access to prior knowledge adapted for enrichment analysis (Fig. 1B). This is done by systematically excluding non-specific metabolites (e.g. H_2_O or CO_2_), ions or xenobiotics in an optional exclusion step (Fig. 1B). We refer to the collection of these metabolite sets as MetSigDB, which currently includes (i) pathway-metabolite sets (KEGG (Kanehisa et al, 2017), Reactome (Milacic et al, 2024), WikiPathways (Agrawal et al, 2024), Gaude (Gaude and Frezza, 2016), Hallmarks (Liberzon et al, 2015)), (ii) pathway-gene/metabolite sets (Gaude (Gaude and Frezza, 2016), Hallmarks (Liberzon et al, 2015)), (iii) chemical class-metabolite sets that classify chemical compounds into a hierarchical system based on their structural features (Braisted et al, 2023), (iv) cancer-metabolite sets (MacDB (Sun et al, 2023)) and (v) MetalinksDB (Farr et al, 2024) metabolite-protein sets including receptor-, transporter- and enzyme-metabolite sets (Fig. 1B–D; Dataset EV1). In addition, MetaProViz offers conversion of any gene-set into gene-metabolite sets using metabolic reactions of a filtered version of Recon-3D accessed from the COSMOS prior knowledge network (Dugourd et al, 2021), which MetaProViz directly provides for the Hallmarks pathway-gene sets (Liberzon et al, 2015) and pathway-gene sets from Gaude et al (Gaude and Frezza, 2016) and converts those into pathway-gene-metabolite sets. The database knowledge in MetaProViz can also be used for other purposes, for example, the metabolite-receptor, metabolite-transporter, and enzyme-metabolite relationships for network analysis, and the ID translation utility to translate any data frame column with metabolite IDs.

### Improving the connection between prior knowledge and metabolomics features

**Figure EV1:**
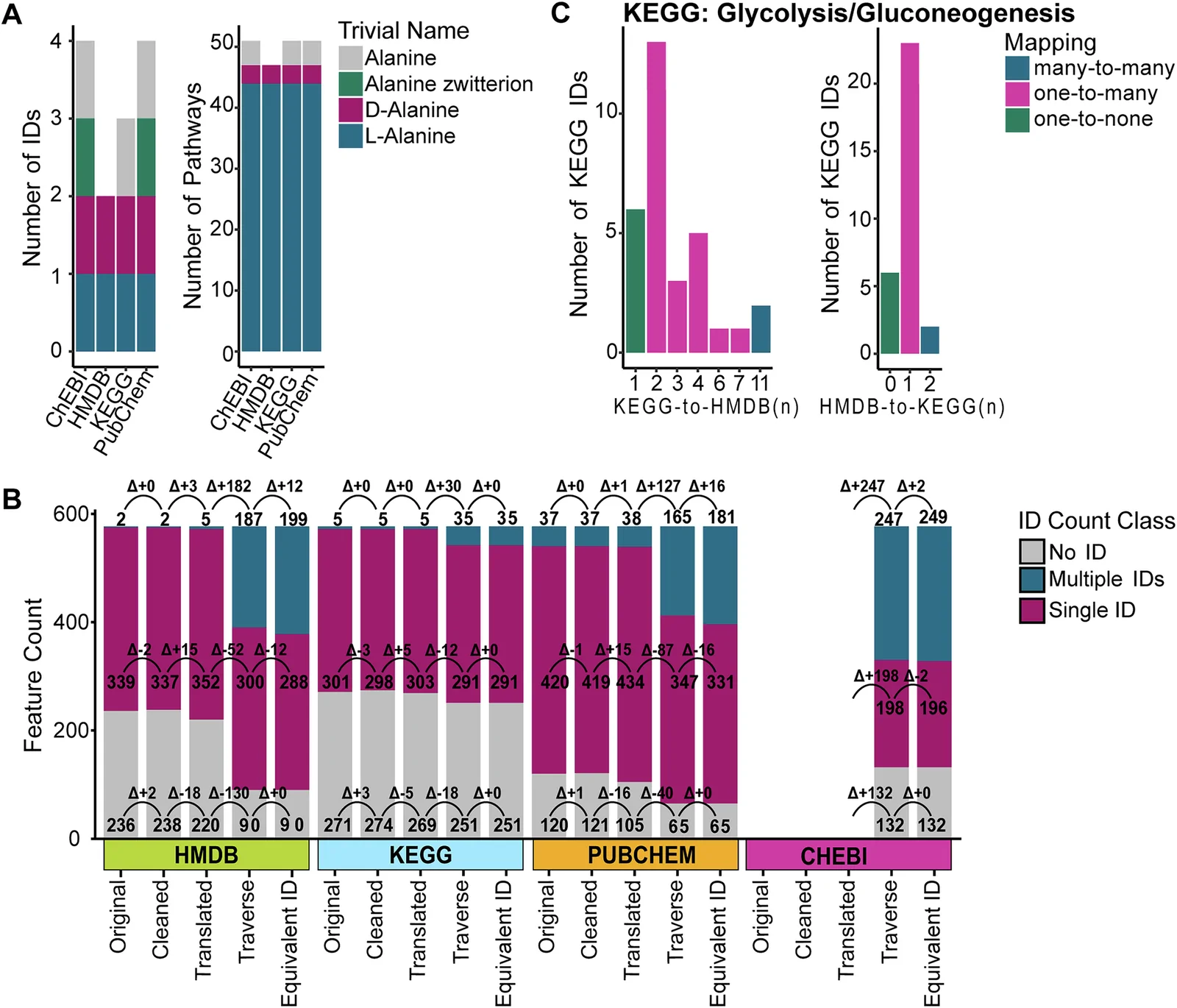
ID mapping problems in metabolite prior knowledge. (**A**) Example of the presence of multiple metabolite IDs for the amino acid alanine in ChEBI, HMDB, KEGG and PubChem databases (left) and the number of pathways these metabolite IDs can be mapped to by RaMP-DB (right). (**B**) Stacked bar graphs showing the frequency at which a certain number of metabolite IDs per integrated peak are available (no, one or multiple) with the number of feature counts (*y* axis) for HMDB, KEGG, PUBCHEM and CHEBI within the ccRCC patient feature space. “Original” is referring to the IDs provided by the original publication, “Cleaned” is after our ID mismatch check (= deltas are mostly negative), “Translated” are IDs that were added by translating from CAS numbers, “Traverse” are IDs added after using MetaProViz ID traversion method and “Equivalent ID” are IDs added based on stereoisomerism. The deltas describe the IDs gained through each step and show that over the course of the ID workflow, features without IDs (grey) are decreasing, whilst features with multiple IDs (blue) are increasing. (**C**) Ambiguity in the translation of metabolites in the “KEGG Glycolysis/Gluconeogenesis” pathway from KEGG to HMDB IDs (left) and then back (right) using MetaProViz; number of source IDs (bar heights) that map to a certain number of target IDs (*x* axis). Source data are available online for this figure.

Many databases that collect metabolite information, such as the Human Metabolome Database (HMDB)(Wishart et al, 2022), include multiple entries for the same metabolite with different degrees of ambiguity (Pham et al, 2019). This poses a difficulty when assigning metabolite IDs to measured data, where, e.g., stereoisomers are not distinguished. Hence, if detection is unspecific, it is crucial to assign all possible metabolite IDs to increase the overlap with the prior knowledge. For example, when assigning an HMDB ID to alanine, assigning both IDs for D-alanine and L-alanine has to be done as no unspecific HMDB ID is available (Fig. EV1A; Dataset EV2, sheet 24). On the contrary, when using ChEBI IDs, only one ID can be assigned, namely alanine (“CHEBI:16449”). However, this may lead to other problems as the ChEBI ID is not specific and does not map to most metabolic pathways in standard prior knowledge pathway-metabolite sets such as KEGG (Kanehisa et al, 2017), WikiPathways (Agrawal et al, 2024) and Reactome (Milacic et al, 2024) (Fig. EV1A; Dataset EV2, sheet 24). Substrate chirality is critical to enzymatic processes and the stereoselectivity of enzymes is homochiral with predominance of one particular enantiomer (e.g., D-sugars, L-amino acids, etc.), hence it is important that a specific metabolite ID maps to certain pathways.

**Figure 2:**
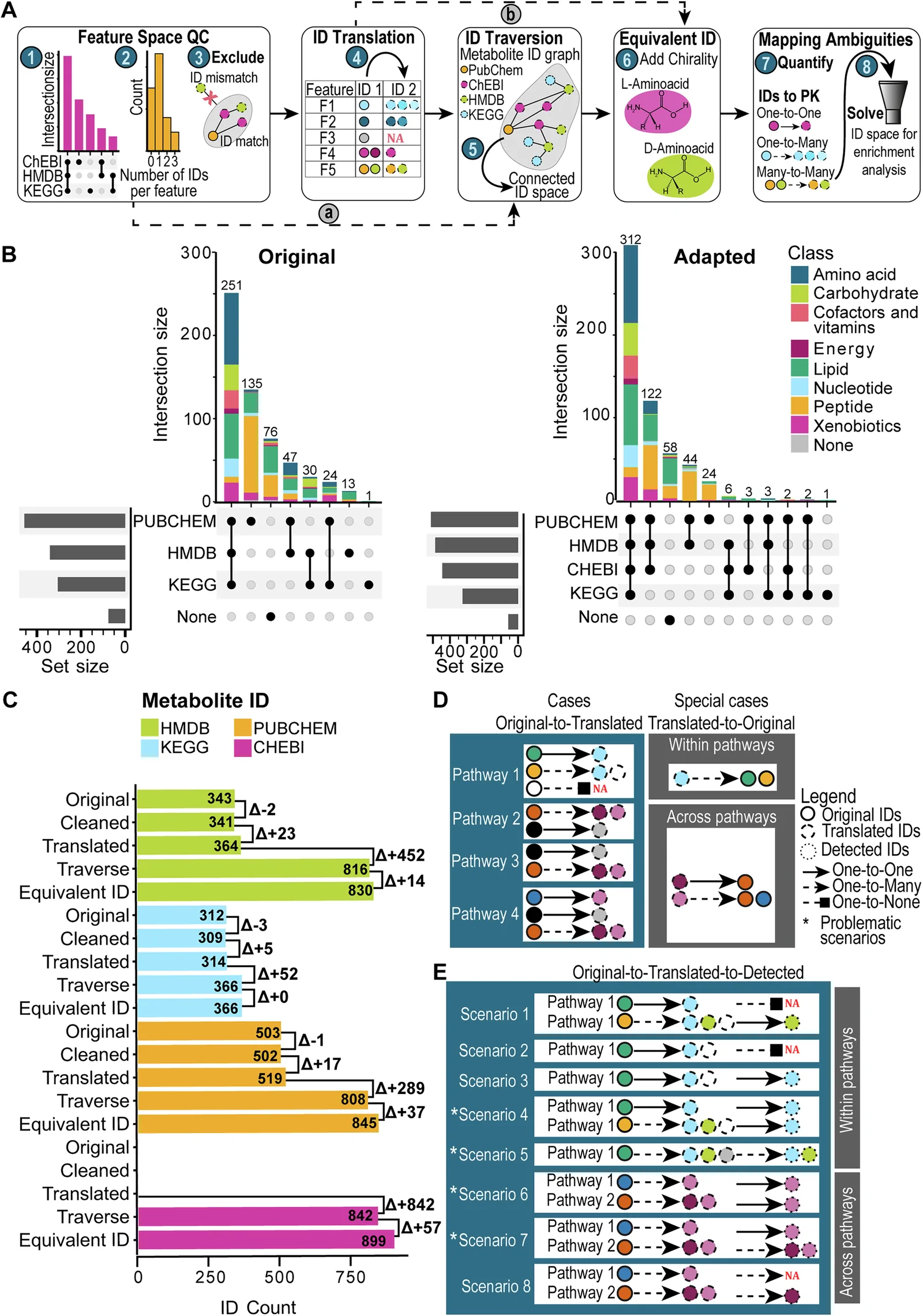
ID mapping problems in metabolite prior knowledge. (**A**) Overview of the feature ID workflow starting from annotated features with metabolite ID. The workflow is divided into 8 steps: features space quality control (QC) based on (1) metabolite ID intersection analysis (2) quantification of number of IDs per feature and (3) ID mismatch identification if more than one ID type per feature is available, which are solved manually; (4) ID translation from one ID type into another ID type; (5) ID traversion using an ID graph, which adds new ID types and adds IDs to all present ID types; (6) addition of equivalent ID types based on chirality; (7) quantification and (8) manual solving of mapping ambiguities. Often, either ID translation or ID traversion is performed, and hence one of the two is skipped (route **a**, **b**). (**B**) Upset plots of the ccRCC patients feature IDs as provided in the original publication (left) and after assigning additional potential IDs and translating between the provided ID types (right). (**C**) Number of metabolite ID (*x* axis) for HMDB, KEGG, PUBCHEM and CHEBI within the ccRCC patient feature space. “Original” is referring to the IDs provided by the original publication, “Cleaned” is after our ID mismatch check ( = deltas are mostly negative), “Translated” are IDs that were added by translating from CAS numbers, “Traverse” are IDs added after using MetaProViz ID traversion method and “Equivalent ID” are IDs added based on stereoisomerism. The deltas describe the IDs gained through each step. (**D**, **E**) Schematics of possible mapping cases between metabolite IDs ( = each circle corresponds to one ID) of a pathway-metabolite set (e.g., KEGG) to metabolite IDs of a different database (e.g., HMDB), with (**D**) showing many-to-many mappings that can occur within and across pathway-metabolite sets and (**E**) additionally showing the mapping to metabolite IDs that were assigned to the detected peaks within and across pathway-metabolite sets. Different scenarios within pathways (scenarios 1–5) and across pathways (scenarios 6–8) are displayed, highlighting problematic scenarios (4–7) that require further actions. Problematic scenarios should be addressed on a case-by-case basis as they also require biological information to be taken into account. Source data are available online for this figure.

Since existing solutions such as MetaboAnalystR 4.0 (Pang et al, 2024) do not handle ambiguous metabolite ID mapping (Dataset EV1, sheet 6 for detailed comparison), MetaProViz offers methodologies to solve such conflicts to allow robust mapping of experimental data with prior knowledge. In detail, MetaProViz performs feature space quality control, offers functionalities to increase the metabolite ID feature space, including ID translation, ID traversion through a metabolite ID graph and enantiomer addition and quantifies mapping ambiguities (Fig. 2A) (Braisted et al, 2023). Here we apply this to the ccRCC patients feature space, for which three different ID types have been provided by the authors (Hakimi et al, 2016), namely HMDB, KEGG and PubChem. Using MetaProViz, we first performed feature space quality control by analysing the existing metabolite ID space and found that only 43% of all features have HMDB, KEGG and Pubchem IDs assigned, whilst 23% only had a PubChem ID assigned (Fig. 2B, left and Dataset EV2, sheet 1). One explanation could be that the study was published in 2016 (Hakimi et al, 2016) when the databases covered fewer metabolites. In addition, the majority of features only had one database ID assigned per feature (Fig. EV1B); however, given machine detection limits, at least all possible stereoisomers should be assigned to the feature, unless there is a biological reason. Next, we checked for ID mismatches and found features with metabolite IDs that pointed to different structures, resulting in the removal of 2 HMDB IDs, 3 KEGG IDs and 1 PubChem ID (Figs. 2C and EV1B; Dataset EV2, sheets 2–3). In detail, we removed 41 partially incompatible cases, which means some IDs are connected in the network, whilst others are not. We also had 33 cases with no match between IDs (= fully incompatible), which we manually reviewed (Dataset EV2, sheet 2). Since the ccRCC patients feature space provided CAS numbers for some features, we performed ID translation using RaMP-DB prior knowledge (Braisted et al, 2023) for features that had no metabolite ID assigned, which reduced the number of features without any ID from 76 to 58 (Figs. 2B,C and EV1B; Dataset EV2, sheet 4). To further extend the overlap across ID types, we expanded the metabolite IDs across HMDB, KEGG and PubChem by building and traversing a metabolite ID graph. This fills the gaps by iteratively going through the cross-database mappings and collecting all connected IDs, whilst additionally adding ChEBI IDs to the features (Figs. 2B,C and EV1B; Dataset EV2, sheet 5). In addition, this will result in multiple metabolite IDs per feature since each database has entries with different degrees of structural resolution (Fig. EV1B). Lastly, we added equivalent IDs based on chirality (Fig. EV1B, “Methods”). Together, this increased the number of features with all initial ID types from 43 to 54% (Figs. 2B, right,  2C and EV1b; Dataset EV2, sheet 6). Increasing the metabolite ID space is an important step to maximise the overlap before mapping to prior knowledge, such as metabolite sets.

An additional consideration when integrating measured metabolite IDs with prior knowledge is the inconsistency in identifier types across resources; for example, KEGG pathways use KEGG IDs, whereas Reactome relies on ChEBI identifiers. Hence, assigning additional metabolite IDs by using MetaProViz’s workflow (Fig. 2A) is important (Fig. EV1A; Dataset EV2, sheet 24). During metabolite ID expansion via ID translation or traversion we can observe one-to-none, one-to-one, one-to-many and many-to-many mappings for the features and ID types, which for metabolite sets can happen either within a group, i.e., pathway, and/or across groups (Figs. 2D and EV1C; Dataset EV2, sheet 25). Using MetaProViz’s functionality to summarise mapping ambiguities during ID translation, we demonstrate that converting KEGG IDs from KEGG pathway-metabolite sets to HMDB IDs results in zero one-to-one mappings. Instead, most mappings are one-to-many, and some even involve many-to-many relationships (Fig. EV1C), as observed in the ccRCC feature space. This mapping ambiguity can lead to problems when set enrichment analysis is performed, since it can inflate or deflate the pathway-metabolite sets. Here we can have cases where both the prior knowledge metabolite sets and the measured data, or one of these, have multiple metabolite IDs per feature by applying ID translation/traversion. For all those cases, MetaProViz maps the detected metabolites to the pathway-metabolite sets and flags ambiguous or problematic scenarios to enable manual review. In detail, addition of metabolite IDs to a metabolite set by translation or traversion can lead to special cases such as many-to-one mappings (Fig. 2D, Pathway 1), where, for example, the original resource used the ID for L-Alanine (Pathway 1, green) and D-Alanine (Pathway 1, yellow) in the amino acid pathway, whilst the ID space we translate to only has an entry for Alanine zwitterion (Pathway 1, blue). In addition, many-to-one mappings can also occur across pathways (Fig. 2D, Pathways 2–4), where this mapping is only detected when mappings are analysed taking all pathways into account. Both of these cases deflate the pathways, which can also happen for one-to-none mappings (Fig. 2D, Pathway 1, white). There are also cases that inflate the pathway, such as one-to-many mappings (Fig. 2D, e.g., Pathway 2–4, orange mapping to pink and violet). It is important to ensure that the number of features assigned to each pathway matches the original composition defined in the prior knowledge resource (Fig. 2E). In detail, there are scenarios that will require a reduction in features per pathway (Fig. 2E, Scenario 4) or the merge of two detected features by sum or mean (Fig. 2E, Scenario 5). In addition, there are scenarios that only become apparent across pathways (Fig. 2E, Scenarios 6–8) where decisions on a case-by-case basis are required. Indeed, mapping the ccRCC feature space to MetSigDB KEGG pathways, we found 16 cases that required manual review, hence we removed IDs from our detected features for 14 of those cases (Dataset EV2, sheet 7, flag column 6).

In summary, the prior knowledge normalisation methods of MetaProViz increase the overlap between prior knowledge and measured data, leading to more accurate set enrichment results and improved functional interpretation. These functionalities include translating metabolite IDs, summarising mapping ambiguities that occur during ID translation and flagging problematic scenarios of mapping ambiguities (Fig. 1). Together with MetaProViz curated selection of annotated metabolite sets (Fig. 1B) and the implementation of ORA on differential analysis results or clusters of metabolites, these functionalities support the generation of mechanistic hypotheses.

### Preprocessing and quality control using MetaProViz

**Figure 3:**
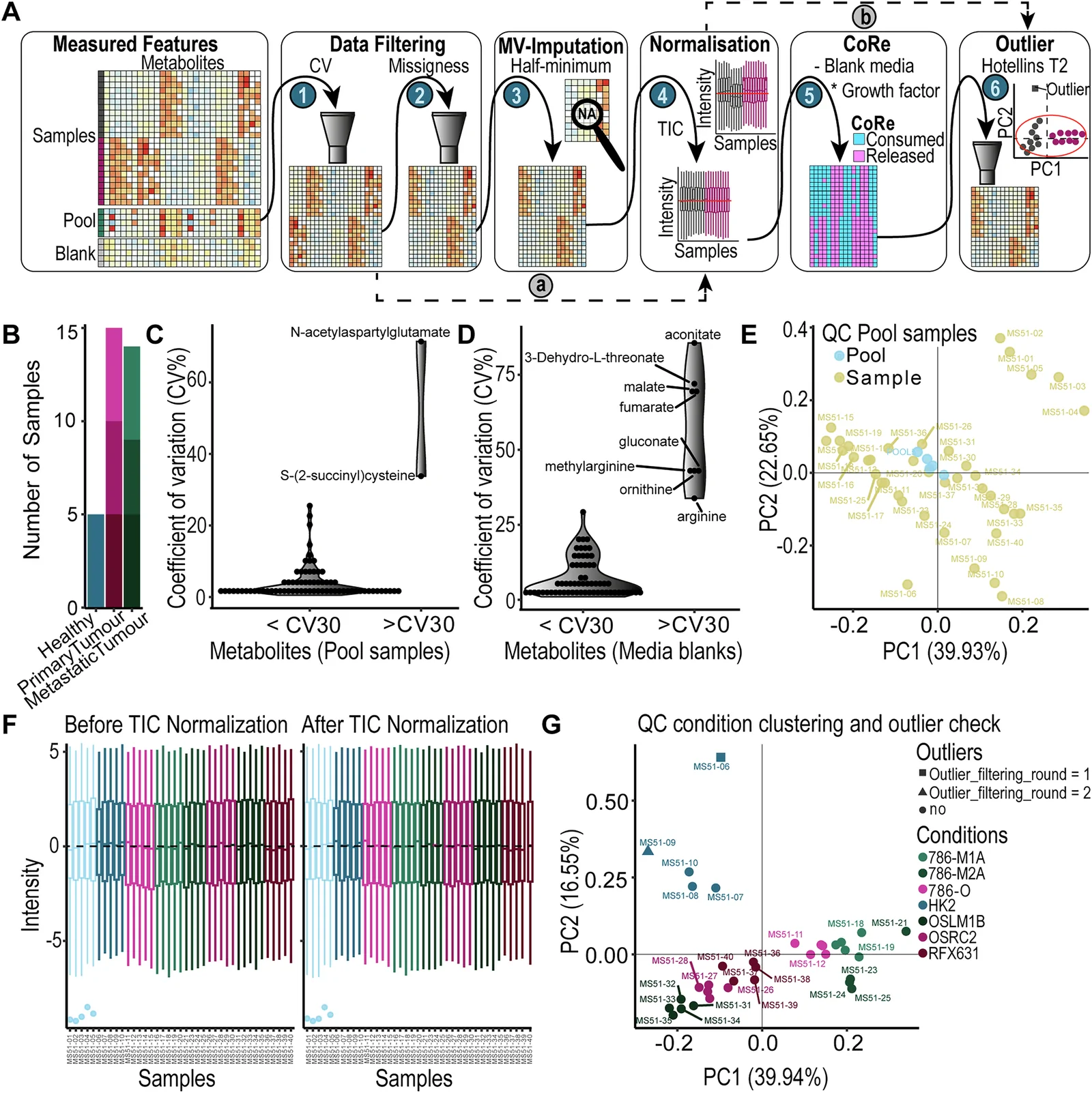
Quality control and processing of exometabolomics data using MetaProViz. (**A**) Overview of the pre-processing workflow starting from a matrix of annotated features and divided into 6 steps: Features are filtered based on (1) coefficient of variation (CV) of pool samples and blanks and (2) missingness in the biological samples, (3) if applicable MVI using half-minimum is performed, (4) TIC normalisation, (5) if applicable Consumption-Release (CoRe) normalisation by subtraction of blank media samples and by multiplying with growth factor and (6) outlier removal based on Hotellin’s T2 distribution. It is common that MVI and CoRe are skipped (route **a**, **b**). (**B**) Number of exometabolomics samples reflecting biological replicates from ccRCC cell lines, including healthy (blue: HK2), primary tumour (light to dark pink: 786-O, OSRC2, RFX631) and metastatic (light to dark green: 786-M1A, 786-M2A, OSLM1B). (**C**–**E**) Exometabolomics feature quality assessment of: (**C**) CV of each metabolite in pool samples (*n* = 3), (**D**) in media blank samples (*n* = 5), which are samples where no cells were cultured in, and (**E**) PCA showing metabolite pool samples clustering around the origin. (**F**) Boxplots of feature intensities before and after TIC normalisation (media blank = light blue; HK2 = blue; 786-O, OSRC2, RFX631 = light to dark pink; 786-M1A, 786-M2A, OSLM1B= light to dark green) (*n* = 5 biological replicates per condition). The centre line indicates the median, box boundaries represent the 25th and 75th percentiles, and whiskers extend to the most extreme observations within 1.5 times the interquartile range. Values beyond the whiskers are shown as outliers. (**G**) PCA plot of Hotelling’s T2 outlier detection performed on the processed and normalised exometabolomics data using 99% confidence interval (“Outlier_filtering_round = 1”, MS51-06, was removed). Detailed explanation, code and plots can be found in the vignette (https://saezlab.github.io/MetaProViz/articles/CoRe%20Metabolomics.html). Source data are available online for this figure.

MetaProViz offers a suite of pre-processing and quality control (QC) functions, with a focus on user-guidance and reproducibility. This suite includes functions for both the analysis of QC samples, such as pool samples, as well as functions for normalisation, MVI and outlier detection. These steps collectively help remove low-quality features and sample outliers, resulting in high-quality data suitable for addressing biological questions. Here, we apply MetaProViz functionalities to perform the pre-processing of exometabolomics data (Fig. 3A) from one renal control cell line and six ccRCC cell lines (Fig. 3B).

**Figure EV2:**
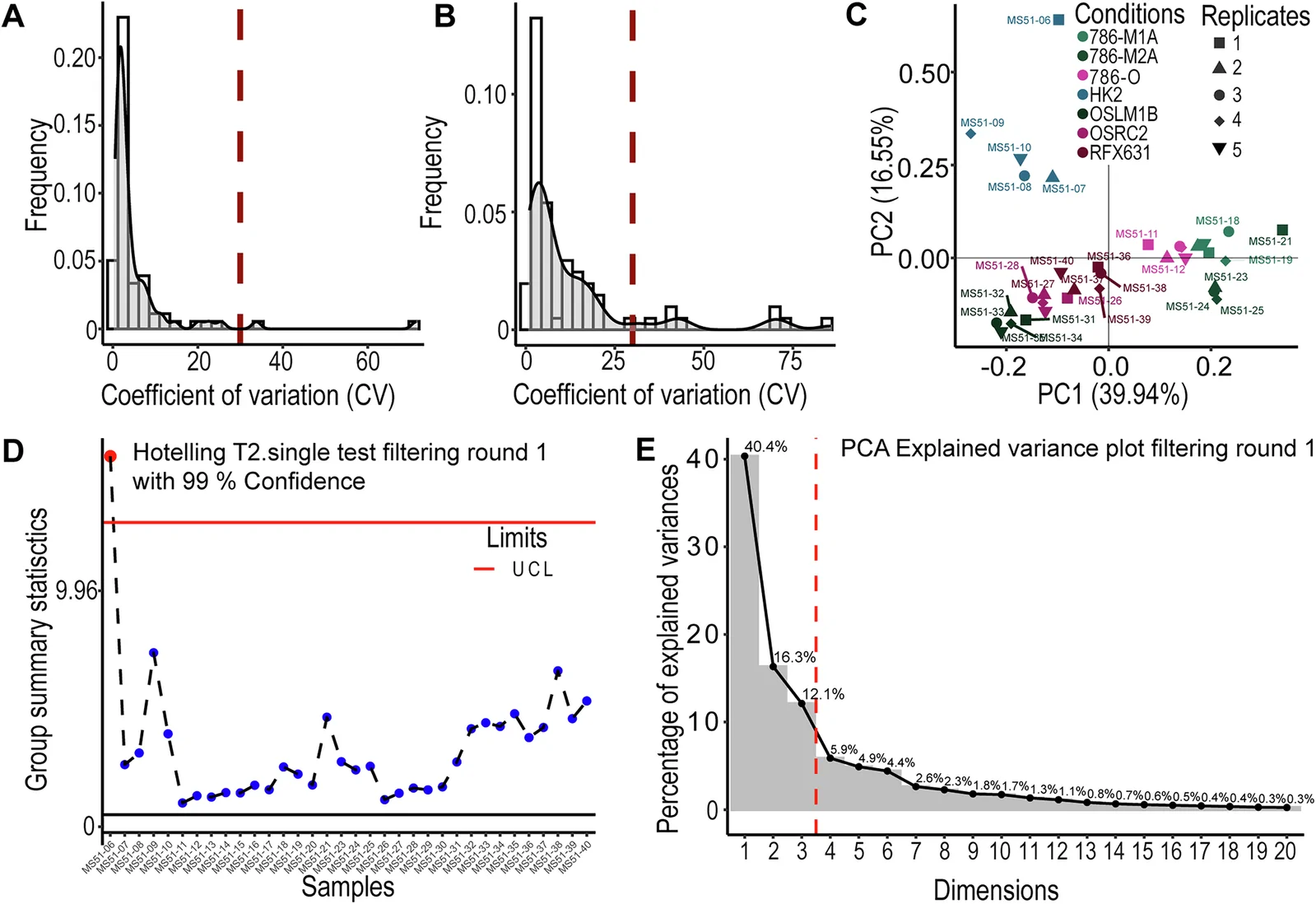
Quality control and processing of exometabolomics data using MetaProViz. Feature QC and outlier testing of exometabolomics data using MetaProViz. (**A**, **B**) Exometabolomics feature quality of (**A**) metabolite pool sample coefficient of variation (CV) (*n* = 3), (**B**) media blank samples (*n* = 5), which are samples where no cells were cultured in. (**C**) PCA plot with shapes for biological replicates to understand replicate spread. (**D**, **E**) Visualisation of different parameters of Hotelling’s T2 test results with 99% confidence interval of filtering round 1 including (**D**) Group summary statistics highlighting MS51-06 as outlier, which was removed and (**E**) explained variance of PCA plot with elbow (red line). Detailed code, explanation and plots can be found in the vignette (https://saezlab.github.io/MetaProViz/articles/CoRe%20Metabolomics.html). Source data are available online for this figure.

Solutions to identify low-quality features based on QC samples are multifaceted. Quality control (QC) samples, such as blanks (e.g., water or PBS), media blanks (media without cells), and pool samples (a homogeneous mixture of all samples), can be used to assess feature variability. Specifically, the coefficient of variation (CV) helps identify features with high variability and thus low detection accuracy. For blanks, minimal or no signal is expected, while media blanks and pool samples typically show CVs below 30% (Figs. 3C,D and EV2A,B; Dataset EV3). In our data, most measured features have CVs near zero, indicating high measurement confidence (left violin Fig. 3C,D). In addition, pool samples are expected to cluster near the coordinate origin in PCA plots, reflecting their role as a homogeneous mixture of all samples (Fig. 3E). Beyond removing or flanking low-quality features based on variability, features can also be filtered based on missingness, commonly following the 80% filtering rule in metabolomics research (Wei et al, 2018; Bijlsma et al, 2006). Here, the missingness for each feature can be quantified across all samples (Bijlsma et al, 2006) or across samples on a per-condition basis (Wei et al, 2018), as in our case done for each cell line (Dataset EV3). From a biological standpoint, filtering out low-quality or highly missing features is essential to avoid skewing downstream analyses by inflating the importance of certain pathways dominated by unreliable measurements.

To prevent biases in metabolomics data caused by variations in sample loading or instrument performance, a common approach is to normalise each sample by its TIC (Wulff and Mitchell, 2018). This involves dividing each feature intensity by the sample’s TIC and scaling it by the mean ion count across all samples (Fig. 3F; Dataset EV3). However, users are cautioned against applying TIC normalisation to datasets with a small number of features, as this may lead to inaccuracies (Wulff and Mitchell, 2018). Exometabolomics experiments require additional normalisation to allow interpretation of metabolite consumption or release (Meiser and Frezza, 2024). Specifically, each feature is normalised using both media blanks and either a growth factor (to account for overall cell growth during the experiment) or growth rate (to capture variations in cell proliferation). This normalisation yields negative values when a metabolite is consumed from the media, and positive values when it is released by the cells into the culture medium. This is crucial to understand metabolite exchange with the extracellular environment and can be used to study metabolic pathway activity (Meiser and Frezza, 2024). Even though commonly used, MVI can impact and potentially skew biological interpretation. Hence, we opted to only provide MVI for values that are missing not at random (MNAR), where half-minimum MVI has been shown to perform best for metabolomics (Wei et al, 2018). By performing PCA, we can check for batch effects, which are technical variations that confound downstream analysis, by, e.g., colour coding for biological replicates (Fig. EV2C). Given the multitude of factors that can introduce batch effects and the challenges they pose when conflated with true biological signals (Han and Li, 2022), we decided not to include batch correction algorithms in MetaProViz and encourage the user to apply dedicated tools for this. Once the data processing and normalisation is complete, Hotelling’s T2 test (Hotelling, 1931) is used to detect outliers across the multidimensional space of PCA (Figs. 3G and EV2D,E). It is up to the user to remove any sample outliers, but we recommend removing major outliers identified in the filtering round 1 (Fig. 3G). Here, it can also be helpful to use biological information such as the experimental conditions of the cell lines in this example (Fig. 3G; Dataset EV3). In this case, it becomes clear that sample MS51-09, identified as a potential outlier in filtering round 2, clusters together with replicates of the same cell line, so we would opt not to remove it.

### Combination of exo- with intracellular metabolomics and prior knowledge to generate a mechanistic hypothesis

**Figure 4:**
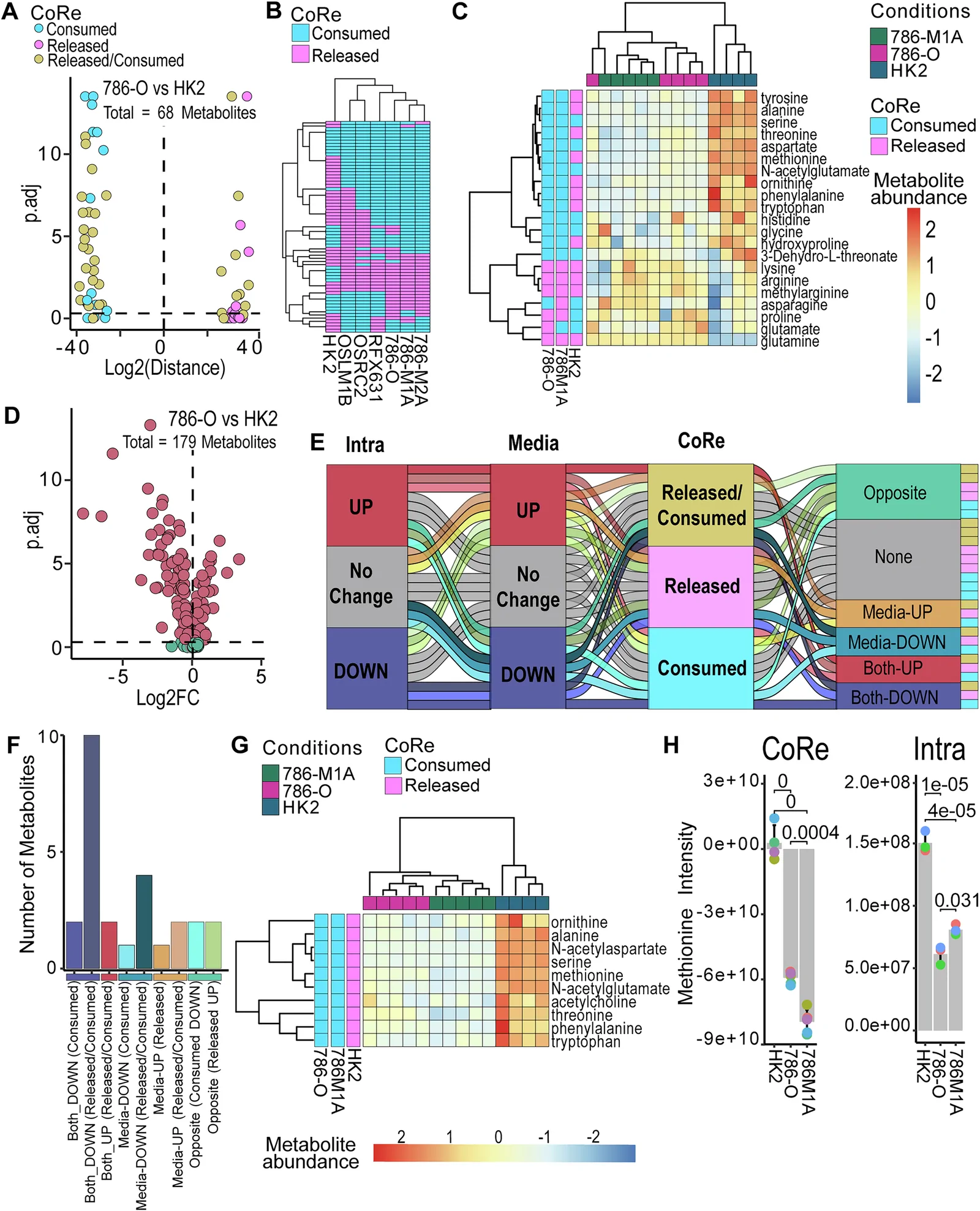
Combination of exo- with intracellular metabolomics and prior knowledge. (**A**) Differential metabolite analysis results for exometabolomics data comparing 786-O versus HK2 cells using ANOVA and false discovery rate (FDR) for *P* value adjustment (Shapiro–Wilk test: 96% (786-O)/82% (HK2) of the metabolites follow a normal distribution). (**B**) Heatmap of mean consumption-release of the measured metabolites across cell lines. (**C**) Heatmap of the biological replicates of the normalised ccRCC cell line exometabolomics data for the selected metabolites of amino acid metabolism for a sample subset. (**D**) Differential metabolite analysis results for intracellular data comparing 786-O versus HK2 cells using ANOVA and FDR for *P* value adjustment (Shapiro–Wilk test: 95% (786-O)/96% (HK2) of the metabolites follow a normal distribution). (**E**) Schematics of bioRCM process to integrate exometabolomics with intracellular metabolomics and (**F**) number of metabolites by their combined change patterns in intracellular- and exometabolomics in 786-M1A versus HK2. (**G**) Heatmap of the biological replicates of the cell lines metabolite abundances in the “Both_DOWN (Released/Comsumed)” cluster. (**H**) Bar graphs of normalised methionine intensity for exometabolomics with *n* = 5 biological replicates per condition (CoRe: negative value, if the metabolite has been consumed from the media, or a positive value, if the metabolite has been released from the cell into the culture media) and intracellular metabolomics with *n* = 3 biological replicates per condition (Intra). Dots represent biological replicates, and *P*-adjusted values were calculated using ANOVA and FDR. Bars represent the mean, and error bars indicate ± SD. Source data are available online for this figure.

To harness the full potential of exometabolomics data and unravel metabolic dysregulation, MetaProViz offers a suite of functional analysis tools that use differential metabolite analysis results as input. Starting from the normalised data matrix (normalisation details see “Methods” section and Fig. 3), we performed differential metabolite analysis using MetaProViz ccRCC cell line example data (Fig. 3A). Since exometabolomics data normalised to culture media blanks includes both positive and negative values, reflecting metabolite release and consumption, respectively, classical log2 fold change can not be applied. Instead, we calculate the log2 distance (Fig. 4A, “Methods” section), which captures whether a metabolite is released or consumed in both conditions, or released in one and consumed in the other (and vice versa). This information is visualised using a colour-coded scheme (Fig. 4A; Dataset EV3). For statistical testing, we used limma (Ritchie et al, 2015) and one-versus-all comparisons with *P* value adjustment using false discovery rate (FDR). To interpret exometabolomics data of multiple conditions, we generate feature metadata that summarises the consumption-release information based on the mean of the measured replicates per condition. These summaries are visualised in a heatmap, which displays cell line clustering according to their metabolite consumption-release profiles (Fig. 4B). To aid interpretation, we selected a representative subset of cell lines: healthy (HK2), primary ccRCC (786-O), and metastatic ccRCC (786-M1A), and selected metabolites based on their assigned primary pathway. Focusing on amino acid metabolism, we found that primary and metastatic ccRCC cell lines (786-O and 786-M1A, respectively) predominantly consume various amino acids, whilst healthy cells (HK2) produce and release those to the media (Fig. 4C).

Alongside exometabolomics experiments, intracellular metabolomics of the same cells is often performed. If both exometabolomics and intracellular metabolomics have been performed, MetaProViz offers functional clustering to integrate those data modalities. In MetaProViz, we provide intracellular metabolomics for four of the seven cell lines (Fig. 3A), namely HK2, 786-O, 786-M1A and 786-M2A. To integrate exometabolomics with intracellular metabolomics, differential metabolite analysis results from the same comparison are required (Fig. 4A,D; Dataset EV4). These results serve as input for our biological regulatory clustering method (bioRCM), which is based on logical regulatory rules. We have previously demonstrated that bioRCM can extract meaningful feature clusters in a multi-omics context (Rogerson et al, 2023; Mora et al, 2024). In MetaProViz, we created logical regulatory rules to integrate exometabolomics with intracellular metabolomics, consisting of an ordered series of three 5-state-5-state-4-state transitions. The first layer includes the intracellular metabolomics, the second layer the exometabolomics and the third layer the direction (consumed, released, consumed/released or not detected) of the exometabolites (Fig. 4E; Dataset EV3, sheet 13). This will result in clusters of metabolites that carry biological meaning (Fig. 4F; Dataset EV3, sheet 15). Next, we focused on the cluster “Both_DOWN (Released-Consumed)” and found that several amino acids are consumed by the ccRCC cell line 786-M1A but released by healthy HK2 cells. At the same time, intracellular levels are significantly lower than in HK2 (Log2FC = -0.9, *P*.adj = 4.4e-5) (Fig. 4G). To explore the role of these metabolites in signalling, we queried the prior knowledge resource MetalinksDB (Farr et al, 2024), which includes metabolite-receptor, metabolite-transporter and metabolite-enzyme relationships, for their known upstream and downstream protein interactors for the measured metabolites (Dataset EV3). This approach is especially valuable for exometabolomics, as it allows us to generate hypotheses about cell–cell communication. Notably, we identified links involving methionine (Fig. 4H), enzymes such as *BHMT*, and transporters such as *SLC43A2* that were previously shown to be important in ccRCC (Qu et al, 2022; Mora et al, 2024) (Dataset EV3). Interestingly, intracellular methionine levels comparing tumour versus normal tissue are decreased (Fig. 4H, right), and in the extracellular data, methionine was consumed from the media whilst control cells released methionine (Fig. 4H, left). This increased methionine usage in ccRCC could be explained by the increased DNA-hypermethylation landscape, a known characteristic in ccRCC (Clark et al, 2019).

In summary, calculating consumption-release and combining it with intracellular metabolomics via biological regulated clustering reveals metabolites of interest. Further combining these results with prior knowledge using the MetaproViz toolkit facilitates biological interpretation of the data.

### Understand metabolic alterations by patient metadata analysis and multi-condition clustering

**Figure 5:**
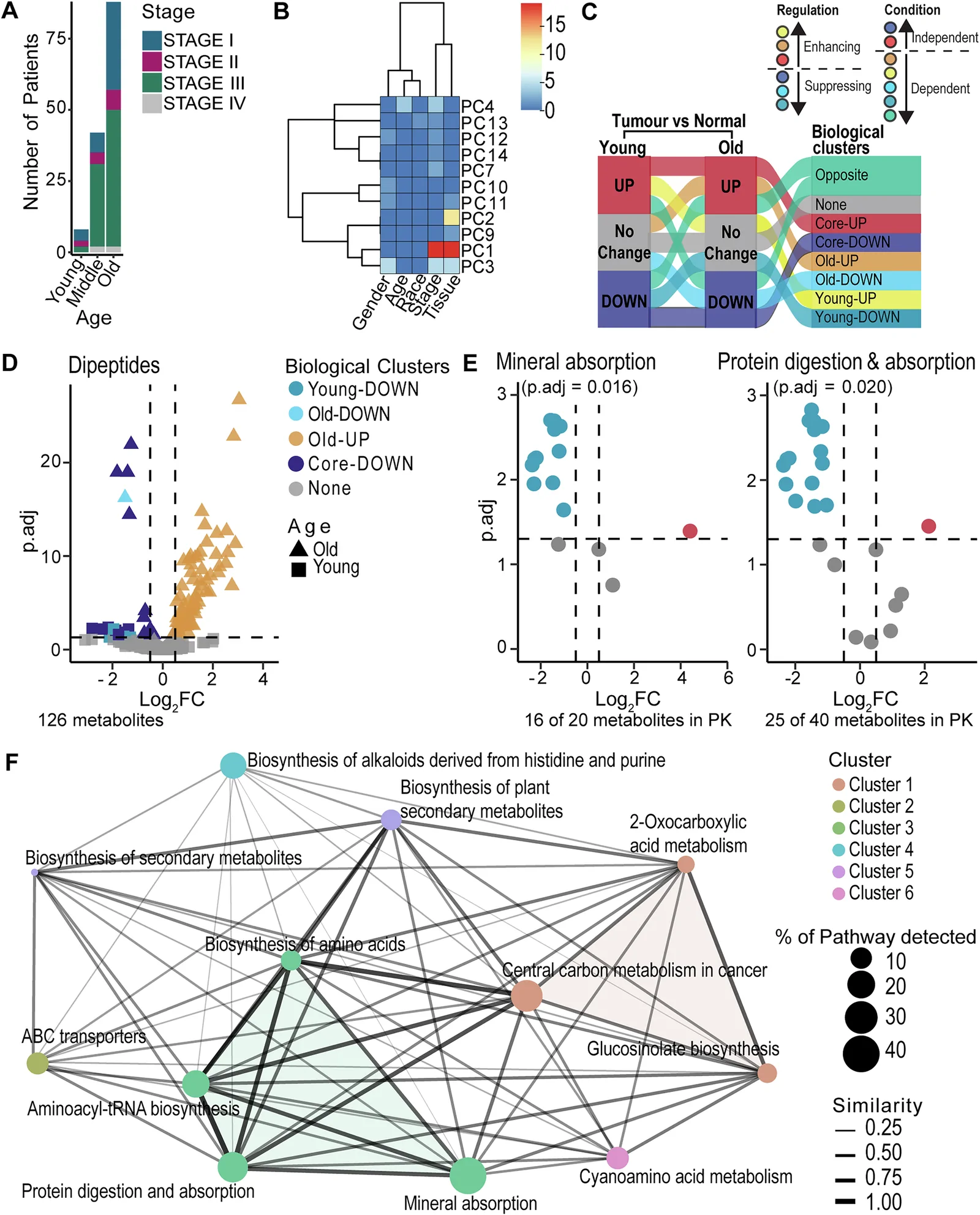
Patient metadata analysis and multi-condition clustering to dissect patient data. (**A**) Number of patients in the ccRCC cohort by age at surgery <42 (young), >42 & <58 (middle) and >58 (old) and tumour stage. (**B**) Heatmap of variance in principal components (PCs, *y* axis) explained significantly by patient metadata (*x* axis) (Tukey’s post doc test after anova *P*.adj < 0.05). (**C**) Schematics of two condition bioRCM processes to compare changes in tumour versus normal in old versus young patients. It results in biological clusters that carry information about regulation and condition dependence (colours). (**D**) Volcano plot of tumour versus normal metabolite changes in old (triangle, *n* = 88 patients) and young (square, *n* = 8 patients) ccRCC patients, colour-coded by biological clusters using limma and false discovery rate (FDR) for *P* value adjustment. (**E**) Volcano plots of tumour versus normal metabolite changes in young patients (*n* = 8 patients) (limma and FDR for *P* value adjustment) within two KEGG pathways that were significantly deregulated based on over-representation analysis. (**F**) Network graph of top enriched pathways (*P*.adjusted <0.05) of tumour vs normal in young patients, generated using MetaProViz cluster_pk() function. We used Jaccard similarity metrics, a similarity threshold of 0.6 and Louvain community detection for clustering. Node sizes represent the percentage of metabolites detected in each enriched pathway, and pathway similarity defines edge weights. Source data are available online for this figure.

Compared to cell line experiments, patient cohorts are generally more heterogeneous and come with extensive sample metadata (e.g., age, gender, therapeutic response or stage, amongst many other information), which is also the case for the ccRCC patient data in MetaProViz (Fig. 5A). To identify the main metabolite drivers, we created a method in MetaProViz that relates metabolite features to patient metadata. First, this method orders patients by a PCA on their metabolomics features, then it performs multiple ANOVA’s to quantify how much each principal component (PC) can be explained by each metadata variable. Applying this to the patient ccRCC data, as expected, tissue type (tumour or normal) was the strongest explanatory variable for PC1 (Fig. 5B; Dataset EV2). Another variable explaining the same amount of variance in PC1 is the tumour stage, which could point to adjacent normal tissue metabolic rewiring that happens in relation to stage and showcases that biological data harbour co-variations, which can not be disentangled by this method. Interestingly, the variance in PC4 can be explained in 3.8% by metabolites separating age and stage. We decided to dissect this further by applying bioRCM using the multi-condition clustering, focusing on patient subsets of young (age <42 years) and old (age >58 years) patients (Fig. 5C). The bioRCM we developed to interpret multi-condition experiments uses an ordered series of two 5-state-5-state transitions between the layers. The core states include “UP”, “DOWN” and “No Change” with the latter further subdivided into “Not detected”, “No significant change”, “Significant positive” and “Significant negative” (Dataset EV2). One of the most altered metabolite classes are dipeptides that could play a role as biomarkers in cancer (Ozawa et al, 2020) and are upregulated in old patients, whilst downregulated or unchanged in young patients (Fig. 5D). Enrichment analysis using KEGG pathways further revealed that “Mineral absorption” and “Protein digestion and absorption” are specifically downregulated in young patients (Fig. 5E; Dataset EV2). Moreover, those signatures cluster together with “Biosynthesis of amino acids” and “Aminoacyl-tRNA iosynthesis” based on common metabolites present in the KEGG pathways (Fig. 5F). Together, this potentially explains the reduced number of dipeptides observed.

As this example shows, performing metadata-driven analysis can extract metabolic patterns of patient subgroups, which classical pathway enrichment analysis would not have been able to extract.

## Discussion

MetaProViz is a modular, flexible, open-source and reproducible metabolomics analysis package that integrates curated prior knowledge with a rich functional analysis toolkit. Its capabilities extend beyond standard workflows by addressing key limitations in the field arising from the ambiguity of metabolite features and the mapping of metabolite identifiers across databases. MetaProViz achieves this through dedicated tools that complete feature annotations with missing identifiers and quantify mapping ambiguities, facilitating a more accurate and comprehensive integration of metabolomics data with existing knowledge. Ultimately, this improves pathway enrichment and functional analyses, surpassing the identifier handling and enrichment options available in other metabolomics analysis solutions (Pang et al, 2024). Besides intracellular metabolomics, MetaProViz also includes dedicated methods for exometabolomics analysis, which, to our knowledge, no other publicly available tool currently provides. These methods enable the systematic analysis of metabolite consumption and release by cells and linking extracellular metabolic changes to intracellular pathways and mechanisms, expanding the scope of functional and mechanistic interpretation. Combined with visualisation features designed to minimise the risk of misinterpretation, MetaProViz supports the generation of mechanistic hypotheses and strengthens the biological interpretability of metabolomics studies.

The integration of metabolomics data with prior knowledge remains challenging due to ambiguity in feature identification and ambiguous mapping between ID types. There is no single solution that fits all problems; therefore, MetaProViz offers a set of functions to expand the metabolite ID space within datasets, translates IDs of data or metabolite sets, quantifies mapping ambiguities, and highlights problematic cases. We anticipate that future advances will move beyond strict reliance on database-specific identifiers and leverage chemical structures and ontologies to explicitly resolve ambiguities, thus further improving mapping accuracy and interpretability. MetaProViz is already structured to accommodate such developments by using the OmniPath ecosystem (Turei et al, 2021) at the backend, which enables us to address these limitations inherent to current ID-based methods in the future.

MetaProViz inclusion of prior knowledge of receptors, transporters and other protein interactors, provided by MetalinksDB, extends analysis beyond classical pathways by linking altered metabolite levels to signalling cascades. Applying this to clear-cell renal cell carcinoma (ccRCC) data, we identified increased methionine usage in ccRCC cell lines, and through MetalinksDB, linked this change to enzymes and transporters associated with overall survival (Qu et al, 2022; Mora et al, 2024). Consistent with these findings, patient tumour samples exhibited reduced methionine levels compared to normal tissue, potentially reflecting the elevated DNA-hypermethylation landscape characteristic of ccRCC (Clark et al, 2019). This case study illustrates the potential of MetaProViz’ to integrate metabolomics data with prior knowledge, to generate biologically meaningful hypotheses, and to place metabolic changes into a broader mechanistic context. In addition, providing knowledge of receptor-, transporter- and enzyme-metabolite pairs, MetaProViz can readily accommodate transcriptomics and proteomics data for combined enrichment analysis.

Metabolomics poses unique analytical challenges compared to genomics, and we designed MetaProViz to actively guide users in selecting appropriate methods. Significant development effort has gone into implementing informative messages and automated checks, for example, performing Shapiro–Wilk tests during differential analysis to report whether the chosen statistical test is appropriate. These safeguards help prevent common analytical pitfalls and support informed decision-making for both experienced bioinformaticians and researchers less familiar with metabolomics-specific issues. At the same time, the modular architecture of MetaProViz supports integration with complementary tools, such as COSMOS (Dugourd et al, 2021) for multi-omics network analysis, and facilitates future extensions for single-cell and spatial metabolomics. Incorporation into Bioconductor (Huber et al, 2015) (https://bioconductor.org/packages/devel/bioc/html/MetaProViz.html) already supports reproducible and scalable applications, whilst future incorporation into workflow management systems, such as nf-core (Ewels et al, 2020) modules in Nextflow (Di Tommaso et al, 2017), will further enhance the usage in metabolomics core facilities. Beyond current capabilities, future developments could also incorporate mechanistic modelling to capture metabolic fluxes, subcellular compartmentalisation, enzyme kinetics, regulatory feedback loops, and thermodynamic constraints to dissect metabolic response under perturbations. Given the imminent shift in metabolite identification through AI-based approaches, including language model-guided (Qiang et al, 2026) methods and self-supervised learning (Bushuiev et al, 2025), the growing number of identified metabolites will make the MetaProViz toolkit increasingly valuable for the community to gain functional insights.

In summary, MetaProViz combines prior knowledge access, advanced identifier translation, and mapping ambiguity quantification with exometabolomics analysis, functional analysis, and visualisation within a flexible and interoperable framework. Its unique support for exometabolomics, together with safeguards such as automated checks and informative messages, addresses common pitfalls and improves decision-making during analysis. Its design balances the needs of bioinformaticians accustomed to genomic workflows but new to metabolomics, while also enabling wet-lab scientists with minimal coding experience to perform robust and reproducible analyses.

## Methods

**Table:** Reagents and tools table

Reagent/resource	Reference or source	Identifier or catalogue number
**Software**		
**MetaProViz dependency** R-package name		
dplyr	https://dplyr.tidyverse.org	CRAN; dplyr 1.2.1
ggfortify	https://github.com/sinhrks/ggfortify	CRAN; ggfortify 0.4.19
magrittr	https://magrittr.tidyverse.org	CRAN; magrittr 2.0.5
purrr	https://purrr.tidyverse.org/	CRAN; purrr 1.2.2
rlang	https://rlang.r-lib.org	CRAN; rlang 1.2.0
stringr	https://stringr.tidyverse.org	CRAN; stringr 1.6.0
tibble	https://tibble.tidyverse.org/	CRAN; tibble 3.3.1
tidyr	https://tidyr.tidyverse.org	CRAN; tidyr 1.3.2
ComplexUpset	https://github.com/krassowski/complex-upset	CRAN; ComplexUpset 1.3.3
DBI	https://dbi.r-dbi.org	CRAN; DBI 1.2.3
EnhancedVolcano	https://github.com/kevinblighe/EnhancedVolcano	Bioconductor 3.20; EnhancedVolcano 1.24.0
OmnipathR	https://github.com/saezlab/OmnipathR	GitHub; saezlab/OmnipathR 3.19.12
Polychrome	http://oompa.r-forge.r-project.org/	CRAN; Polychrome 1.5.4
S4Vectors	https://bioconductor.org/packages/S4Vectors	Bioconductor 3.20; S4Vectors 0.44.0
broom	https://broom.tidymodels.org/	CRAN; broom 1.0.10
cosmosR	https://github.com/saezlab/COSMOSR	Bioconductor 3.20; cosmosR 1.14.0
factoextra	http://www.sthda.com/english/rpkgs/factoextra	CRAN; factoextra 1.0.7
ggVennDiagram	https://github.com/gaospecial/ggVennDiagram	CRAN; ggVennDiagram 1.5.2
ggbeeswarm	https://github.com/eclarke/ggbeeswarm	CRAN; ggbeeswarm 0.7.2
ggplot2	https://ggplot2.tidyverse.org	CRAN; ggplot2 4.0.2
ggpubr	https://rpkgs.datanovia.com/ggpubr/	CRAN; ggpubr 0.6.1
ggrepel	https://ggrepel.slowkow.com/	CRAN; ggrepel 0.9.6
gridExtra	https://cran.r-project.org/package=gridExtra	CRAN; gridExtra 2.3
gtools	https://github.com/r-gregmisc/gtools	CRAN; gtools 3.9.5
hash	http://www.johnhughes.org	CRAN; hash 2.2.6.3
igraph	https://r.igraph.org/	CRAN; igraph 2.2.1
inflection	https://CRAN.R-project.org/package=inflection	CRAN; inflection 1.3.7
kableExtra	http://haozhu233.github.io/kableExtra/	CRAN; kableExtra 1.4.0
knitr	https://yihui.org/knitr/	CRAN; knitr 1.50
limma	https://bioinf.wehi.edu.au/limma/	Git; limma 3.65.5
logger	https://daroczig.github.io/logger/	CRAN; logger 0.4.1
patchwork	https://patchwork.data-imaginist.com	CRAN; patchwork 1.3.2
pheatmap	https://cran.r-project.org/package=pheatmap	CRAN; pheatmap 1.0.13
qcc	https://github.com/luca-scr/qcc	CRAN; qcc 2.7
qvalue	http://github.com/jdstorey/qvalue	Bioconductor 3.20; qvalue 2.38.0
rappdirs	https://rappdirs.r-lib.org	CRAN; rappdirs 0.3.3
readr	https://readr.tidyverse.org	CRAN; readr 2.1.6
rstatix	https://rpkgs.datanovia.com/rstatix/	CRAN; rstatix 0.7.2
svglite	https://svglite.r-lib.org	CRAN; svglite 2.2.1
tidyselect	https://tidyselect.r-lib.org	CRAN; tidyselect 1.2.1
writexl	https://ropensci.r-universe.dev/writexl https://docs.ropensci.org/writexl/	CRAN; writexl 1.5.4
SummarizedExperiment	https://bioconductor.org/packages/SummarizedExperiment	Bioconductor 3.20; SummarizedExperiment 1.36.0
**Additionally used for paper analysis** R-package name		
MetaProViz	https://github.com/saezlab/MetaProViz	GitHub; saezlab/MetaProViz 3.99.53
easyalluvial	https://github.com/erblast/easyalluvial/	CRAN; easyalluvial 0.3.2
rmarkdown	https://github.com/rstudio/rmarkdown	CRAN; rmarkdown 2.31
tidyverse	https://tidyverse.tidyverse.org	CRAN; tidyverse 2.0.0
R.methodsS3	https://github.com/HenrikBengtsson/R.methodsS3	CRAN; R.methodsS3 1.8.2
R.oo	https://henrikbengtsson.github.io/R.oo/	CRAN; R.oo 1.27.1
R.utils	https://henrikbengtsson.github.io/R.utils/	CRAN; R.utils 2.13.0
lazyeval	https://cran.r-project.org/package=lazyeval	CRAN; lazyeval 0.2.3

### Example data

MetaProViz provides access to diverse example datasets from kidney cancer studies. Cell-line data include intracellular metabolomics of human renal epithelial cells (HK2) and renal cell carcinoma (ccRCC) cell lines 786-O, 786-M1A, and 786-M2A and corresponding cell culture media profiling (exometabolomics), which additionally includes ccRCC cell lines OS-RC-2, OS-LM1, and RFX-631 were downloaded from the metabolomics workbench project PR001418, including study ST002224 and ST002226 (Data ref: Yang, 2022; Sciacovelli et al, 2022). Patient tissue metabolomics from 138 matched ccRCC and adjacent normal tissue pairs were downloaded from Hakimi et al's “supplementary table 2”, which includes median normalised data (Hakimi et al, 2016). Patient tissue processed transcriptomics and proteomics data from ccRCC tissue were downloaded from Mora and Schmidt et al's table “13073_2024_1415_MOESM3_ESM.xlsx” originally from proteomics Data Commons accession number PDC000127 (Clark et al, 2019; Mora et al, 2024; Data ref: Clark et al, 2019). The detailed code can be found on the “ExampleData” branch of the MetaProViz package (https://github.com/saezlab/MetaProViz/tree/ExampleData).

### Data cleaning

To exclude measured features of low quality, the missingness for each feature across all samples per-condition was quantified (modified 80%-filtering rule) (Wei et al, 2018) and features with missingness <= 20% were kept. In addition, features with CV > 30% in the pool samples (homogenous mixture of all samples) were removed. Lastly, for exometabolomics features with CV > 30 in media blanks (media without cells) were flanked.

### Data processing

TIC normalisation according to Wulff et al (Wulff and Mitchell, 2018) was performed by dividing feature intensities by the sample’s TIC and scaling by the mean ion count across all samples and thus ensures that variations in sample loading or instrumentation do not bias the data. For the exometabolomics experiments media sample (cell culture media taken from cells) were additionally normalised using both media blanks (samples where no cells were cultured in) and growth factor (accounts for cell growth during experiment) as growth rate (accounts for variations in cell proliferation) has not been available.

1 $${MA}({S}_{1})=\left({S}_{1}-\mu \left(B\right)\right)* {G}_{1}$$

- MA represents the metabolite abundance (arbitrary unit) of a measured metabolite.
- *S*_1_ represents sample 1.
- μ(*B*) represents the population mean of the blank samples.
- G_1_ represents the growth factor or growth rate corresponding to S_1_.

The results are either a negative value, if the metabolite has been consumed from the media, or a positive value, if the metabolite has been released from the cell into the culture media.

### MVI

For cell-line data, we performed half-minimum (HM) MVI per feature assuming missing values that are missing not at random (MNAR) for which HM has been shown to perform best (Wei et al, 2018). Notably, MetaProViz does not offer other ways to perform MVI and urges the user to use dedicated tools for this.

### Outlier detection

Testing for outliers was performed using Hotelling’s T2 outlier test (Hotelling, 1931) with a confidence interval of 0.99. If an outlier was identified, it was removed, and the test was repeated. All results can be found in the supplementary tables. Based on this, we removed sample “MS51-06” (biological replicate of HK2 cell line) from the exometabolomics dataset (Sciacovelli et al, 2022) and sample “MS55_29” (analytical replicate of biological replicate 2 of 786-M2A cell line) from the intracellular dataset (Sciacovelli et al, 2022). No samples were removed from the patient dataset (Hakimi et al, 2016).

### Differential metabolite analysis (DMA)

For statistical testing, pairwise comparisons of conditions were defined by the metadata annotations and passed using the numerator and denominator. For cell lines, we performed one-vs-all comparisons (HK2 cells versus all other cells) using ANOVA. For the patient’s ccRCC data, we compared tumour versus normal of two patient subsets, “young” (< 42 years) and “old” (> 58 years). In general, MetaProViz supports all-vs-all one-vs-all and one-vs-one comparisons. For the latter, methods including Student’s *t* test, Wilcoxon rank-sum test (Wilcoxon, 1945), chi-squared test, or correlation analysis can be used. For one-vs-all or all-vs-all comparisons, methods such as ANOVA, Welch’s ANOVA (Welch, 1951), Kruskal-Wallis test (Padhee and Ahmed, 2024), or linear modelling (via limma’s lmFit method (Ritchie et al, 2015)) are available. To account for multiple hypothesis testing, *P* values were adjusted using the FDR (Rouam, 2013) method unless otherwise specified. Yet, MetaProViz also offers other adjustment methods such as Benjamini–Hochberg (BH) (Haynes, 2013). Assessment of statistical assumptions was done for each metabolite by checking the normality of metabolite distributions across conditions using the Shapiro–Wilk test (Padhee and Ahmed, 2024) and by evaluating the homogeneity of variances using Bartlett’s test (Viwatwongkasem et al, 2004). For “Standard DMA” applied to intracellular metabolomics, the Log2 Fold Change (Log2FC) comparing Condition 1 (C_1_) versus Condition 2 (C_2_) is calculated based on the mean (average) of the sample values corresponding to the same condition C_n_:

2 $${C}_{x}=\frac{1}{n}{\sum }_{i=1}^{n}{S}_{i}$$

- C_x_ represents the condition a set of samples (S_i_) corresponds to.
- $\frac{1}{n}$ represents the reciprocal of the total number of values (n), which calculates the mean.
- ${\sum }_{i=1}^{n}{S}_{i}$ represents the summation of all the sample values, from S_1_ to S_n_.

Log2 Fold Change (Log2FC) comparing Condition 1 (C1) versus Condition 2 (C2)

3 $${Log}2{FC}\left(\frac{{MA}\left({C}_{1}\right)}{{MA}\left({C}_{2}\right)}\right)=log 2\left(\frac{{C}_{1}}{{C}_{2}}\right)$$

- ${Log}2{FC}(\frac{{MA}({C}_{1})}{{MA}({C}_{2})})$ represents the Log2FC comparing the MA of C_1_ versus the MA of C_2_.

Statistical results of hypothesis testing for the difference between the individual sample MA values of C_1_ and the individual sample MA values of C_2_ is calculated using standard statistical tests as described above. For “CoRe DMA”, which is applied to exometabolomics, the absolute difference between C_1_ versus C_2_ was calculated by first calculating the mean (average) of the sample values corresponding to the same condition C_n_ using (1). In the next step, we used C_x_ to calculate the absolute difference. In this step, the absolute difference is log_2_-transformed to make the values comparable between the different metabolites.

4 $$\log 2[{{\rm{abs}}}\left(\right.({{\rm{C}}}1)-({{\rm{C}}}2)]$$

The result does not consider whether one product is larger than the other; it only looks at the magnitude of their difference. To reflect the direction of change between the two conditions, we multiplied by −1 if C1 < C2.

### Prior knowledge access

MetaProViz uses the OmniPath database and the R package OmnipathR to access prior knowledge (Turei et al, 2021). To support the applications in MetaProViz, we implemented new clients in OmnipathR for HMDB (Wishart et al, 2007, 2022), RaMP DB (Braisted et al, 2023), KEGG (Kanehisa et al, 2017), Hallmarks (Liberzon et al, 2015), MetalinksDB (Farr et al, 2024), Gaude (Gaude and Frezza, 2016), Reactome (Milacic et al, 2024), Wikipathways (Agrawal et al, 2024) and MacDB (Sun et al, 2023). These clients import data directly from the original source in bulk and parse it into data frames. OmnipathR provides infrastructure for the clients, such as download management and local cache.

### ID quality control

We assess the compatibility of all metabolite IDs originally assigned to each feature to identify potential mismatches early in the workflow. For this, we build a bidirectional ID edge table from pairwise RaMP (Braisted et al, 2023) ID mapping tables, retrieved through OmnipathR (Turei et al, 2021) for HMDB, KEGG, ChEBI, and PubChem. The initially assigned IDs to a feature are treated as seed IDs. For each feature, we evaluate all distinct seed ID pairs and determine whether the two IDs are connected in the ID edge graph, either by a direct match or through intermediate IDs. Seed ID pairs are considered compatible when such a connection exists, and incompatible when no direct or indirect connection is found. We summarise the resulting compatibility assessments at the feature level by flagging whether all assigned seed IDs are mutually compatible, and we manually resolve flagged mismatches. This step helps prevent the propagation of incorrect feature annotations in downstream analyses.

### Metabolite ID translation and addition

In MetaProViz, we make use of OmnipathR’s (Turei et al, 2021) identifier translation utility, which is able to translate between all compound ID types supported by RaMP (Braisted et al, 2023) or HMDB (Wishart et al, 2007, 2022), in addition to several protein, gene and RNA ID types. We also use this translation service to add additional potential metabolite IDs by one-step direct mappings in combination with a manually curated amino acid table. The translation is highly customisable, and optionally an ambiguity analysis is carried out, qualifying and quantifying the one-to-one, one-to-many and many-to-many mappings in the data frame, which is summarised in the summary report. If grouping variables such as pathway names in pathway-metabolite sets are present, ambiguities within and across groups were also analysed and quantified.

### ID traversal

To increase the feature-level ID space, we expand the initial metabolite ID set of each feature by graph traversal. This function first applies the ID quality control method’s ID compatibility assessment and constructs a bidirectional ID edge table from pairwise RaMP (Braisted et al, 2023) ID mapping tables, as described in the ID quality control method. For each feature, the initially assigned IDs are used as starting points (seed IDs). In a second step, all connected IDs are collected by traversing the same ID graph from the seed IDs until no additional IDs are found. This expands the feature-level ID space across multiple identifier systems and captures both direct and indirect relationships. We quantify expanded IDs per type and include quality flags that indicate large expansions and potential seed-ID mismatches, enabling transparent downstream interpretation and decision-making.

### Prior knowledge refactoring and contextualisation

To combine the exometabolomics data with MetalinksDB (Farr et al, 2024), we contextualised MetalinksDB to only contain cell_location = “Extracellular”, tissue_location = c(“Kidney”, “All Tissues”) and biospecimen_location = c(“Blood”, “Urine”). Furthermore, we excluded all metabolites that were not part of the bioRCM cluster RG2_Significant “Both_DOWN (Released/Consumed)” (see biological regulatory clustering method for details on clustering).

### Biological regulatory clustering model (bioRCM)

This is conceptually based on the Signature Regulatory Clustering (SiRCle) (Mora et al, 2024), and the input data are differential metabolite analysis results, including statistics and fold change or distance and direction for exometabolomics data. Depending on the input data available, there are two bioRCM methods available: Multi-Condition bioRCM and Consumption-Release bioRCM. For Multi-Condition bioRCM multiple experimental conditions or patient metainformation have to be available that can be used to create multiple comparisons. Here we performed two differential analyses comparing tumour versus normal, first for a subset of old patients (age at surgery >58) and second for a subset of young patients (age at surgery <42) of the patient’s example data, which was used as input for the bioRCM Multi-Condition clustering. For Consumption-Release bioRCM intracellular and exometabolomics under the same conditions are required. Here, we performed differential analysis comparing 786-M1A versus HK2 cells using the intracellular and exometabolomics cell line example data.

The user will need to choose the “background method (BG)”, the thresholds/cutoffs of the input data and the “Regulation Grouping (RG)”. The BG setting defines which features are considered for the bioRCM clusters (Table 1). For example, Intra&CoRe is the most restrictive setting as only features detected in exometabolomics and intracellular metabolomics are considered. Here we have used the BG settings “C1&C2” for the multi-condition approach and “Intra&CoRe” for the Consumption-Release approach.

**Table 1:** Overview of bioRCM background (BG) options.

bioRCM	BG	Description
Multi-Condition	C1|C2	Features detected in Comparison 1 OR Comparison 2
	C1&C2	Features detected in Comparison 1 AND Comparison 2
	C1	Features detected in Comparison 1
	C2	Features detected in Comparison 2
Consumption-Release	Intra|CoRe	The least stringent background method, since a metabolite will be included in the input if it has been detected on one of the two conditions.
	Intra&CoRe	The most stringent background setting, which leads to a small number of metabolites.
	Intra	Focus is on the metabolite abundance of the intracellular.
	CoRe	Focus is on the metabolite abundance of the CoRe.

Next, the user will set the different input thresholds, which will define whether a feature is considered “UP”, “DOWN” or “No Change”, with “UP”/“DOWN” meaning a feature is significantly up-/downregulated in the underlying comparison and “No Change” are features that do not meet the significance and/or the fold change/distance threshold, or dependent on the background (BG) setting were not detected. Hence, “No Change” is subdivided into “undetected”, which includes features detected in one comparison/one data layer, but not in the other comparison/data layer, “not significant”, which includes features that do not meet the significance threshold set, “significant negative”, and “significant positive”, which includes features that do not meet the negative/positive fold change threshold but meet the significance threshold.

Lastly, the “Regulation Grouping (RG)” defines how the different flows will be summarised into clusters, whereby all flows can be found in RG1_All. The Multi-Condition bioRCM uses an ordered series of two 5-state-5-state transitions between the two layers ( = comparisons), where the five states divide the metabolites into upregulated, downregulated, “significant negative”, “significant positive”, “undetected”. Similarly, also the Consumption-Release bioRCM includes these 5-state-5-state transitions for intracellular and exometabolomics, but additionally extends to a 5-state-5-state-4-state between the layers where the first layer includes the intracellular metabolomics, the second layer the exometabolomics and the third layer the direction (consumed, released, consumed/released or not detected) of the exometabolites. Those flows are summarised either with a focus on significant changes, meaning even metabolites that are UP, and significant positive will be placed in the same cluster, or with a focus on the change, meaning in this case UP and significant positive will not be placed in the same cluster. For the exact flows, also check Datasets EV3 and  EV4.

### Over-representation analysis

We perform over-representation analysis (ORA), which is based on the Fisher’s exact test, either on the top/bottom 10% of the ranked (t.value) differential metabolite analysis results or on the clusters from bioRCM. In both cases, we set all detected metabolites as the background.

### Metabolite set clustering

We implemented a customisable clustering function to analyse overlapping metabolites across metabolite sets to quantify and visualise redundancy and similarity among metabolite sets and applied it to the ORA enrichment results. In detail, we compute pairwise metabolite set similarity using the overlap-based Jaccard similarity metrics with a threshold of 0.6 for the similarity to remove weakly related pairs of metabolite sets (similarities < threshold are set to 0). To group the metabolite sets, we cluster the similarity results using community-based (Louvain) (Blondel et al, 2008) clustering. The user will need to choose a suitable similarity method with a threshold and the subsequent clustering method (Table 2). The results are visualised using network graphs, in which nodes represent metabolite set terms and weighted edges represent metabolite set similarity, enabling redundancy assessment, overlap analysis, and sanity checking of enrichment results and metabolite sets. This analysis can also be applied prior to enrichment analysis directly on the prior knowledge metabolite sets.

**Table 2:** Overview of the available similarity and clustering methods.

Method		Description
Similarity	Jaccard overlap (Jaccard, 1901)	Fraction of shared metabolites relative to the union.
	Overlap-coefficient (Kjos‑Hanssen, 2022) (Szymkiewicz-Simpson)	Fraction of shared metabolites relative to the smaller set.
	Correlation-based	Pearson/Spearman/Kendall, etc. correlation between binary term vectors; negatives set to 0.
Clustering	Connected-components	Clusters are connected components in the thresholded (unweighted) graph.
	Community-based (Louvain) (Blondel et al, 2008)	Louvain communities on the thresholded weighted graph.
	Hierarchical	Agglomerative clustering on thresholded distance matrix (1 − similarity), cut at 1 − threshold.

### Sample metadata analysis

First, we reduced all the measured features of one sample into a few features in the different principal components (PC) by performing PC analysis (PCA). Since each PC explains a certain percentage of the variance between the different samples, this enables interpretation of sample clustering based on the measured features. By performing an ANOVA between each PC and selected patient metainformation (tissue, stage, age, gender), we extracted the main metabolite drivers that separate patients based on these metainformation.

### Visualisation

MetaProViz fine-tunes the colour palettes, aesthetics, and sizes of elements within each figure layout, with the aim of consistently generating well-readable, publication-ready figures. For this, we use ggplot2-based (Wickham, 2016) figures and adapt figures from EnhancedVolcano (Blighe et al, 2018), pheatmap (Kolde, 2025), ComplexUpset (Lex et al, 2014) and igraph (Csárdi and Nepusz, 2006).

### Blinding

No blinding was performed in this study. This work is a secondary analysis of publicly available datasets, and no experimental allocation, intervention, or outcome assessment requiring blinding was conducted by the authors.

## Supplementary information

Appendix

Peer Review File

Dataset_EV1

Dataset_EV2

Dataset_EV3

Dataset_EV4

Source data Fig. 1

Source data Fig. 2

Source data Fig. 3

Source data Fig. 4

Source data Fig. 5

EV Figures 1 and 2 Source Data

Expanded View Figures

## Data Availability

Data generated as part of this study are available as Tables EV1 to EV4 or via GitHub: https://github.com/saezlab/MetaProViz/tree/BioRxiv. Information to download all used raw data from the respective studies is available on the “ExampleData” branch of the MetaProViz package (https://github.com/saezlab/MetaProViz/tree/ExampleData). The code, release history, reference documentation, and vignettes of the MetaProViz R-package is available at https://saezlab.github.io/MetaProViz/ and https://bioconductor.org/packages/devel/bioc/html/MetaProViz.html. The code to reproduce all figures and tables in this manuscript including the complete environment is available at https://github.com/saezlab/MetaProViz/tree/BioRxiv and https://github.com/saezlab/MetaProViz/tree/Revision. The source data of this paper are collected in the following database record: biostudies:S-SCDT-10_1038-S44320-026-00231-8.
