# Supplementary material for: Integrated metabolomics data analysis to generate mechanistic hypotheses with MetaProViz
Source: Mol Syst Biol. 2026 Aug 17;22(10):1605–25. doi: 10.1038/s44320-026-00231-8 (PMC13639010; doi:10.1038/s44320-026-00231-8)
Supplement: Supplementary file 1 [file 44320_2026_231_MOESM13_ESM.pdf]

Expanded View Figures

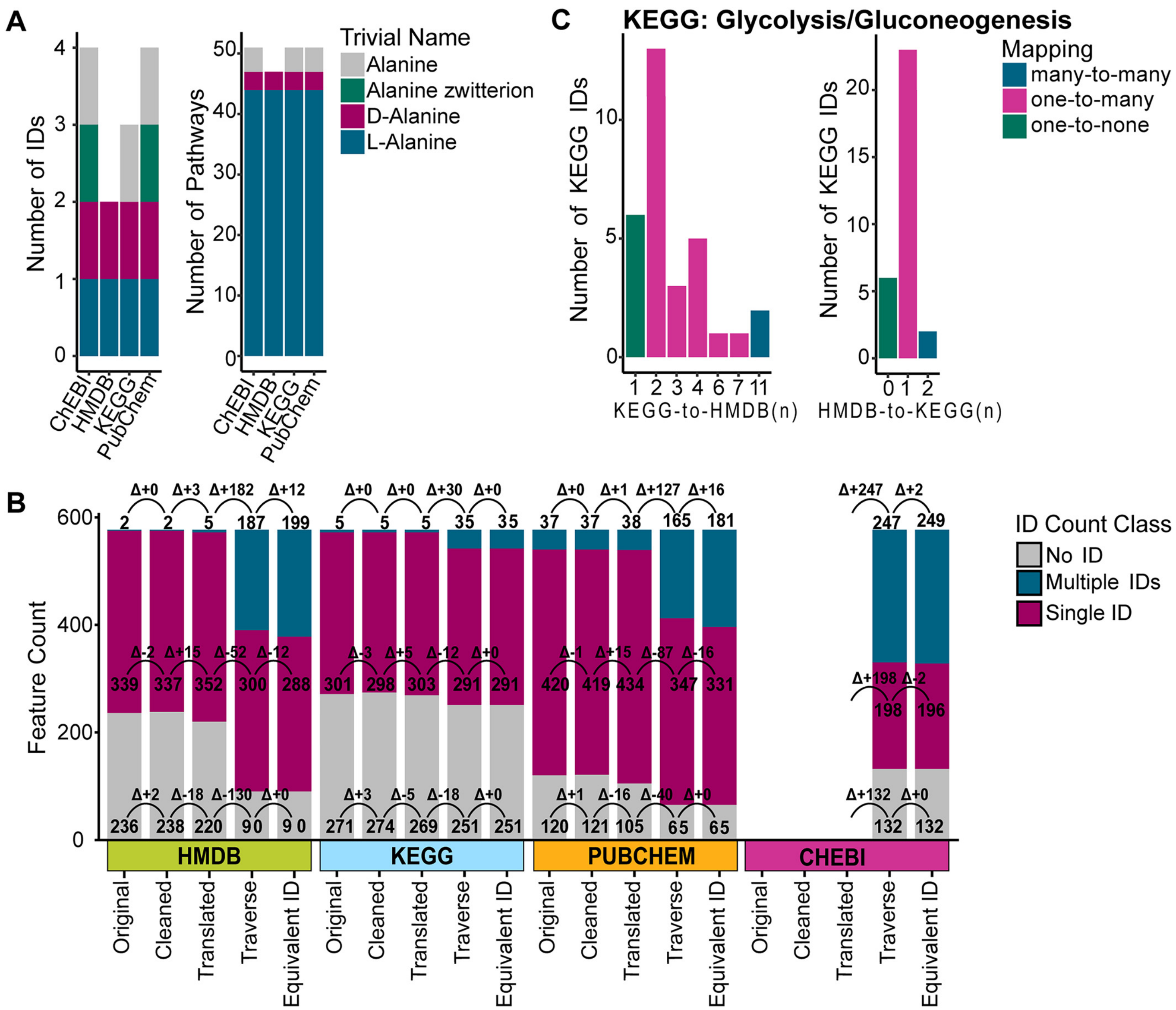

Figure EV1. ID mapping problems in metabolite prior knowledge.

(**A**) Example of the presence of multiple metabolite IDs for the amino acid alanine in ChEBI, HMDB, KEGG and PubChem databases (left) and the number of pathways these metabolite IDs can be mapped to by RaMP-DB (right). (**B**) Stacked bar graphs showing the frequency at which a certain number of metabolite IDs per integrated peak are available (no, one or multiple) with the number of feature counts (*y* axis) for HMDB, KEGG, PUBCHEM and CHEBI within the ccRCC patient feature space. "Original" is referring to the IDs provided by the original publication, "Cleaned" is after our ID mismatch check (= deltas are mostly negative), "Translated" are IDs that were added by translating from CAS numbers, "Traverse" are IDs added after using MetaProViz ID traversion method and "Equivalent ID" are IDs added based on stereoisomerism. The deltas describe the IDs gained through each step and show that over the course of the ID workflow, features without IDs (grey) are decreasing, whilst features with multiple IDs (blue) are increasing. (**C**) Ambiguity in the translation of metabolites in the "KEGG Glycolysis/Gluconeogenesis" pathway from KEGG to HMDB IDs (left) and then back (right) using MetaProViz; number of source IDs (bar heights) that map to a certain number of target IDs (*x* axis). Source data are available online for this figure.

 © EMBL-EBI and the Authors

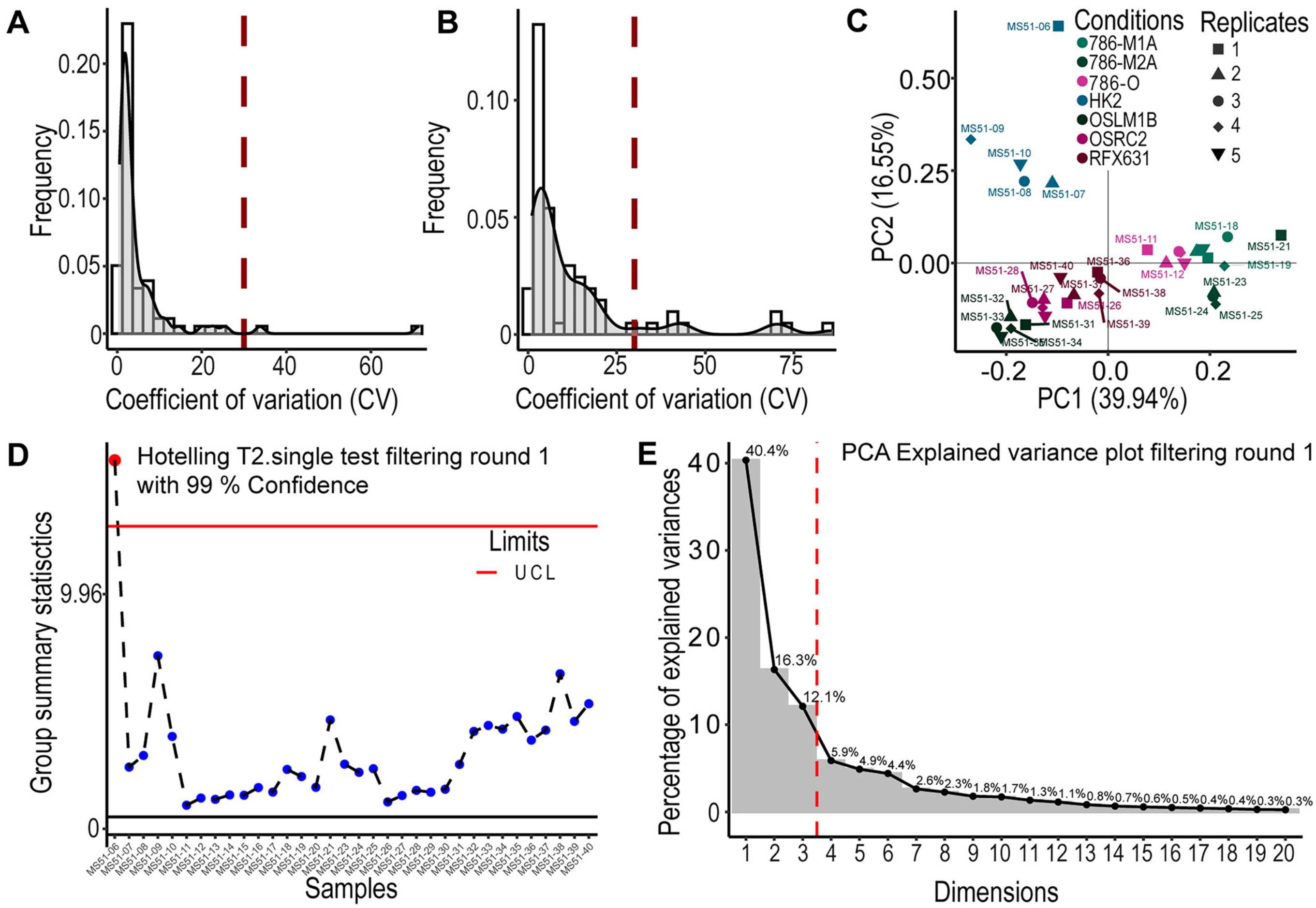

Figure EV2. Quality control and processing of exometabolomics data using MetaProViz.

Feature QC and outlier testing of exometabolomics data using MetaProViz. (**A**, **B**) Exometabolomics feature quality of (**A**) metabolite pool sample coefficient of variation (CV) ($n = 3$), (**B**) media blank samples ($n = 5$), which are samples where no cells were cultured in. (**C**) PCA plot with shapes for biological replicates to understand replicate spread. (**D**, **E**) Visualisation of different parameters of Hotelling's T2 test results with 99% confidence interval of filtering round 1 including (**D**) Group summary statistics highlighting MS51-06 as outlier, which was removed and (**E**) explained variance of PCA plot with elbow (red line). Detailed code, explanation and plots can be found in the vignette (https://saezlab.github.io/MetaProViz/articles/CoRe%20Metabolomics.html). Source data are available online for this figure.

© EMBL-EBI and the Authors
