# Supplementary material for: Integrated metabolomics data analysis to generate mechanistic hypotheses with MetaProViz
Source: Mol Syst Biol. 2026 Aug 17;22(10):1605–25. doi: 10.1038/s44320-026-00231-8 (PMC13639010; doi:10.1038/s44320-026-00231-8)
Supplement: Supplementary file 2 [file 44320_2026_231_MOESM1_ESM.pdf]

Appendix - Integrated metabolomics data analysis to generate mechanistic hypotheses with MetaProViz

Table of Contents

Appendix Figure S1. MetaProViz example data and folder structure overview. 1

Appendix Figures

A

ccRCC studies	Sample type	Omics type
Cellines	Intracellular	Metabolomics
Cellines	Culture media	Metabolomics
Patients	Intracellular	Metabolomics
Patients	Intracellular	RNAseq & Proteomics

B

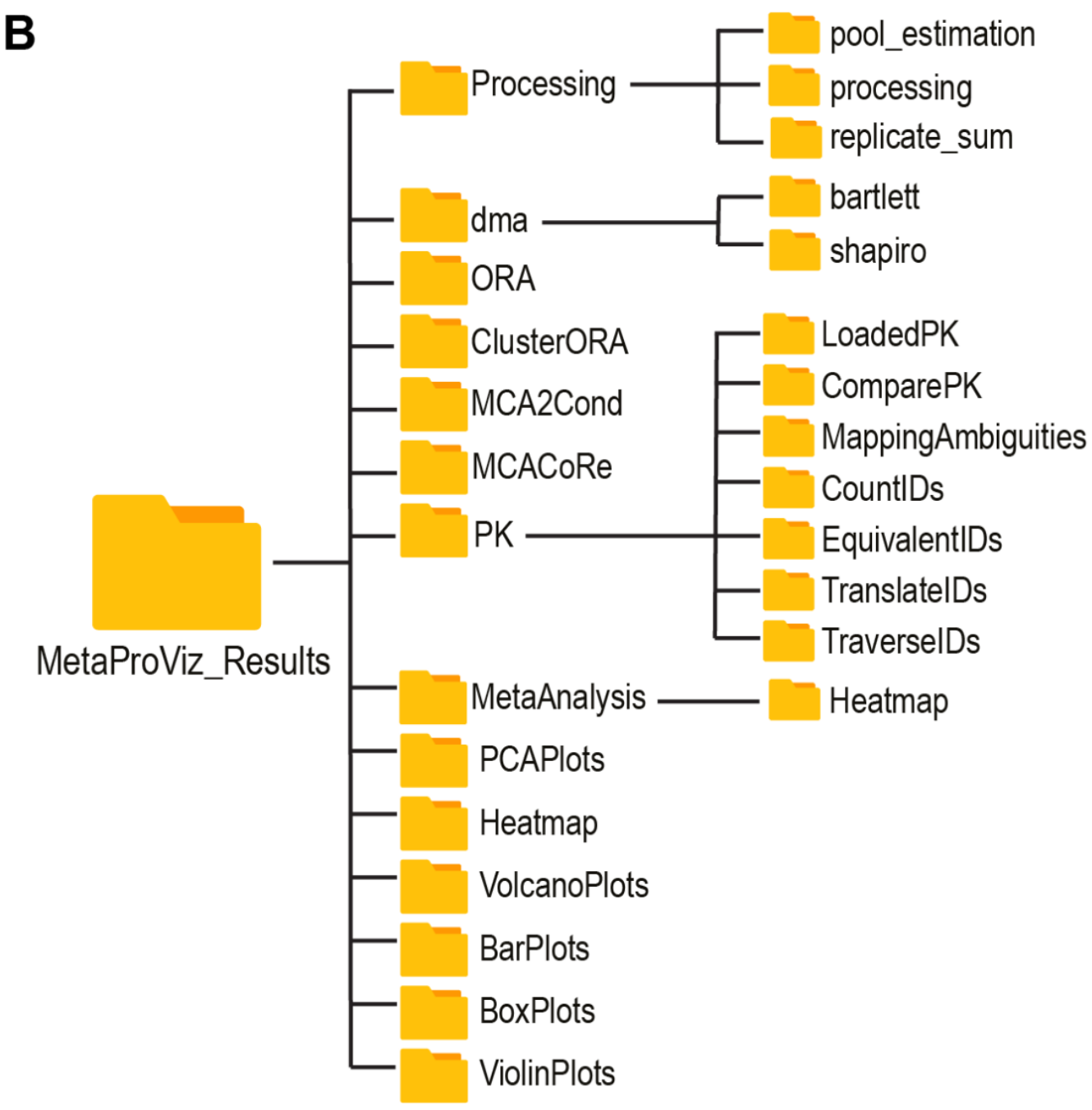

Appendix Figure S1: MetaProViz example data and folder structure overview.
a) Overview of available ccRCC example data in MetaProViz. **b)** Output folder structure where the result tables and figures are automatically saved for MetaProViz analysis.
