# Supplementary material for: Integrated metabolomics data analysis to generate mechanistic hypotheses with MetaProViz
Source: Mol Syst Biol. 2026 Aug 17;22(10):1605–25. doi: 10.1038/s44320-026-00231-8 (PMC13639010; doi:10.1038/s44320-026-00231-8)
Supplement: Supplementary file 3 [file 44320_2026_231_MOESM2_ESM.pdf]

molecular systems biology

Integrated metabolomics data analysis to generate mechanistic hypotheses with MetaProViz

Christina Schmidt, Jannik Franken, Dénes Türei, Dimitrios Prymidis, Macabe Daley, Christian Frezza, and Julio Saez-Rodriguez

Corresponding author(s): Julio Saez-Rodriguez (saezlab@ebi.ac.uk) , Christian Frezza (christian.frezza@uni-koeln.de)

Review Timeline:

Transfer from Review Commons:	10th Feb 26
Editorial Decision:	16th Feb 26
Revision Received:	16th Apr 26
Editorial Decision:	28th May 26
Revision Received:	10th Jun 26
Accepted:	24th Jun 26

Review COMMONS

Editor: Poonam Bheda

Transaction Report:

(Note: With the exception of the correction of typographical or spelling errors that could be a source of ambiguity, letters and reports are not edited. Depending on transfer agreements, referee reports obtained elsewhere may or may not be included in this compilation. Referee reports are anonymous unless the Referee chooses to sign their reports.)

Review #1

1. Evidence, reproducibility and clarity:

Evidence, reproducibility and clarity (Required)

Schmidt et al. present MetaProViz, a comprehensive and modular platform for metabolomics data analysis. The tool provides a full suite of processing capabilities spanning metabolite annotation, quality control, normalization, differential analysis, integration of prior knowledge, functional enrichment, and visualization. The authors also include example datasets, primarily from renal cancer studies, to demonstrate the functionality of the pipeline. The MetaProViz framework addresses several long-standing challenges in metabolomics data analysis, particularly issues of reproducibility, ambiguous metabolite annotation, and the integration of metabolite features with pathway knowledge. The platform is likely to be a valuable addition for the community, but the reviewer has some comments that need to be addressed prior to publication.

The section "Improving the connection between prior knowledge and metabolomics features" could benefit from additional clarification. It is not entirely clear to the reader what specific steps were taken beyond using RaMP-DB to translate metabolite identifiers. For example, how exactly were ambiguous mappings ("different scenarios") handled in practice, and to what extent does this process "fix" or merely flag inconsistencies? A more explicit description or example of how MetaProViz resolves these cases would help readers better understand the improvements claimed.

The introduction of MetSigDB is intriguing, but its construction and added value are not sufficiently described. It would be helpful to clarify what specific advantages MetSigDB provides over directly using existing pathway resources such as KEGG, Reactome, or WikiPathways. For example, how many features, interactions, or metabolite-set relationships are included, and in what way are these pathways improved or extended compared to those already available in public databases?

Figure 1D/1E: The reviewer appreciates the inclusion of the visualizations illustrating the different mapping scenarios, as these effectively convey the complexity of metabolite ID translation. However, it took some time to interpret what each scenario represented. It would be helpful to include brief annotations or explanatory text directly on the figures to clarify what each scenario depicts and how it relates to the underlying issue being addressed.

"By assigning other potential metabolite IDs and by translating between the present ID types, we not only increase the number of features within all ID types but also increase the feature space with HMDB and KEGG IDs (Fig. 2a, right, SFig. 2 and Supplementary Table 1)". The reviewer would appreciate additional clarification on how this was done. It is not clear what specific steps or criteria were used to assign additional metabolite IDs or to translate between identifier types. The reviewer also appreciates the inclusion of the UpSet plots. However, simply having the plots side-by-side makes it difficult to determine the specific differences. An alternative visualization, such as stacked bar plots, scatter plots summarizing the changes in feature counts, or other representation that more clearly highlights the deltas, might make these results easier to interpret.

MetaboAnalyst is mentioned several times in the manuscript. The reviewer is familiar with some of the limitations and practical challenges associated with using MetaboAnalyst and its R package. Given that MetaboAnalyst already offers some overlapping functionality with MetaProViz (and offers it in the form of an interactive website and a sometimes functional R package), a more explicit comparison between the two tools would help readers fully understand the unique advantages and improvements provided by MetaProViz.

Page 11: The authors state that they used limma for statistical testing, including for the analysis of exometabolomics data, where the values appear to represent log2-transformed distances or ratios rather than normally distributed intensities. Since limma assumes approximately normal residuals, please provide evidence or justification that this assumption holds for these data types. If the distributions deviate substantially from normality, a non-parametric alternative might be more appropriate.

Page 13: why were young and old defined this way? Authors should provide their reasoning and/or citations for this grouping.

Figure 4e: It may help with interpretation to have these Sankey-like graph edges be proportional to the number of metabolites.

Figure 4h: The values appear to be on an intensity scale (e.g., on the order of 3e10), yet some of them are negative, which would not be expected for raw or log-transformed mass spectrometry intensities. It is unclear whether these represent normalized abundance values, distances, or some other transformation. In addition, for the comparison of tumour versus normal tissue, it is not specified what statistical test was applied. Since mass spectrometry data are typically log2-transformed to approximate a log-normal distribution before performing t-tests or similar parametric methods, clarification is needed on how

these data were processed.

Figure 5: "Tukey's p.adj < 0.05" . Was this a Tukey's post-hoc test? This should be explicitly stated.

The potential for multi-omics is mentioned. Please clarify how generalizable this framework is. Can it readily accommodate transcriptomics, proteomics, or fluxomics data, or does it require custom logic or formatting for each new data type?

Please clarify if/how enrichment analyses account for varying set sizes and redundant metabolite memberships across pathways, which can bias over-representation analysis results.

2. Significance:

Significance (Required)

The MetaProViz framework addresses several long-standing challenges in metabolomics data analysis, particularly issues of reproducibility, ambiguous metabolite annotation, and the integration of metabolite features with pathway knowledge. The platform is likely to be a valuable addition for the community, but the reviewer has some comments that need to be addressed prior to publication.

Authors should be commended for the availability of data/code and detailed methods. Clarity is good. Authors have clearly spent a lot of time thinking about the challenges of metabolomics data analysis.

3. How much time do you estimate the authors will need to complete the suggested revisions:

Estimated time to Complete Revisions (Required)

(Decision Recommendation)

Between 1 and 3 months

4. *Review Commons* values the work of reviewers and encourages them to get credit for their work. Select 'Yes' below to register your reviewing activity at Web of Science Reviewer Recognition Service (formerly Publons); note that the content of your review will not be visible on Web of Science.

Web of Science Reviewer Recognition

Yes

Review #2

1. Evidence, reproducibility and clarity:

Evidence, reproducibility and clarity (Required)

Schmidt et al report the development of MetaProViz, an integrated R package to process, analyze and visualize metabolomics data, including integration with prior knowledge. The authors then go on to demonstrate utility by analyzing several metabolomes of cell lines, media and patient samples from kidney cancer. The manuscript provides a concise description of key challenges in metabolomics that the authors identify and address in their software. The examples are helpful and illustrative, although I should point out that I lack the expertise to evaluate the R package itself. I only have a few very minor comments.

Minor comments:

1. Figure 2D, E: While the schematics are fairly intuitive, a brief figure legend description of what the different scenarios etc. represent would make this easier to grasp.
2. Fig. 4: The authors briefly state that they integrate prior knowledge to identify the changes in methionine metabolism in kidney cancer, but it is not clear how exactly they contribute to this conclusion. It could be helpful to expand a bit on this to better illustrate how MetaProViz can be used to integrate prior knowledge into the analysis workflow.
3. Given the functional diversity among metabolites -central to diverse pathways, are key signaling molecules, restricted functions, co-variation within a pathway - I wonder how informative approaches such as PCA or enrichment analyses are for identifying metabolic drivers of a (patho)physiological state. To some extent, this can be addressed by integrating prior knowledge, and it would be helpful if the authors could comment on (and if applicable explain) whether/how this is integrated into MetaProViz.

2. Significance:

Significance (Required)

This is a very significant advance from one of the leading groups in the field that is likely to enhance metabolomics data analysis in the wider community.

3. How much time do you estimate the authors will need to complete the suggested revisions:

Estimated time to Complete Revisions (Required)

(Decision Recommendation)

Less than 1 month

4. *Review Commons* values the work of reviewers and encourages them to get credit for their work. Select 'Yes' below to register your reviewing activity at [Web of Science Reviewer Recognition Service] (formerly Publons); note that the content of your review will not be visible on Web of Science.

Web of Science Reviewer Recognition

No

Review #3

1. Evidence, reproducibility and clarity:

Evidence, reproducibility and clarity (Required)

This manuscript introduces an R package MetaProViz for metabolomics data analysis (post anotation), aiming to solve a poor-analysis-choices problem and enable more people to do the analysis. MetaProViz not only guides people to select the best statistical method, but also enables to solve previously unsolved problems: e.g. multiple and variable metabolite names in different databases and their connections to prior knowledge. They also created exometabolomics analysis and the needed steps to visualise intra-cell / media processes. The authors demonstrated their new package via kidney cancer (clear-cell renal cell carcinoma dataset, steping one step closer to improve biological interpretability of omics data analysis.

Major comments affecting conclusions: none.

Minor comments, important issues that could be addressed and possibly improve the clarity or generally presentation of the tool. Please see all below.

1. You start with separating and talking about metabolomics and lipidomics, but lipidomics

quickly dissapears (especially beyond abstract/intro) - no real need to discuss lipidomics.
2. You refer to the MetImp4 imputation web tool, but I cannot find an active website, manuscript, or R package for it, and the cited link does not load. This raises doubts about whether the tool is currently usable. Additionally, imputation choice should be guided by biological context and study design, not just by testing a few methods and selecting the one that performs best.
3. The authors address key metabolomics issues such as ambiguous metabolite names and isoforms, and their focus on resolving mapping ambiguities and translating between database identifiers is highly valuable. However, the larger challenge of de novo identification and the "dark matter" of unannotated metabolites remains unresolved (initiatives as MassIVE might help in the future https://massive.ucsd.edu/ProteoSAFe/), and readers may benefit from clearer acknowledgement that MetaProViz does not operate on raw spectral data. The introduction currently emphasizes annotation, but since MetaProViz requires already annotated metabolite tables (and then deals with all the messiness), this space might be better used to frame the interpretability and pathway-analysis challenges that the tool directly addresses.
4. I also really enjoyed you touching on the point of user-friendly but then inflexible and problem of reproducibility. We truly need well working packages for other bioinformaticians, rather than expecting wet-lab scientists to do all the analysis within the user interface.
5. It would be helpful to explain why the authors chose cancer/RCC samples for the demonstration. Was it because the dataset included both media and cell measurements? Does the tool perform best when multiple layers of information are available from the same experiment?
6. Figure 2B: The upset plots effectively show increased overlap after adaptation, but it would be easier to compare changes if the order of the intersection bars in the "adapted" plot matched the original. For example, while total intersections increased (251→285), the PubChem+KEGG overlap decreased (24→5), likely due to reallocation to the full intersection.
7. In your example of D-alanine and L-alanine - you mention how chirality is important biological feature, but up to this point it's not clear how do you do translation exactly and in which situations this would be treated just as "alanine" and when the more precise information would be retained? You mention RaMP-DB knowledge and one to X mappings as well as your general guidance in the "methods" part, but it would be useful to describe in this publication how you exactly tackled this problem in the ccRCC case.
8. In one to many mappings, it would be interesting to see quantification how frequently it was happening within a pathway or across pathways. I.e. Would going into pathway analysis "solve" the issue of "lost in translation" or not really?

9. QC: the coefficient of variation (CV) helps identify features with high variability and thus low detection accuracy. Here it's important to acknowledge that if the feature is very variable between groups it can be extremely important, but if the feature is very variable within the group - only then one would have low trust in the accuracy.
10. Missing value imputation - while missing not at random is a great way to deal with missingness, it would be great to have options for others (not just MNAR), as missingness is of a complex nature. If a pretty strong decision has been made, it would be good to support this by some supplementary data (i.e. how results change while applying various combinations of missingness and why choosing MNAR seems to be the most robust).
11. In the pre-processing and imputation stages - it would be interesting to see a summary table of how many features are left after each stage.
12. Is there a reason not to do UMAP or PSL-DA graphs for outlier detection? Doing more than PCA would help to have more confidence in removing or retaining outliers in the cases where biological relevance is borderline.
13. Metadata vs metabolite features - can this be used beyond metabolomics (i.e. proteomics, transcriptomics, etc)? It can be always very useful when there are many metadata features and it's hard to pre-select beforehand which ones are the most biologically relevant.
14. While authors discussed what KEGG pathways were significantly deregulated, it would be interesting to see all the pathways that were affected (e.g. aPEAR "bubble" graphs can show this (https://github.com/kerseviciute/aPEAR) , or something similar to NES scores). I appreciate the trickiness of it, but it would be quite interesting to see how authors e.g. Figure5e narrowed it down to the two pathways and how all the others looked like.
15. Could you comment on the runtime of the pipeline? In particular, do the additional translation steps and use of multiple databases substantially affect computational speed?
16. I clap to the authors for automated checks if selected methods are appropriate!
17. My suggestion would be to also look into power calculation or p-value histogram. In your example you saw some clear signal, but very frequently research studies are under-sampled and while effect can be clearly seen, there are just not enough samples to have statistically significant hits.
18. Overall functional parts are novel and next step in helping with data interpretability, but I still found it hard to read into functionally clear insights (re to pathways / functional groupings of metabolites) - especially as you have e.g. enzyme-metabolite databases etc. I think clarity there could be improved and would help to get your message more widely across.

2. Significance:

Significance (Required)

This is a great tool and I can't wait to use it on many upcoming metabolomics projects! Authors tackle multiple ongoing issues within the field: from poor selection of statistical methods (they provide guidance or have default safer options) to the messiness of data annotation between databases and improving data interpretability. The field is still evolving quickly, and it's impossible to solve all problems with one package; thus some limitations within the package could be seen as a bit rigid. Nonetheless, this fully steps toward filling an existing methodological gap. All bioinformaticians doing metabolomic analysis, or those learning how to do it, will greatly benefit from this knowledge.

I myself lead a team of 6 bioinformaticians, and we do analysis for researchers, clinicians, drug discovery, and various companies. We run internal metabolomics pipelines every day and fully sympathise with the problems addressed by the authors.

3. How much time do you estimate the authors will need to complete the suggested revisions:

Estimated time to Complete Revisions (Required)

(Decision Recommendation)

Less than 1 month

4. *Review Commons* values the work of reviewers and encourages them to get credit for their work. Select 'Yes' below to register your reviewing activity at Web of Science Reviewer Recognition Service (formerly Publons); note that the content of your review will not be visible on Web of Science.

Web of Science Reviewer Recognition

Yes

Revision Plan

Manuscript number: RC-2025-03233
Corresponding author(s): Prof. Dr. Christian Frezza and Prof. Dr. Ing. Julio Saez-Rodriguez

[The "revision plan" should delineate the revisions that authors intend to carry out in response to the points raised by the referees. It also provides the authors with the opportunity to explain their view of the paper and of the referee reports.

The document is important for the editors of affiliate journals when they make a first decision on the transferred manuscript. It will also be useful to readers of the reprint and help them to obtain a balanced view of the paper.

If you wish to submit a full revision, please use our "Full Revision" template. ***It is important to use the appropriate template to clearly inform the editors of your intentions.****]*

1. General Statements [optional]

This section is optional. Insert here any general statements you wish to make about the goal of the study or about the reviews.

We thank the editors and reviewers for their careful and constructive assessment of our manuscript. We appreciate the time invested by all three reviewers and found their comments highly valuable for improving the clarity, scope, and interpretability of MetaProViz. Below, we outline our overall revision strategy and summarize the key changes already implemented as well as those planned for the revised manuscript.

As part of the revision process, we have **already addressed the majority (>70%) of comments raised by all three reviewers**, with a particular focus on improving clarity, methodological transparency, and interpretability of the presented analyses (see section 3 below). These revisions include expanded figure legends, additional methodological detail, clearer justification of analytical choices, and improved descriptions of how prior knowledge is integrated throughout the workflow.

In the remaining revision steps, we plan to:

- **Extend the manuscript text** to more explicitly describe how metabolite identifier translation ambiguities are handled in practice, including concrete examples from the ccRCC case study.
- **Expand the description of MetSigDB**, including its construction, scope, and advantages relative to existing pathway resources, supported by new Extended Data tables.
- **Add new summary and quantification tables** (Extended Data) to improve transparency of preprocessing.
- **Enhance data visualization and interpretability**, including additional plots that highlight feature count deltas after identifier translation and quantification of one-to-many and many-to-one mappings.
- **Prepare comparison to Metaboanalyst** to highlight MetaProViz features that can add to Metaboanalyst.

In addition to manuscript-level changes, we have also:

- Performed a large scale, systematic refactoring across the package to improve code quality, robustness, and maintainability.
- We initiated and nearly completed the Bioconductor submission and review process, which not only resulted in numerous improvements and adoption of higher quality standards, but as MetaProViz will be part of Bioconductor, it ensures a long-term continuous quality control.

We believe these revisions will strengthen our **software manuscript** and will help us to provide a robust and practical tool to enhance biological interpretation of metabolomics data. Our responses to individual comments are below in **blue**.

Changes implemented to our manuscript text uses the following syntax:

- Strikethrough has been used to show where changes were made.
- Blue text indicates inclusion of new text.

In addition, we have added line numbers to the manuscript document, which we refer to in our responses where applicable.

2. Description of the planned revisions

Insert here a point-by-point reply that explains what revisions, additional experimentations and analyses are planned to address the points raised by the referees.

Reviewer #1

Comments:

(1)

The section "Improving the connection between prior knowledge and metabolomics features" could benefit from additional clarification. It is not entirely clear to the reader what specific steps were taken beyond using RaMP-DB to translate metabolite identifiers. For example, how exactly were ambiguous mappings ("different scenarios") handled in practice, and to what extent does this process "fix" or merely flag inconsistencies? A more explicit description or example of how MetaProViz resolves these cases would help readers better understand the improvements claimed.

We thank the reviewer for pointing this out and we agree that this section requires extension to ensure clarity.
Beyond using RaMP-DB, we are characterising the mapping ambiguity (one-to-none, one-to-many, many-to-one, many-to-many) within and across metabolite-sets (i.e. pathways) and return this information to the user together with the translated identifiers. This is important to understand potential inflation/deflation of metabolite-sets that occur due to the translation. Moreover, we also offer the manually curated amino-acid collection to ensure L-, D- and zwitterion without chirality IDs are assigned for aminoacids (**Fig. 2b**).
Ambiguous mappings are handled based on the measured data (**Fig. 2e**). Indeed, many translation cases that deflate (many-to-one mapping) or inflate (one-to-many mapping) the metabolite-sets are resolved when merging the prior knowledge with actual measured data (i.e. **Fig. 2e**, one-to-many in scenario 1, which becomes obsolete as only one/none of the many potential metabolite IDs is detected). By sorting each mapping into one of those scenarios, we only flag those cases. The reason for this decision has been that in many cases multiple decisions are valid (i.e. **Fig. 2e**, Scenario 5: Here the values of the two detected metabolites could be summed or the metabolite value with the larger Log2FC could be kept) and it should really be up to the user to make those dependent on their knowledge of the biological system and the analytical LC-MS method used.
Since these points have not been clear enough, we will add a more explicit description to the results section by showcasing more details on how we exactly tackled this problem in the ccRCC example data. This has also been suggested by **Reviewer 3** (**Minor Comment 7 and 8**), so feel free to also see the responses below.

(2)

The introduction of MetSigDB is intriguing, but its construction and added value are not sufficiently described. It would be helpful to clarify what specific advantages MetSigDB provides over directly using existing pathway resources such as KEGG, Reactome, or WikiPathways. For example, how many features, interactions, or metabolite-set relationships are included, and in what way are these pathways improved or extended compared to those already available in public databases?

We thank the reviewer for this valuable comment and we apologise that this was not described sufficiently. One of the major advantages is that all the resources are available in one place following the same table format without the need to visit the different original resources and perform data wrangling prior to enrichment analysis. In addition, where applicable, we have removed metabolites that are not detectable by LC-MS (i.e. ions, H_2O, CO_2) to circumvent pathway inflation with features that are never within the data and hence impacting the statistical testing in enrichment analysis workflows.

During the revision, we will compile an Extended Data Table listing all the resources present in MetSigDB, their number of features and interactions. We will also extend the methods section "Prior Knowledge access" about MetSigDB and how we removed metabolites.

(4)

"By assigning other potential metabolite IDs and by translating between the present ID types, we not only increase the number of features within all ID types but also increase the feature space with HMDB and KEGG IDs (Fig. 2a, right, SFig. 2 and Supplementary Table 1)". The reviewer would appreciate additional clarification on how this was done. It is not clear what specific steps or criteria were used to assign additional metabolite IDs or to translate between identifier types. The reviewer also appreciates the inclusion of the UpSet plots. However, simply having the plots side-by-side makes it difficult to determine the specific differences. An alternative visualization, such as stacked bar plots, scatter plots summarizing the changes in feature counts, or other representation that more clearly highlights the deltas, might make these results easier to interpret.

The main **Fig. 2a** shows the original (left) metabolite ID availability per detected metabolite feature in the ccRCC data and the adapted (right) metabolite IDs. The individual steps taken to extend the metabolite ID coverage of the measured features and obtain Fig 2a (right), are shown in **SFig. 2** for HMDB (**SFig. 2a**) and KEGG (**SFig. 2b**). We did not include the plots for the pubchem IDs as they follow the same principle. The individual steps we are showcasing with **SFig. 2** are (I) How many of the detected features (577) have a HMDB ID (341, red bar + grey bar), (II) How this distribution changed after equivalent amino-acid IDs are added, which does not change the number of features with an HMDB ID, but the number of features with a single HMDB ID, and (III) How this distribution changed after translating from the other available ID types (KEGG and PubChem) to HMDB IDs using RaMP-DBs knowledge, which leads to 430 detected features with one or multiple HMDB IDs. The exact numbers can be extracted from **Supplementary Table 1**, Sheet **"Feature metadata"**, where for example N-methylglutamate had no HMDB ID assigned in the original publication (see column HMDB_Original), yet by translating HMDB from KEGG

(hmdb_from_kegg) and PubChem (see column hmdb_from_pubchem) we obtain in both cases the same HMDB ID "HMDB0062660". In order to clarify this in the manuscript, we have extended the figure legend of **SFig. 2**: "***a-b)*** *Bargraphs showing the frequency at which a certain number of metabolite IDs per integrated peak are available as per ccRCC patients feature metadata provided in the original publication (left), after potential equivalent IDs for amino-acid and amnio-acid-related features were assigned (middle), which increases the number of features with multiple (middle: grey bars) and after IDs were translated from the other available ID types (right). ~~for~~* ***a)*** *Of 577 detected features, 341 had at least one HMDB ID~~s~~ assigned (left graph, red + grey bar) according to the original publication (left). Translating from KEGG-to-HMDB and from PubChem-to-HMDB increased the number of features with an HMDB ID from 341 to 430 (left). ~~and~~* ***b)*** *Of 577 detected features, 306 had at least one KEGG IDs assigned (left graph, red + grey bar) according to the original publication (left). Translating from HMDB-to-KEGG and from PubChem-to-KEGG did not increase the total number of features with an KEGG ID (left).*"

We like the suggestion of the reviewer to provide representations of the deltas and will add additional plots to **SFig. 2** as part of our planned revision.

(5)

MetaboAnalyst is mentioned several times in the manuscript. The reviewer is familiar with some of the limitations and practical challenges associated with using MetaboAnalyst and its R package. Given that MetaboAnalyst already offers some overlapping functionality with MetaProViz (and offers it in the form of an interactive website and a sometimes functional R package), a more explicit comparison between the two tools would help readers fully understand the unique advantages and improvements provided by MetaProViz.

This is a good point the reviewer raises. As part of the revisions, we plan to create a supplementary data table that includes both tools and their respective features. We will refer to this table within the manuscript text.

Reviewer #3

Minor Comments:

(7)

7- In your example of D-alanine and L-alanine - you mention how chirality is important biological feature, but up to this point it's not clear how do you do translation exactly and in which situations this would be treated just as "alanine" and when the more precise information would be retained? You mention RaMP-DB knowledge and one to X mappings as well as your general guidance in the "methods" part, but it would be useful to describe in this publication how you exactly tackled this problem in the ccRCC case.

We thank the reviewer for this suggestion. Since this is a complex problem, we will add a more explicit description to the results section by showcasing more details on how we exactly tackled this problem in the ccRCC example data.

In regards to D- and L-alanine, even though chirality is an important biological feature, in a standard experiment we can not distinguish if we detect the L- or D-aminoacid. This is why we try

to assign all possible IDs to increase the overlap with the prior knowledge. In **Fig. 2b** we showcase that this can potentially lead to multiple mappings of the same measured feature to multiple pathways. For example, if we measure alanine and assign the pubchem ID for L-Alanine, D-Alanine and Alanine and try to map to metabolite-sets that include both L-Alanine and D-Alanine. In turn this could fall into Scenario 6 (**Fig. 2e**), where across pathways there is a D-Alanine specific one (Pathway 1) and a L-Alanine specific one (Pathway 2). Now we can decide, if we want to allow both mapping (many-to-one) or if we decide to exclude D-Alanine because we know our biological system is human and should primarily have L-Alanine.

(8)

8- In one to many mappings, it would be interesting to see quantification how frequently it was happening within a pathway or across pathways. I.e. Would going into pathway analysis "solve" the issue of "lost in translation" or not really?

We have quantified the frequency for the example of translating the KEGG metabolite-set into HMDB IDs (**Fig. 2c**, left panel). Yet, we are not showcasing the quantification across the KEGG metabolite-sets with this plot. During the revision we will add the full results available to the **Extended Data Table 2**, which currently only includes the results displayed in **Fig.2c**.

(11)

11- In the pre-processing and imputation stages - it would be interesting to see a summary table of how many features are left after each stage.

This is a good suggestion and refers to the steps described in **Fig. 3a**. We will create an overview table for this, add it into the Extended Data Table and refer to it in the results section.

3. Description of the revisions that have already been incorporated in the transferred manuscript

Please insert a point-by-point reply describing the revisions that were <u>*already carried out and included*</u> *in the transferred manuscript. If no revisions have been carried out yet, please leave this section empty.*

Reviewer #1

Evidence, reproducibility and clarity

Authors should be commended for the availability of data/code and detailed methods. Clarity is good. Authors have clearly spent a lot of time thinking about the challenges of metabolomics data analysis.

Significance

Schmidt et al. present MetaProViz, a comprehensive and modular platform for metabolomics data analysis. The tool provides a full suite of processing capabilities spanning metabolite annotation, quality control, normalization, differential analysis, integration of prior knowledge, functional

enrichment, and visualization. The authors also include example datasets, primarily from renal cancer studies, to demonstrate the functionality of the pipeline. The MetaProViz framework addresses several long-standing challenges in metabolomics data analysis, particularly issues of reproducibility, ambiguous metabolite annotation, and the integration of metabolite features with pathway knowledge. The platform is likely to be a valuable addition for the community, but the reviewer has some comments that need to be addressed prior to publication.

We thank the reviewer for this positive feedback.

Comments:

(3)

Figure 1D/1E: The reviewer appreciates the inclusion of the visualizations illustrating the different mapping scenarios, as these effectively convey the complexity of metabolite ID translation. However, it took some time to interpret what each scenario represented. It would be helpful to include brief annotations or explanatory text directly on the figures to clarify what each scenario depicts and how it relates to the underlying issue being addressed.

We think the reviewer refers to Fig. 2D/E and we acknowledge that this is a complex problem we try to convey. We received a similar comment from Reviewer 2 (Minor Comment 1), who asked to extend the figure legend description of what the different scenarios display.

We have extended the figure legend and specifically explained each displayed case and its meaning (Line 222-242):

*"**d-e)** Schematics of possible mapping cases between metabolite IDs (= each circle corresponds to one ID) of a pathway-metabolite set (e.g. KEGG) to metabolites IDs of a different database (e.g. HMDB) with **(d)** showing many-to-many mappings that can occur within and across pathway-metabolite sets and **(e)** additionally showing the mapping to metabolite IDs that were assigned to the detected peaks within and across pathway-metabolite sets. **(d)** Translating the metabolite IDs of a pathway-metabolite set can lead to special cases such as many-to-one mappings (Pathway 1), where for example the original resource used the ID for L-Alanine (Pathway 1, green) and D-Alanine (Pathway 1, yellow) in the amino-acid pathway, whilst the translated resources only has an entry for Alanine zwitterion (Pathway 1, blue). Additionally, many-to-one mappings can also occur across pathways (Pathway 2-4), where this mapping is only detected when mappings are analysed taking all pathways into account. Both of these cases deflate the pathways, which can also happen for one-to-none mappings (Pathway 1, white). There are also cases that inflate the pathway such as one-to-many mappings (e.g. Pathway 2-4, orange mapping to pink and violet). **(e)** Showcasing the different scenarios when merging measured data (detected) based on the translated metabolites within pathways (scenario 1-5) and across pathways (scenario 6-8) highlighting problematic scenarios (4-7) that require further actions. Unproblematic scenarios (1-3 and 8) can include special cases between original and translated (i.e. one-to-many in scenario 1), which become obsolete as only one/none of the many potential metabolite IDs is detected. Yet, if multiple metabolites are detected action is required (scenario 5), which can include building the sum of the multiple detected features or only keeping the one with the highest Log2FC between two conditions. Other special cases between original and translated (i.e. many-to-one in scenario 4 and 6) also depend on what has been mapped to the measured features. If features have been measured in those scenarios, pathway deflation (i.e. only one original entry remains) or measured feature duplication (the same measurement is mapped to many features in the prior*

knowledge) are the possible results within and across pathways. Those scenarios should be addressed on a case-by-case basis as they also require biological information to be taken into account."

We have also rearranged the Scenarios in **Fig. 2e**. We hope that together with the extended figure legend this is now clear.

(6)

Page 11: The authors state that they used limma for statistical testing, including for the analysis of exometabolomics data, where the values appear to represent log2-transformed distances or ratios rather than normally distributed intensities. Since limma assumes approximately normal residuals, please provide evidence or justification that this assumption holds for these data types. If the distributions deviate substantially from normality, a non-parametric alternative might be more appropriate.

For exometabolomics data we use data normalised to media blank and growth factor (formula **(1)**). Limma is performed on those data, not on the log2-transformed distances. The Log2(Distance) is calculated separately to the statistical results using the normalised exometabolomics data. In addition, we always perform the Shapiro-Wilk test as part of MetaProViz differential analysis function on each metabolite to understand the distribution. In this particular case we have the following distributions:

Cell line	Metabolites normal distribution [%]	Metabolites not-normal distribution [%]
HK2	82.35	17.65
786-O	95.71	4.29
786-M1A	97.14	2.86
786-M2A	88.57	11.43
OSRC2	92.86	7.14
OSLM1B	85.71	14.29
RFX631	97.14	2.86

If a user would have distributions that deviate substantially from normality, non-parametric alternatives are also available in MetaProViz (see methods section for all options).

7)

Page 13: why were young and old defined this way? Authors should provide their reasoning and/or citations for this grouping.

We thank the reviewer for pointing this out. The explanation of our choices of the age groups is purely based on the literature:

First, ccRCC can be sporadic (>96%) or familial (<4%) by inheriting a mutation in VHL leading to ccRCC at an average age of 37 years (https://www.ncbi.nlm.nih.gov/pmc/articles/PMC3308682/pdf/nihms362390.pdf). This was also

observed in other cohorts, where of 1233 patients only 93 were under 40 years of age (%, whilst 1140 (%) were older than 40 years (https://www.europeanurology.com/article/S0302-2838(06)01316-9/fulltext). Second, given the high frequency of sporadic cases it is unsurprising that ccRCC incidences were found to peak in patients aged 60 to 79 years with more male than female incidences (https://journals.lww.com/md-journal/Fulltext/2019/08020/Frequency,_incidence_and_survival_outcomes_of.49.aspx). Third, it was shown that sex impacts on the renal cancer-specific mortality and is modified by age, which is a proxy for hormonal status with premenopausal period below 42 years and postmenopausal period above 58 years (https://www.ncbi.nlm.nih.gov/pmc/articles/PMC4361860/pdf/srep09160.pdf). Putting all of this information together, we decided on our age groups of young (<42 years), middle (42-58 years) and old (>58years) following the hormonal period in order to account for sex impact. Additionally, our young age group is representative of the age of familial ccRCC, whilst our old age group summarises the age group where incidences were found to peak.

To make this clear in the manuscript we have extended the method section of the manuscript (Line 547-548):

"For the patient's ccRCC data, we compared tumour versus normal of two patient subset, "young" (<42 years) and "old" (>58years)."

(8)

Figure 4e: It may help with interpretation to have these Sankey-like graph edges be proportional to the number of metabolites.

We thank the reviewer for this suggestion, which we also pondered. When we tested this visualisation, the plot became convoluted, hard to interpret and not all potential flows exist in the data. This is why we have opted to create an overview graph of each potential flow, with each edge representing a potentially existing flow. The number of times a flow exists is shown in Fig. 4f.

(9)

Figure 4h: The values appear to be on an intensity scale (e.g., on the order of 3e10), yet some of them are negative, which would not be expected for raw or log-transformed mass spectrometry intensities. It is unclear whether these represent normalized abundance values, distances, or some other transformation. In addition, for the comparison of tumour versus normal tissue, it is not specified what statistical test was applied. Since mass spectrometry data are typically log2-transformed to approximate a log-normal distribution before performing t-tests or similar parametric methods, clarification is needed on how these data were processed.

Thanks for pointing this out, it made us realize that we need to extend our figure legend for clarity for Fig. 4h (Line 343-345). In both cases we show normalized intensities following the workflow described in Fig. 3a. In case of the left graph labelled "CoRe", we are plotting an exometabolomics experiment, were additionally normalised using both media blanks (samples where no cells were

cultured in) and growth factor (accounts for cell growth during experiment) as growth rate (accounts for variations in cell proliferation) has not been available (see also formula (1) in methods section). A result has a negative value if the metabolite has been consumed from the media, or a positive value if the metabolite has been released from the cell into the culture media.

In addition, the reviewer refers to the comparison of tumour versus normal (**Fig. 4a** and **4d**) and the missing description of the chosen statistical test. We have added the details to the figure legend (Lines 334 and 345).

Adapted legend **Fig. 4**: "***a)*** *Differential metabolite analysis results for exometabolomics data comparing 786-O versus HK2 cells using Annova and false discovery rate (FDR) for p-value adjustment.* ***b)*** *Heatmap of mean consumption-release of the measured metabolites across cell lines.* ***c)*** *Heatmap of normalised ccRCC cell line exometabolomics data for the selected metabolites of amino acid metabolism for a sample subset.* ***d)*** *Differential metabolite analysis results for intracellular data comparing 786-O versus HK2 cells using Annova and false discovery rate (FDR) for p-value adjustment.* ***e)*** *Schematics of bioRCM process to integrate exometabolomics with intracellular metabolomics and* ***f)*** *number of metabolites by their combined change patterns in intracellular- and exometabolomics in 786-M1A versus HK2.* ***g)*** *Heatmap of the metabolite abundances in the "Both_DOWN (Released/Comsumed)" cluster.* ***h)*** *Bar graphs of normalised methionine intensity for exometabolomics (CoRe: negative value, if the metabolite has been consumed from the media, or a positive value, if the metabolite has been released from the cell into the culture media) and intracellular metabolomics (Intra).*"

(10)

Figure 5: "Tukey's p.adj < 0.05" . Was this a Tukey's post-hoc test? This should be explicitly stated.

We thank the reviewer for pointing this out. We have used the TukeyHSD (Tukey's Honestly Significant Difference) test in R on the Anova results. We have added more details into the figure legend (Line 384): "(Tukey's post-doc test after anova p.adj < 0.05)"

(11)

The potential for multi-omics is mentioned. Please clarify how generalizable this framework is. Can it readily accommodate transcriptomics, proteomics, or fluxomics data, or does it require custom logic or formatting for each new data type?

Thanks for raising this question. MetaProViz can readily accommodate transcriptomics and proteomics data for combined enrichment analysis using for example MetalinksDB metabolite-receptor pairs. Yet, MetaProViz does not support modelling fluxomics data into metabolic networks. We state in the discussion that this could be future development ("Beyond current capabilities, future developments could also incorporate mechanistic modeling to capture metabolic fluxes, subcellular compartmentalization, enzyme kinetics, regulatory feedback loops, and thermodynamic constraints to dissect metabolic response under perturbations."). To clarify

on the availability of multi-omics integration for combined enrichment analysis, we have added some more details into the discussion section.

Line 467-469: "*In addition, providing knowledge of receptor-, transporter- and enzyme-metabolite pairs, MetaProViz can readily accommodate transcriptomics and proteomics data for combined enrichment analysis.*"

(12)

Please clarify if/how enrichment analyses account for varying set sizes and redundant metabolite memberships across pathways, which can bias over-representation analysis results.

This is a very relevant point, which we have already been working on. Indeed, we agree that enrichment results from enrichment analyses can be biased due to varying set sizes and redundant metabolite memberships across pathways. MetaProViz explicitly accounts for varying set sizes when running over representation analysis (functions standard_ora()and cluster_ora()), which uses a model that computes the p-value under a hypergeometric distribution. Thereby, larger pathways are penalized unless the overlap is proportionally large, while smaller pathways can be significant with fewer overlaps. Hence, the test quantifies whether the observed overlap between the query set and a pathway is larger than would be expected under random sampling. In addition, we explicitly filter by gene-set size using min_gssize/max_gssize, which further controls for extreme small or large sets. So both the statistical test itself and the size filters incorporate gene-set size variation.

Regarding the redundant metabolite-set (i.e. pathways) memberships, we have now implemented a new function (cluster_pk()) to cluster metabolite-sets like pathways based on overlapping metabolites. Thereby we allow investigation of enrichment results in regard to redundancy and similarity. For given metabolite-sets, the function calculates pathway similarities via either overlap- or correlation-based metrics. After optional thresholding to remove weak similarities, we implemented three clustering algorithms (connected-components clustering, Louvain community detection and hierarchical clustering) to group similar pathways. We then visualize the clustering results as a network graph using the new function viz_graph based on igraph. We have added all information into our methods section "Metabolite-set clustering" (Lines 656-671). In addition, we have also added the results of the clustering into **Fig. 5f**.

New **Fig. 5f**:"***f)*** *Network graph of top enriched pathways (p.adjusted < 0.05) of tumour vs normal in young patients, generated using cluster_pk(). We used Jaccard similarity metrics, a similarity threshold of 0.6 and Louvain community detection for clustering. Node sizes represent the percentage of metabolites detected in each enriched pathway and pathway similarity defines edge weights.*"

F

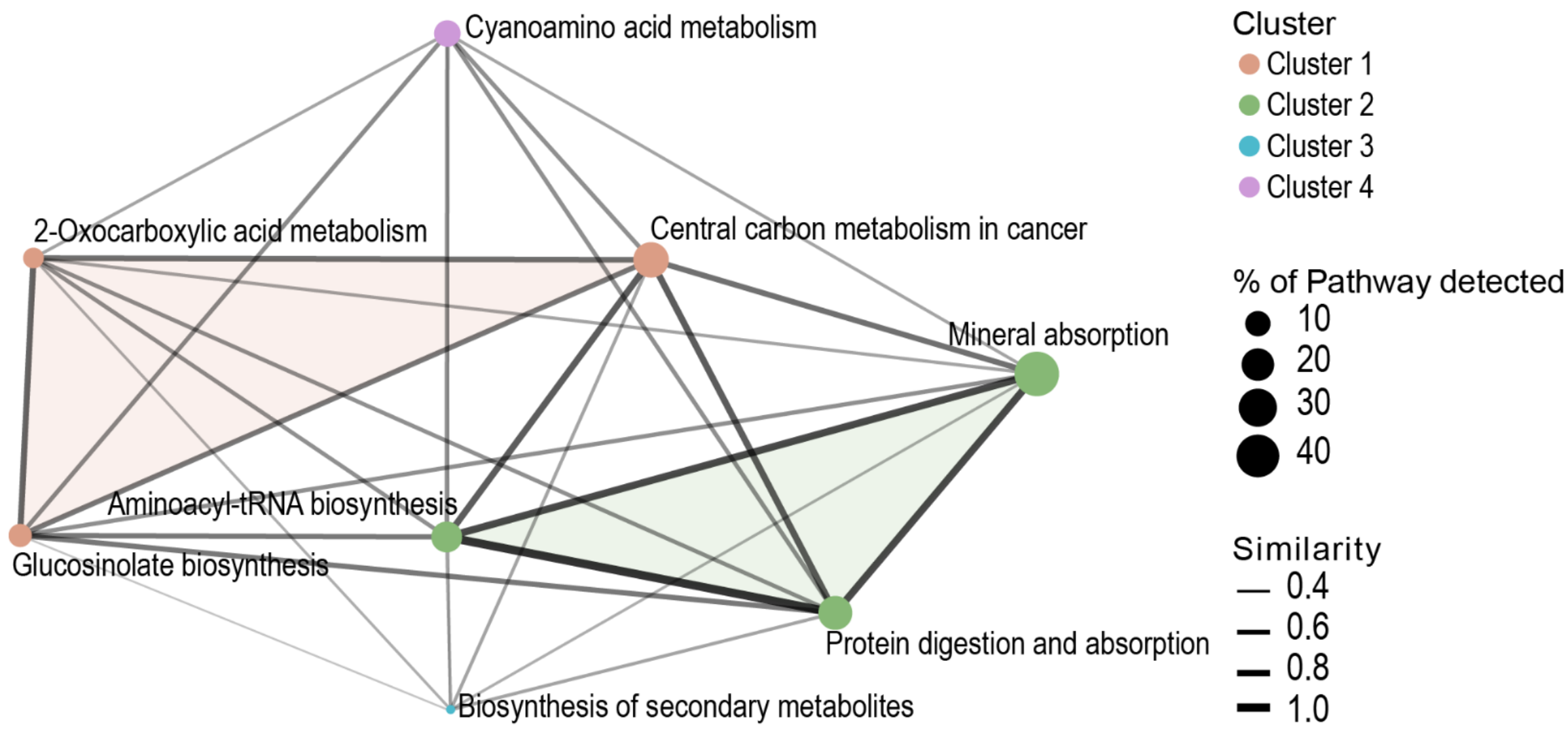

Reviewer #2

Evidence, reproducibility and clarity

Schmidt et al report the development of MetaProViz, an integrated R package to process, analyze and visualize metabolomics data, including integration with prior knowledge. The authors then go on to demonstrate utility by analyzing several metabolomes of cell lines, media and patient samples from kidney cancer. The manuscript provides a concise description of key challenges in metabolomics that the authors identify and address in their software. The examples are helpful and illustrative, although I should point out that I lack the expertise to evaluate the R package itself. I only have a few very minor comments.

Significance

This is a very significant advance from one of the leading groups in the field that is likely to enhance metabolomics data analysis in the wider community.

We thank the reviewer for this positive feedback on our package. We appreciate that there are no major comments from the reviewer.

Minor comments:

(1)

1. Figure 2D, E: While the schematics are fairly intuitive, a brief figure legend description of what the different scenarios etc. represent would make this easier to grasp.

We thank the reviewer for pointing this out and we acknowledge that this is a complex problem we try to convey. We received a similar comment from Reviewer 1 (Comment 3), so please see the extensive response there. In brief, we have extended the figure legend and specifically explained each displayed case and its meaning (Line 222-242) and extended the Figure itself by adding additional categories to **Fig. 2e**.

Extended legend **Fig.2 d-e**: "***d-e)*** *Schematics of possible mapping cases between metabolite IDs (= each circle corresponds to one ID) of a pathway-metabolite set (e.g. KEGG) to metabolites IDs of a different database (e.g. HMDB) with* ***(d)*** *showing many-to-many mappings that can occur within and across pathway-metabolite sets and* ***(e)*** *additionally showing the mapping to metabolite IDs that were assigned to the detected peaks within and across pathway-metabolite sets.* ***(d)*** *Translating the metabolite IDs of a pathway-metabolite set can lead to special cases such as many-to-one mappings (Pathway 1), where for example the original resource used the ID for L-Alanine (Pathway 1, green) and D-Alanine (Pathway 1, yellow) in the amino-acid pathway, whilst the translated resources only has an entry for Alanine zwitterion (Pathway 1, blue). Additionally, many-to-one mappings can also occur across pathways (Pathway 2-4), where this mapping is only detected when mappings are analysed taking all pathways into account. Both of these cases deflate the pathways, which can also happen for one-to-none mappings (Pathway 1, white). There are also cases that inflate the pathway such as one-to-many mappings (e.g. Pathway 2-4, orange mapping to pink and violet).* ***(e)*** *Showcasing the different scenarios when merging measured data (detected) based on the translated metabolites within pathways (scenario 1-5) and across pathways (scenario 6-8) highlighting problematic scenarios (4-7) that require further actions. Unproblematic scenarios (1-3 and 8) can include special cases between original and translated (i.e. one-to-many in scenario 1), which become obsolete as only one/none of the many potential metabolite IDs is detected. Yet, if multiple metabolites are detected action is required (scenario 5), which can include building the sum of the multiple detected features or only keeping the one with the highest Log2FC between two conditions. Other special cases between original and translated (i.e. many-to-one in scenario 4 and 6) also depend on what has been mapped to the measured features. If features have been measured in those scenarios, pathway deflation (i.e. only one original entry remains) or measured feature duplication (the same measurement is mapped to many features in the prior knowledge) are the possible results within and across pathways. Those scenarios should be addressed on a case-by-case basis as they also require biological information to be taken into account.*"

(2)

2. Fig. 4: The authors briefly state that they integrate prior knowledge to identify the changes in methionine metabolism in kidney cancer, but it is not clear how exactly they contribute to this conclusion. It could be helpful to expand a bit on this to better illustrate how MetaProViz can be used to integrate prior knowledge into the analysis workflow.

We think the reviewer refers to this section in the text (Line 363-370):

*"Next, we focused on the cluster "Both_DOWN (Released-Consumed)" and found that several amino acids are consumed by the ccRCC cell line 786-M1A but released by healthy HK2 cells. At the same time, intracellular levels are significantly lower than in HK2 (Log2FC = -0.9, p.adj = 4.4e-5) (**Fig. 4g**). To explore the role of these metabolites in signaling, we queried the prior knowledge resource MetalinksDB, which includes metabolite-receptor, metabolite-transporter and metabolite-enzyme relationships, for their known upstream and downstream protein interactors for the measured metabolites (**Supplementary Table 5**). This approach is especially valuable for exometabolomics, as it allows us to generate hypotheses about cell–cell communication. Notably, we identified links involving methionine (**Fig. 4h**), enzymes such as BHMT, and transporters such as SLC43A2 that were previously shown to be important in ccRCC[25,42] (**Supplementary Table 5**)."*

We have now extended this part to clearly state that here MetalinkDB is the prior knowledge resource we used to identify the links for methionine (Line 363-364). In addition we have extended our summary statement to ensure clarity for the reader that we combine the biological clustering,

which revealed the amino acid changes, with prior knowledge for the mechanistic insight (Line 380-381):

"*In summary, calculating consumption-release and combining it with intracellular metabolomics via biological regulated clustering reveals metabolites of interest. Further combining these results with prior knowledge using the MetaproViz toolkit facilitates biological interpretation of the data.*"

(3)

3. Given the functional diversity among metabolites -central to diverse pathways, are key signaling molecules, restricted functions, co-variation within a pathway - I wonder how informative approaches such as PCA or enrichment analyses are for identifying metabolic drivers of a (patho)physiological state. To some extent, this can be addressed by integrating prior knowledge, and it would be helpful if the authors could comment on (and if applicable explain) whether/how this is integrated into MetaProViz.

The reviewer is correct in stating the functional diversity of metabolites, which is also why prior knowledge is needed to add mechanistic interpretation to the finding from the metadata analysis (as we showcased by focusing on the separation of age (**Fig. 5c-d**)). We think that approaches such as PCA or enrichment can be helpful, even if admittedly limited. For example, in the metadata analysis presented in **Fig. 5b** and the subsequent enrichment analysis presented in **Fig. 5**, we used PCA to extract the eigenvector and the loading, which act as weights indicating the contribution of each original metabolite to that specific principal components separation. Hence, the eigenvector of PCA shows the metabolite drivers of the separation. This does not necessarily mean that those metabolites are drivers of a (patho)physiological state - the (patho)physiological state can equally be the reason for those metabolites driving the separation on the Eigenvectors. Thus, the metadata analysis presented in **Fig. 5b** enables us to extract the metadata variables (patho)physiological states separated on a PC with the explained variance. This can also lead to co-variation, when multiple (patho)physiological states are separated on the same PC, as the reviewer correctly points out. Regarding the enrichment analysis, we provide different types of prior knowledge for classical mapping, but also the prior knowledge we used to create the biological regulated clustering, which together help to identify key metabolic groups as we can first cluster the metabolites and afterwards perform functional enrichment. Yet, this does not account for the technical issues of enrichment analysis. In this context multi-omics integration building metabolic-centric networks could further elucidate the diversity of metabolic pathways and connection to signalling and co-variation, yet this is not the scope of MetaProViz. To sum up, we are aware of the limitations of this analysis and the constraints on the downstream interpretation.

To capture the functional diversity amongst metabolites, which leads to metabolites being present in multiple pathways of metabolite-pathways sets, we have implemented a new function to cluster metabolite-sets like pathways based on overlapping metabolites and visualize redundant metabolite-set (i.e. pathways) memberships (**Fig.5f**). For more details also see our response to Reviewer 1, Comment 12. We hope this will circumvent miss- and over-interpretation of the enrichment results.

In addition, we have extended the text to include the analysis pitfalls explicitly (Line 416-419): "*Another variable explaining the same amount of variance in PC1 is the tumour stage, which could point to adjacent normal tissue metabolic rewiring that happens in relation to stage and showcases that biological data harbour co-variations, which can not be disentangled by this method.*"

Reviewer #3

Evidence, reproducibility and clarity

This manuscript introduces an R package MetaProViz for metabolomics data analysis (post anotation), aiming to solve a poor-analysis-choices problem and enable more people to do the analysis. MetaProViz not only guides people to select the best statistical method, but also enables to solve previously unsolved problems: e.g. multiple and variable metabolite names in different databases and their connections to prior knowledge. They also created exometabolomics analysis and the needed steps to visualise intra-cell / media processes. The authors demonstrated their new package via kidney cancer (clear-cell renal cell carcinoma dataset, steping one step closer to improve biological interpretability of omics data analysis.

Significance

This is a great tool and I can't wait to use it on many upcoming metabolomics projects! Authors tackle multiple ongoing issues within the field: from poor selection of statistical methods (they provide guidance or have default safer options) to the messiness of data annotation between databases and improving data interpretability. The field is still evolving quickly, and it's impossible to solve all problems with one package; thus some limitations within the package could be seen as a bit rigid. Nonetheless, this fully steps toward filling an existing methodological gap. All bioinformaticians doing metabolomic analysis, or those learning how to do it, will greatly benefit from this knowledge.

I myself lead a team of 6 bioinformaticians, and we do analysis for researchers, clinicians, drug discovery, and various companies. We run internal metabolomics pipelines every day and fully sympathise with the problems addressed by the authors.

Major comments affecting conclusions

none.

We thank the reviewer for this positive feedback on evidence, reproducibility and clarity as well as significance of our work given the reviewers experience with metabolomics data analysis mentioned. We appreciate that there are no major comments from the reviewer.

Minor comments

Minor comments, important issues that could be addressed and possibly improve the clarity or generally presentation of the tool. Please see all below.

(1)

1- You start with separating and talking about metabolomics and lipidomics, but lipidomics quickly dissapears (especially beyond abstract/intro) - no real need to discuss lipidomics.

Thanks, that's a good note and we have removed it from the abstract and introduction.

(2)

2- You refer to the MetImp4 imputation web tool, but I cannot find an active website, manuscript, or R package for it, and the cited link does not load. This raises doubts about whether the tool is currently usable. Additionally, imputation choice should be guided by biological context and study design, not just by testing a few methods and selecting the one that performs best.

We fully agree with the reviewer on imputation handling. The manuscript we cite from Wei et. al. (https://doi.org/10.1038/s41598-017-19120-0) compared a multitude of missing value imputation methods and made this comparison strategy available as a web-based tool not as any code-based package such as an R-package. Yet, the reviewer is right, the web-tool is no longer reachable. Hence, we have adapted the statement in our introduction (Line 61-62): "*Moreover, there are tools that focus on specific steps of the pre-processing of feature intensities, which encompasses feature selection, missing value imputation (MVI)*[9] *and data normalisation.* ~~*For example, MetImp4 is a web-tool that includes and compares multiple MVI methods*[9]*.*~~ "

(3)

3- The authors address key metabolomics issues such as ambiguous metabolite names and isoforms, and their focus on resolving mapping ambiguities and translating between database identifiers is highly valuable. However, the larger challenge of de novo identification and the "dark matter" of unannotated metabolites remains unresolved (initiatives as MassIVE might help in the future https://massive.ucsd.edu/ProteoSAFe/), and readers may benefit from clearer acknowledgement that MetaProViz does not operate on raw spectral data. The introduction currently emphasizes annotation, but since MetaProViz requires already annotated metabolite tables (and then deals with all the messiness), this space might be better used to frame the interpretability and pathway-analysis challenges that the tool directly addresses.

We appreciate the comment and have highlighted this in the abstract and introduction: "*MetaProViz operates on annotated intensity values…*" (Line 29 and 88).

Given the newest advancements in metabolite identification using AI-based methods, MetaProViz toolkit with a focus on connecting metabolite IDs to prior knowledge becomes increasingly valuable. We added this to our discussion (Line 484-488): "*Given the imminent shift in metabolite identification through AI-based approaches, including language model-guided*[48] *methods and self-supervised learning*[49]*, the growing number of identified metabolites will make the MetaProViz toolkit increasingly valuable for the community to gain functional insights.*"

In regards to the introduction, where we mention some tools for peak annotation: The reason why we have this paragraph where peak annotation are named is that we wanted to set the basis by (I) listing the different steps of metabolomics data analysis and (II) pointing to well-known tools of those steps. We also have a dedicated paragraph for pathway-analysis challenges.

(4)

4- I also really enjoyed you touching on the point of user-friendly but then inflexible and problem of reproducibility. We truly need well working packages for other bioinformaticians, rather than expecting wet-lab scientists to do all the analysis within the user interface.

We thank the reviewer for this positive feedback.

(5)

5- It would be helpful to explain why the authors chose cancer/RCC samples for the demonstration. Was it because the dataset included both media and cell measurements? Does the tool perform best when multiple layers of information are available from the same experiment?

We specifically chose the ccRCC cell line data as example since, for a multitude of cell lines, both media (exometabolomics) and intracellular metabolomics had been performed. The combination of both data types is only used in the biological regulated clustering (Fig. 5e-g), all other analyses do not require additional data modalities. We have not specifically tested how performance differs for this particular case as it would require multiple paired data (exometabolomics and intracellular metabolomics) taken at the same time and at different times.

(6)

6- Figure 2B: The upset plots effectively show increased overlap after adaptation, but it would be easier to compare changes if the order of the intersection bars in the "adapted" plot matched the original. For example, while total intersections increased (251→285), the PubChem+KEGG overlap decreased (24→5), likely due to reallocation to the full intersection.

Thanks for raising this point. We initially had ordered the bars based on their intersection size, but we agree with the reviewers that for our point it makes sense to fix the order in the adapted plot to match the order of the original plot. We have done this (**Fig 2a**) and also extended the figure legend text of **SFig. 2**, which shows the individually performed adaptations summarized in **Fig 2a**.

(9)

9- QC: the coefficient of variation (CV) helps identify features with high variability and thus low detection accuracy. Here it's important to acknowledge that if the feature is very variable between groups it can be extremely important, but if the feature is very variable within the group - only then one would have low trust in the accuracy.

Yes, we totally agree with the reviewer on this. For this reason, we have applied CV only in instances where this is not leading to any condition-driven CV differences, but is truly feature-focused: (1) Function `pool_estimation` performs CV on the pool samples only, which are a homogeneous mixture of all samples, and hence can be used to assess feature variability. (2) Function `processing` performs CV on exometabolomics media samples (=blanks), which are also not impacted by different conditions.

(10)

10- Missing value imputation - while missing not at random is a great way to deal with missingness, it would be great to have options for others (not just MNAR), as missingness is of a complex nature. If a pretty strong decision has been made, it would be good to support this by some supplementary data (i.e. how results change while applying various combinations of missingness and why choosing MNAR seems to be the most robust).

We have decided to only offer support for MNAR, since we would recommend MVI only if there is a biological basis for it.

As mentioned in the response to your minor comment 2, Wei et. al. (https://doi.org/10.1038/s41598-017-19120-0) compared a multitude of missing value imputation methods. They compared six imputation methods (i.e., QRILC, Half-minimum, Zero, RF, kNN, SVD) for MNAR and systematically measured the performance of those imputation methods. They showed that QRILC and Half-Minimum produced much smaller SOR values, showing consistent good performances on data with different numbers of missing variables. This was the reason for us to only provide Half-minimum.

(12)

12- Is there a reason not to do UMAP or PSL-DA graphs for outlier detection? Doing more than PCA would help to have more confidence in removing or retaining outliers in the cases where biological relevance is borderline.

The reason we decided to use PCA was the standardly used combination with the Hotelling T2 outlier testing. Since PCA is a linear dimensionality reduction technique that preserves the overall variance in the data and has a clear mathematical foundation linked to the covariance structure, it specifically fits the required assumptions of the Hotelling T2 outlier testing. Indeed, Hotelling T2 relies on the properties of the covariance matrix and the assumption of a multivariate Gaussian distribution. UMAP is a non-linear dimensionality reduction technique, which prioritizes preserving local and global structures in a way that often results in good clustering visualization, but it distorts distances between clusters and does not have the same rigorous statistical underpinnings as PCA. In terms of PLS-DA, which focuses on maximizing the covariance between variables and the class labels, even though not commonly done, one could use the optimal latent variables for discrimination and apply Hotelling's T^2 to those latent variables. Yet, PLS-DA is supervised and actively tries to separate data points in the latent space, which can be misleading for outlier detection where methods like PCA that are unbiased, unsupervised and preserve global variance are advantageous.

---

(13)

13- Metadata vs metabolite features - can this be used beyond metabolomics (i.e. proteomics, transcriptomics, etc)? It can be always very useful when there are many metadata features and it's hard to pre-select beforehand which ones are the most biologically relevant.

Yes, definitely. In fact, we have used the metadata analysis strategy also with proteomics data and it will work equally with any omics data type.

(14)

14- While authors discussed what KEGG pathways were significantly deregulated, it would be interesting to see all the pathways that were affected (e.g. aPEAR "bubble" graphs can show this (https://github.com/kerseviciute/aPEAR) , or something similar to NES scores). I appreciate the trickiness of it, but it would be quite interesting to see how authors e.g. Figure5e narrowed it down to the two pathways and how all the others looked like.

We thank the reviewer for the suggestion of the aPEAR graphs. Following this suggestion, we have implemented a new function to enable clustering of the pathways based on overlapping metabolites (cluster_pk()). For more details regarding the method see also our response to Reviewer 1 (Comment 12) and our extended method section "Metabolite-set clustering" (Lines 656-671). We visualize the clustering results as a network graph, which we also included into **Fig. 5f**.

The complete result of the KEGG enrichment can be found in Extended Data Table 1, Sheet 13 (Pathway enrichment analysis using KEGG on Young patient subset). The pathways are ranked by p.adjusted value and also include a score (FoldEnrichment) from the fishers exact test (similar to NES scores in GSEA). Here one can find a total of seven pathways with a p.adjusted value < 0.05, of which we showcase two in **Fig. 5e**

For **Fig. 5e** we narrowed down to these two pathways based on the previous findings of dysregulated dipeptides (**Fig. 5d**), as we searched for a potential explanation of this observation.

(15)

15- Could you comment on the runtime of the pipeline? In particular, do the additional translation steps and use of multiple databases substantially affect computational speed?

Downloading and parsing databases takes significant time, especially large ones like RaMP or HMDB might take minutes on a standard laptop. Our local cache speeds up the process by eliminating the need for repeated downloads. In the future, database access will be even faster: according to our plans, all prior knowledge will be accessible in an already parsed format by our own API (omnipathdb.org). The ambiguity analysis, which is a complex data transformation pipeline, and plotting by ggplot2, another key component of MetaProViz, are the slowest parts, especially when performing analysis for the first time when no cache can be used. This means there are a few slow operations which complete in maximum a few dozens of seconds. However, the implementation and speed of these solutions doesn't fall behind what we commonly find in bioinformatics packages, and most importantly, the speed of MetaProViz doesn't pose an obstacle or difficulty regarding an efficient use of it in analysis pipelines.

(16)

16- I clap to the authors for automated checks if selected methods are appropriate!

Thank you, this is something we think is important to ensure correct analysis and circumvent misinterpretation.

(17)

17- My suggestion would be to also look into power calculation or p-value histogram. In your example you saw some clear signal, but very frequently research studies are under-sampled and while effect can be clearly seen, there are just not enough samples to have statistically significant hits.

We fully agree that power calculations are very important. Yet, this should ideally happen prior to the user's experiment. MetaProViz analysis starts at a later time-point and power calculations should have been done before. In regards to p-value histogram, we have implemented a similar measure, namely a density plot, which is plotted as a quality control measure within MetaProViz differential analysis function. The density plot is a smoothed version of a histogram that represents the distribution as a continuous probability density function and can be used to assess whether the p-values follow a uniform distribution.

(18)

18- Overall functional parts are novel and next step in helping with data interpretability, but I still found it hard to read into functionally clear insights (re to pathways / functional groupings of metabolites) - especially as you have e.g. enzyme-metabolite databases etc. I think clarity there could be improved and would help to get your message more widely across.

Regarding the clarity to the pathway enrichment and their functional insights, we have extended the Figure legends of Fig. 4 and 5, clearly state that for the functional interpretation MetalinkDB is the prior knowledge resource we used to identify the links for methionine (Line 367-368), and we have extended our summary statement to highlight that we combine the biological clustering with prior knowledge for the mechanistic insight (Line 380-381).

4. Description of analyses that authors prefer not to carry out

Please include a point-by-point response explaining why some of the requested data or additional analyses might not be necessary or cannot be provided within the scope of a revision. This can be due to time or resource limitations or in case of disagreement about the necessity of such additional data given the scope of the study. Please leave empty if not applicable.

None. We plan to address all points.

5. References

16th Feb 2026

Manuscript Number: MSB-2026-13622-T
Title: Integrated metabolomics data analysis to generate mechanistic hypotheses with MetaProViz

Dear Prof Saez-Rodriguez,

Thank you again for submitting your work to Molecular Systems Biology. We have now discussed your manuscript, the reviews from Review Commons, and your revision plan to address the remaining reviewer comments. We are happy to let you know that with the addition of the proposed revisions, we would send your paper back to the original reviewers who reviewed your paper at Review Commons for re-review at Molecular Systems Biology. Please let me know in case you would like to discuss in further detail, I would be happy to schedule a call.

When submitting your revised manuscript, please carefully review the instructions that follow below. We perform an initial quality control of all revised manuscripts before re-review; failure to include requested items will delay the evaluation of your revision.

We require:
1) A .docx formatted version of the manuscript text (including legends for main figures, EV figures and tables). Please make sure that the changes are highlighted to be clearly visible. Alternatively you may choose to submit your manuscript as a LaTeX file.

2) Individual production quality figure files as .eps, .tif, .jpg (one file per figure). For guidance, download the 'Figure Guide PDF' (https://media.springernature.com/original/springer-cms/rest/v1/content/27825798/data/v1).

3) At EMBO Press we ask authors to provide source data for the main figures. Our source data coordinator will contact you to discuss which figure panels we would need source data for and will also provide you with helpful tips on how to upload and organize the files.

4) A .docx formatted letter INCLUDING the reviewers' reports and your detailed point-by-point responses to their comments. As part of the EMBO Press transparent editorial process, the point-by-point response is part of the Peer Review File (PRF), which will be published alongside your paper.

5) A complete author checklist, which you can download from our author guidelines (https://link.springer.com/journal/44320/submission-guidelines#cms). Please insert information in the checklist that is also reflected in the manuscript. The completed author checklist will also be part of the PRF.

6) Please note that all corresponding authors are required to supply an ORCID ID for their name upon submission of a revised manuscript.

7) It is mandatory to include a 'Data Availability' section after the Materials and Methods. Before submitting your revision, primary datasets produced in this study need to be deposited in an appropriate public database, and the accession numbers and database listed under 'Data Availability'. Please remember to provide a reviewer password if the datasets are not yet public (see https://link.springer.com/partners/embo-press/editorial-policies#Data%20availability%20statement).

In case you have no data that requires deposition in a public database, please state so in this section as follows: "This study includes no data deposited in external repositories". Note that the Data Availability Section is restricted to new primary data that are part of this study.

8) All Materials and Methods need to be described in the main text using our 'Structured Methods' format, which is required for all research articles. According to this format, the Methods section includes a Reagents and Tools Table (listing key reagents, experimental models, software and relevant equipment and including their sources and relevant identifiers) followed by a Methods and Protocols section describing the methods using a step-by-step protocol format. The aim is to facilitate adoption of the methodologies across labs. Please upload the Reagents and Tools table as a separate document when submitting your revised manuscript. More information on how to adhere to this format as well as a downloadable template (.docx) for the Reagents and Tools Table can be found in our author guidelines: https://link.springer.com/journal/44320/submission-guidelines#cms

9) For data quantification: please specify the name of the statistical test used to generate error bars and p-values, the number (n) of independent experiments (specify technical or biological replicates) underlying each data point and the test used to calculate p-values in each figure legend. The figure legends should contain a basic description of n, p-values and the test applied. Graphs must include a description of the bars and the error bars (s.d., s.e.m.). Please provide exact p-values (in either the figure or figure legend).

10) Our journal encourages inclusion of *data citations in the reference list* to directly cite datasets that were re-used and obtained from public databases. Data citations in the article text are distinct from normal bibliographical citations and should directly link to the database records from which the data can be accessed. In the main text, data citations are formatted as follows: "Data ref: Smith et al, 2001" or "Data ref: NCBI Sequence Read Archive PRJNA342805, 2017". In the Reference list, data citations must be labeled with "[DATASET]". A data reference must provide the database name, accession number/identifiers and a resolvable link to the landing page from which the data can be accessed at the end of the reference. Further instructions are available at .

11) We replaced Supplementary Information with Expanded View (EV) Figures and Tables that are collapsible/expandable online. EV Figures should be cited as 'Figure EV1, Figure EV2" etc... in the text and their respective legends should be included in the main text after the legends of regular figures.
- For the figures that you do NOT wish to display as Expanded View figures, they should be bundled together with their legends in a single PDF file called *Appendix*, which should start with a short Table of Content. Appendix figures should be referred to in the main text as: "Appendix Figure S1, Appendix Figure S2" etc.
- Additional Tables/Datasets should be labeled and referred to as Table EV1, Dataset EV1, etc. Legends should be provided in a separate tab in case of .xls files. Alternatively, the legend can be supplied as a separate text file (README) and zipped together with the Table/Dataset file.
See detailed instructions here:
https://link.springer.com/journal/44320/submission-guidelines#cms

12) Author contributions: CRediT has replaced the traditional author contributions section because it offers a systematic machine-readable author contributions format that allows for more effective research assessment. Please remove the Authors Contributions from the manuscript and use the free text boxes beneath each contributing author's name in our system to add specific details on the author's contribution. More information is available in our guide to authors.

13) Disclosure statement and competing interests: We updated our journal's competing interests policy in January 2022 and request authors to consider both actual and perceived competing interests. Please review the policy https://link.springer.com/partners/embo-press/editorial-policies#Competing%20interest%20disclosures and update your competing interests if necessary.

14) Every published paper now includes a 'Synopsis' to further enhance discoverability. Synopses are displayed on the journal webpage and are freely accessible to all readers. They include a short stand first (maximum of 300 characters, including space) as well as 2-5 one-sentences bullet points that summarizes the paper. Please write the bullet points to summarize the key NEW findings. They should be designed to be complementary to the abstract - i.e. not repeat the same text. We encourage inclusion of key acronyms and quantitative information (maximum of 30 words / bullet point). Please use the passive voice. Please attach these in a separate file or send them by email, we will incorporate them accordingly.

Please also suggest a striking image or visual abstract to illustrate your article as a PNG file 550 px wide x 300-600 px high.

Please note that these would be the final versions and changes during proofing are usually not allowed.

15) As part of the EMBO Publications transparent editorial process initiative (see our policy here: https://link.springer.com/partners/embo-press/editorial-policies#Peer%20review), Molecular Systems Biology will publish online a Peer Review File (PRF) to accompany accepted manuscripts.
In the event of acceptance, this file will be published in conjunction with your paper and will include the anonymous referee reports, your point-by-point response and all pertinent correspondence relating to the manuscript. Let us know whether you agree with the publication of the PRF and as here, if you want to remove or not any figures from it prior to publication.
Please note that the Author checklist will be published at the end of the PRF.

Molecular Systems Biology has a "scooping protection" policy, whereby similar findings that are published by others during review or revision are not a criterion for rejection. Should you decide to submit a revised version, I do ask that you get in touch after three months if you have not completed it, to update us on the status.

Yours sincerely,

Poonam Bheda, PhD
Scientific Editor
Molecular Systems Biology

***

Rev_Com_number: RC-2025-03233
New_manu_number: MSB-2026-13622-T
Corr_author: Saez-Rodriguez
Title: Integrated metabolomics data analysis to generate mechanistic hypotheses with MetaProViz

Revision

Changes implemented to our manuscript use the following syntax:

- ~~Strikethrough~~ has been used to show where text was removed and blue text indicates inclusion of new text.
- Addition of new figure panels is visible by figure legend, since the new legends are blue.

We have added line numbers to the manuscript document, which we refer to in our responses where applicable

Reviewer: plain text highlighted in grey

Authors: Blue text

Reviewer #1

Evidence, reproducibility and clarity

Authors should be commended for the availability of data/code and detailed methods. Clarity is good. Authors have clearly spent a lot of time thinking about the challenges of metabolomics data analysis.

Significance

Schmidt et al. present MetaProViz, a comprehensive and modular platform for metabolomics data analysis. The tool provides a full suite of processing capabilities spanning metabolite annotation, quality control, normalization, differential analysis, integration of prior knowledge, functional enrichment, and visualization. The authors also include example datasets, primarily from renal cancer studies, to demonstrate the functionality of the pipeline. The MetaProViz framework addresses several long-standing challenges in metabolomics data analysis, particularly issues of reproducibility, ambiguous metabolite annotation, and the integration of metabolite features with pathway knowledge. The platform is likely to be a valuable addition for the community, but the reviewer has some comments that need to be addressed prior to publication.

We thank the reviewer for this positive feedback.

Comments:

(1)

The section "Improving the connection between prior knowledge and metabolomics features" could benefit from additional clarification. It is not entirely clear to the reader what specific steps were taken beyond using RaMP-DB to translate metabolite identifiers. For example, how exactly were ambiguous mappings ("different scenarios") handled in practice, and to what extent does this process "fix" or merely flag inconsistencies? A more explicit description or example of how MetaProViz resolves these cases would help readers better understand the improvements claimed.

We thank the reviewer for pointing this out and we agree that this section requires extension to ensure clarity.

Beyond using RaMP-DB, we are characterising the mapping ambiguity (one-to-none, one-to-many, many-to-one, many-to-many) within and across metabolite-sets (i.e. pathways) and return this information to the user together with the translated identifiers. This is important to understand potential inflation/deflation of metabolite-sets that occur due to the translation. Moreover, we also offer the manually curated amino-acid collection to ensure L-, D- and zwitterion without chirality IDs are assigned for aminoacids (**Figure EV 1a**, previously **Figure 2b**). Ambiguous mappings are handled based on the measured data by comparing the data feature space and the pathway feature space (**Fig. 2e**). Indeed, many translation cases that deflate (many-to-one mapping) or inflate (one-to-many mapping) the metabolite-sets are resolved when merging the prior knowledge with actual measured data (i.e. **Fig. 2e**, one-to-many in scenario 1, which becomes obsolete as only one/none of the many potential metabolite IDs is detected). By sorting each mapping into one of those scenarios, we only flag those cases. The reason for this decision has been that in many cases multiple decisions are valid (i.e. **Fig. 2e**, Scenario 5: Here the values of the two detected metabolites could be summed or the metabolite value with the larger Log2FC could be kept) and it should really be up to the user to make those dependent on their knowledge of the biological system and the analytical LC-MS method used.
Since these points have not been clear enough, we have now added a workflow figure, extended the results text and figure legends. Together, these additions showcase more details on how we exactly tackled this problem in the ccRCC example data. This has also been suggested by **Reviewer 3** (**Minor Comment 7 and 8**), so feel free to also see the responses below.
The extensive text changes in the results section can be found in line 204 following.

Workflow of **Fig. 2a**:

A

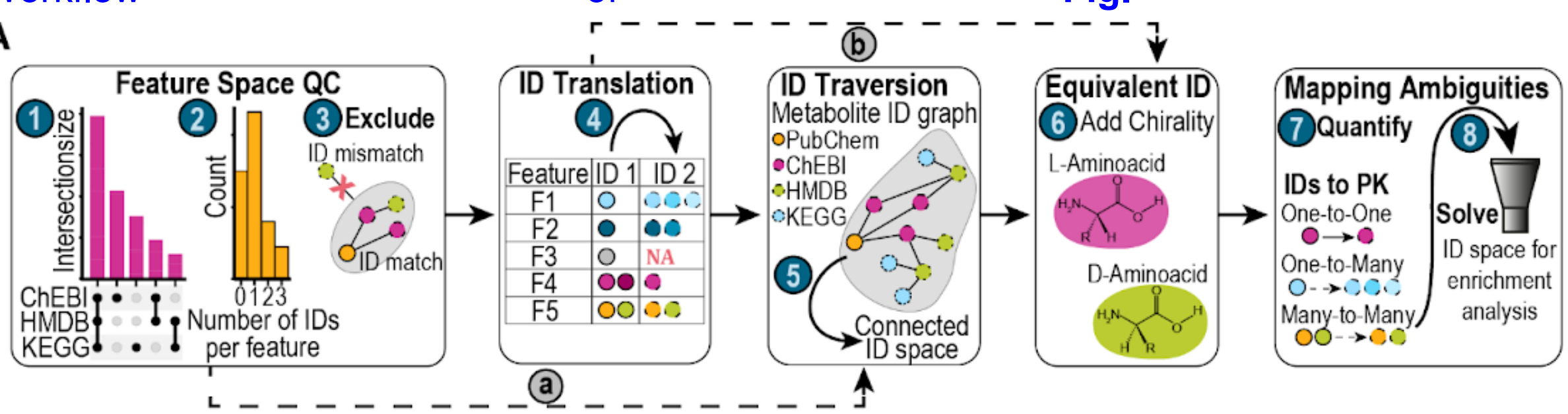

***a)** Overview of the feature ID workflow starting from annotated features with metabolite IDs and is divided into 8 steps: features space quality control (QC) based on (1) metabolite ID intersection analysis (2) quantification of number of IDs per feature and (3) ID mismatch identification if more than one ID type per feature is available, which are solved manually; (4) ID translation from one ID type into another ID type; (5) ID traversion using an ID graph, which adds new ID types and adds IDs to all present ID types; (6) addition of equivalent ID types based on chirality; (7) quantification and (8) manual solving of mapping ambiguities. Often either ID translation or ID traversion are performed and hence one of the two is skipped (route (a) and (b)).*

(2)

The introduction of MetSigDB is intriguing, but its construction and added value are not sufficiently described. It would be helpful to clarify what specific advantages MetSigDB provides over directly using existing pathway resources such as KEGG, Reactome, or WikiPathways. For example, how

many features, interactions, or metabolite-set relationships are included, and in what way are these pathways improved or extended compared to those already available in public databases?

We thank the reviewer for this valuable comment and we apologise that this was not described sufficiently. One of the major advantages is that all the resources are available in one place following the same table format without the need to visit the different original resources and perform data wrangling prior to enrichment analysis. In addition, where applicable, we have removed metabolites that are not detectable by LC-MS (i.e. ions, H2O, CO2) to circumvent pathway inflation with features that are never within the data and hence impacting the statistical testing in enrichment analysis workflows. We have now created a dedicated schematics for it (**Fig. 1b**):

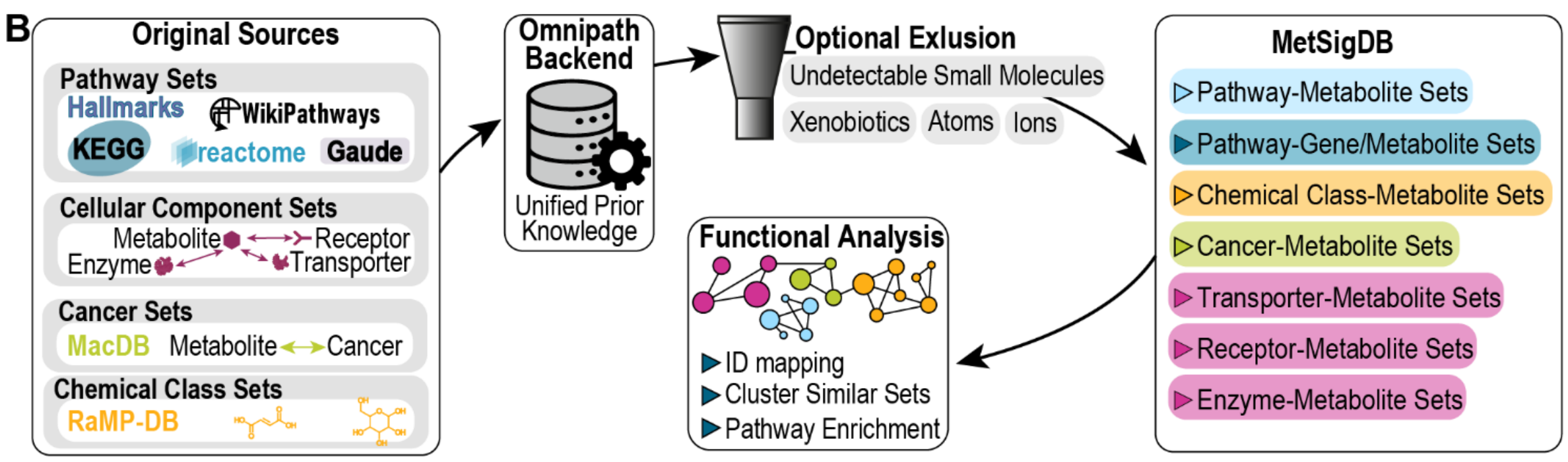

b) Overview of MetSigDB including original sources unified in omnipath, groups of metabolites for exclusion and the final MetSigDB metabolite sets that can be used for functional enrichment.

In addition, we have also compiled an Extended Data Table (**Table EV1**) listing all the resources present in MetSigDB, their number of features and interactions. We have also visualized part of this in **Fig 1c-d**:

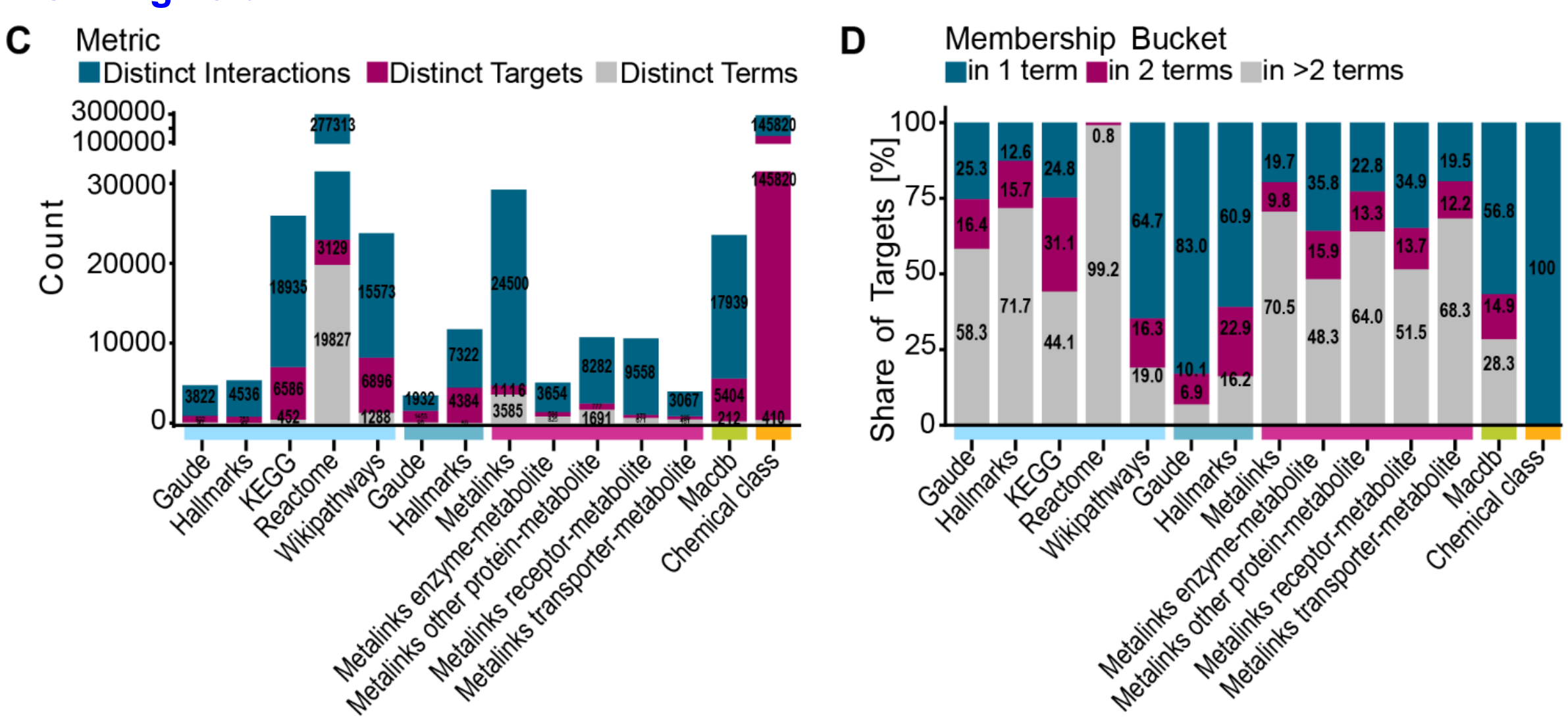

c-d) Metabolite sets are displayed on the x-axis including: pathway-metabolite sets (light blue), pathway-gene/metabolite sets (dark blue), metabolite-protein sets for transporter-, receptor- and enzyme-metabolite interactions (pink), cancer-metabolite set (green) and chemical class-metabolite set (yellow). Prior to plotting metabolites from the exclusion list were removed. **c)** Shows the number of distinct interactions,

targets and terms for each metabolite set and **d)** shows the percentages of shared targets between sets within a metabolite set (e.g. shared metabolites between two pathways in KEGG).

(3)

Figure 1D/1E: The reviewer appreciates the inclusion of the visualizations illustrating the different mapping scenarios, as these effectively convey the complexity of metabolite ID translation. However, it took some time to interpret what each scenario represented. It would be helpful to include brief annotations or explanatory text directly on the figures to clarify what each scenario depicts and how it relates to the underlying issue being addressed.

We think the reviewer refers to Fig. 2D/E and we acknowledge that this is a complex problem we try to convey. We received a similar comment from Reviewer 2 (Minor Comment 1), who asked to extend the figure legend description of what the different scenarios display.

We have extended the figure legend and results section to specifically explain each displayed case and its meaning.
Figure legend (Line 304-317):

*"**d-e)** Schematics of possible mapping cases between metabolite IDs (= each circle corresponds to one ID) of a pathway-metabolite set (e.g. KEGG) to metabolites IDs of a different database (e.g. HMDB) with **(d)** showing many-to-many mappings that can occur within and across pathway-metabolite sets and **(e)** additionally showing the mapping to metabolite IDs that were assigned to the detected peaks within and across pathway-metabolite sets. Different scenarios within pathways (scenario 1-5) and across pathways (scenario 6-8) are displayed, highlighting problematic scenarios (4-7) that require further actions. Problematic scenarios should be addressed on a case-by-case basis as they also require biological information to be taken into account."*

Results section (Line 256-277):

"*This mapping ambiguity can lead to problems when set enrichment analysis is performed since it can inflate or deflate the pathway-metabolite sets. Here we can have cases where both, the prior knowledge metabolite-sets and the measured data, or one of these two have multiple metabolite IDs per feature by applying ID translation/traversion. For all those cases~~Therefore~~, MetaProViz maps the detected metabolites to the pathway-metabolite sets and flags ambiguous or problematic scenarios to enable manual review. In detail, addition of metabolite IDs to a metabolite-set by translation or traversion can lead to special cases such as many-to-one mappings (**Fig.2d**, Pathway 1), where for example the original resource used the ID for L-Alanine (Pathway 1, green) and D-Alanine (Pathway 1, yellow) in the amino-acid pathway, whilst the translated resources only has an entry for Alanine zwitterion (Pathway 1, blue). Additionally, many-to-one mappings can also occur across pathways (**Fig.2d**, Pathway 2-4), where this mapping is only detected when mappings are analysed taking all pathways into account. Both of these cases deflate the pathways, which can also happen for one-to-none mappings (**Fig.2d,** Pathway 1, white). There are also cases that inflate the pathway such as one-to-many mappings (**Fig.2d,** e.g. Pathway 2-4, orange mapping to pink and violet). It is important to~~This~~ ensure~~s~~ that the number of features assigned to each pathway matches the original composition defined in the prior knowledge resource (**Fig. 2e**). In detail, there are scenarios that will require a reduction in features per pathway (**Fig. 2e**, Scenario 4) or the merge of two detected features by sum or mean (**Fig. 2e**, Scenario 5). Additionally, there are scenarios that only become apparent across pathways (**Fig. 2e**, Scenario 6-8) where decisions on a case-by-case basis are required.*"

We have also rearranged the Scenarios in **Fig. 2e**. We hope that together with the extended figure legend this is now clear.

(4)

"By assigning other potential metabolite IDs and by translating between the present ID types, we not only increase the number of features within all ID types but also increase the feature space with HMDB and KEGG IDs (Fig. 2a, right, SFig. 2 and Supplementary Table 1)". The reviewer would appreciate additional clarification on how this was done. It is not clear what specific steps or criteria were used to assign additional metabolite IDs or to translate between identifier types. The reviewer also appreciates the inclusion of the UpSet plots. However, simply having the plots side-by-side makes it difficult to determine the specific differences. An alternative visualization, such as stacked bar plots, scatter plots summarizing the changes in feature counts, or other representation that more clearly highlights the deltas, might make these results easier to interpret.

The main **Fig. 2b** (previously **Fig. 2a**) shows the original (left) metabolite ID availability per detected metabolite feature in the ccRCC data and the adapted (right) metabolite IDs. The individual steps taken to extend the metabolite ID coverage of the measured features and obtain **Fig 2b** (right), were shown in previous **SFig. 2** for HMDB and KEGG. To make the steps taken more clear, we have created a workflow overview (see responds to your comment 1 above). Moreover, we have now created plots showing how many metabolite IDs per feature were added in each step of the workflow based on our suggestion displaying the deltas:

Fig. 2c: Number of metabolite ID (x-axis) for HMDB, KEGG, PUBCHEM and CHEBI within the ccRCC patient feature space. "Original" is referring to the IDs provided by the original publication, "Cleaned" is after our ID mismatch check (= deltas are mostly negative), "Translated" are IDs that were added by translating from CAS numbers, "Traverse" are IDs added after using MetaProViz ID traversion method and "Equivalent ID" are IDs added based on stereoisomerism. The deltas describe the IDs gained through each step.

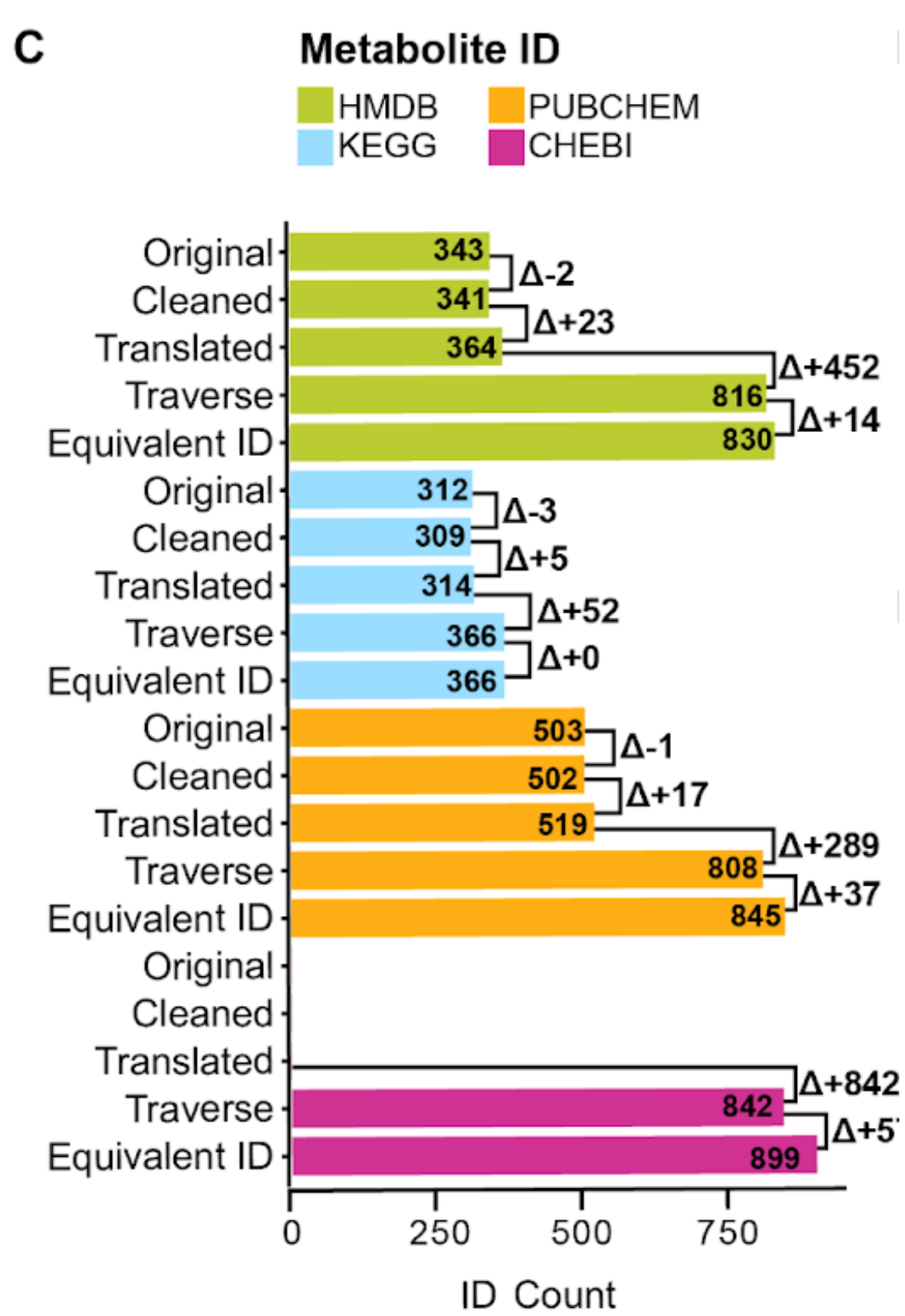

Figure EV1b (previously this was Supplementary Figure 2):

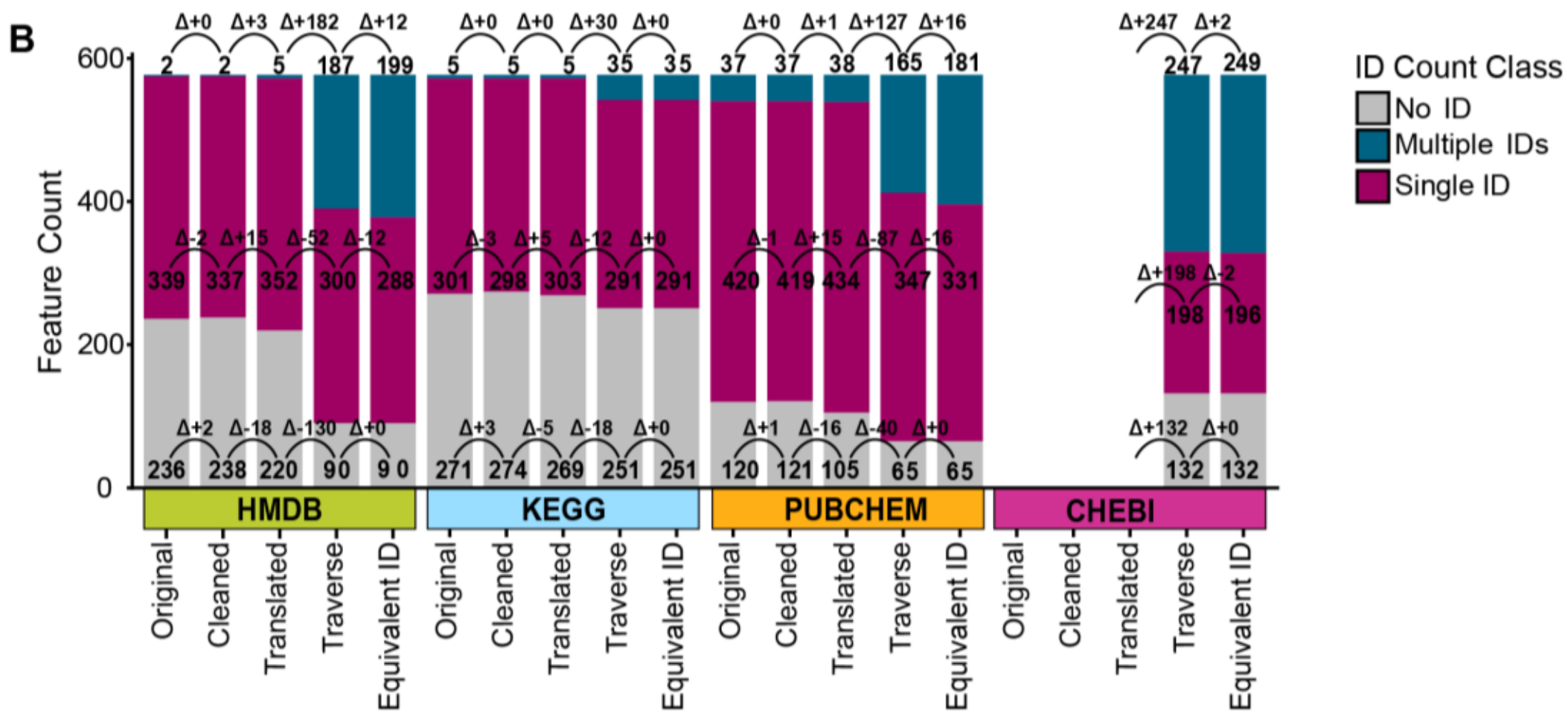

Stacked bargraphs ~~**a-b)** Bargraphs~~ showing the frequency at which a certain number of metabolite IDs per integrated peak are available (no, one or multiple) with the number of feature count (y-axis) for HMDB, KEGG, PUBCHEM and CHEBI within the ccRCC patient feature space. "Original" is referring to the IDs provided by the original publication, "Cleaned" is after our ID mismatch check (= deltas are mostly negative), "Translated" are IDs that were added by translating from CAS numbers, "Traverse" are IDs added after using MetaProViz ID traversion method and "Equivalent ID" are IDs added based on stereoisomerism. The deltas describe the IDs gained through each step and show that over the course of the ID workflow features without IDs (grey) are decreasing, whilst features with multiple IDs (blue) are increasing.

The exact numbers can be extracted from **Table EV2** (previously **Supplementary Table 1**), **Sheets 1, 3, 4, 5, 6** (**"**Raw_Feature_Metadata", "Feature_Metadata_Cleaned", "Feature_Metadata_Translated", "Feature_Metadata_Traverse", "Feature_Metadata_EquivalentID"), where we added detailed information on the features added in each step. The respective summary and deltas of feature counts in each workflow step can be found in Table EV2, sheets 8 and 9 ("ID_Summary", "ID_Summary_Deltas").

(5)

MetaboAnalyst is mentioned several times in the manuscript. The reviewer is familiar with some of the limitations and practical challenges associated with using MetaboAnalyst and its R package. Given that MetaboAnalyst already offers some overlapping functionality with MetaProViz (and offers it in the form of an interactive website and a sometimes functional R package), a more explicit comparison between the two tools would help readers fully understand the unique advantages and improvements provided by MetaProViz.

This is a good point the reviewer raises. MetaProViz and MetaboAnalyst implement comparable high-level workflows, both including preprocessing, differential analysis, functional analysis, and visualisation. Therefore, differences arise not at the module level, but in the depth, flexibility, and integration of specific functionalities. Noteworthy, the web-based version of MetaboAnalyst provides functionalities that are not fully replicated in MetaboAnalystR 4.0, such as the visualisations implemented using visual analytics tools based on JavaScript or Python.

Here it is important to point out that rather than replacing existing tools such as MetaboAnalyst, MetaProViz is designed to complement them, as it addresses limitations as for example metabolite identifier handling, prior knowledge integration handling, and exometabolomics analysis. Hence, for certain downstream analysis MetaboAnalyst offers in their webtool can be readily used with MetaProViz outputs.

Given the breadth and continuous development of MetaboAnalyst, this comparison focuses on selected features directly related to the methodological contributions of MetaProViz. The comparison we perform is based on documented functionality in publicly available documentation of MetaboAnalyst R 4.0 and focuses on features directly relevant to the methodological contributions of this study.

***Table EV1, sheet 6:* Unique conceptual contributions of MetaProViz.** *Comparison based on publicly available documentation and publications of MetaboAnalyst R. The table highlights selected features relevant to the methodological contributions of MetaProViz and is not intended to provide an exhaustive benchmark. MetaboAnalyst has many complementary features that are described elsewhere and are not part of MetaProiViz.*

**Category**	**Feature**	**MetaProViz**	**MetaboAnalyst R 4.0**
Workflow scope	End-to-end metabolomics workflow	✓ Modular workflow integrating processing, analysis, and interpretation	✓ Comprehensive workflows covering multiple analysis types with strong web-based focus
Data integration	Exometabolomics (consumption/release) analysis	✓ Dedicated workflows linking intra- and extracellular metabolite changes	◯ No dedicated workflows explicitly described in available documentation
Prior knowledge integration	General prior knowledge usage	✓ Integrated across multiple steps of analysis	✓ Primarily used for pathway/enrichment analysis
	Metabolite identifier (ID) translation	✓ Systematic translation across databases with annotation expansion	✓ ID mapping supported
	ID ambiguity quantification	✓ Explicit assessment and reporting of mapping ambiguities	◯ Not explicitly described
	Gene → metabolite set conversion	✓ Reaction-based conversion using metabolic networks (e.g. Recon3D/COSMOS)	◯ Not explicitly described
	Receptor/transporter integration	✓ Included via curated resources (e.g. MetalinksDB)	◯ Limited or not explicitly described

Functional analysis	Classical enrichment analysis	✓ Implemented	✓ Implemented
	Multi-condition / biologically informed clustering	✓ Implemented	◯ Limited or not explicitly described
	Integration with exometabolomics data	✓ Direct integration into functional analysis	◯ Not explicitly described
Usability & implementation	Reproducible workflows	✓ Scriptable, log-based workflows in R and Bioconductor	✓ Available as R package, Package dependencies need to be installed separately
	User guidance and automated checks	✓ Integrated messages, logging, and statistical guidance	◯ Limited or not explicitly described
	Interface focus	✓ Scriptable, workflow-oriented (Bioconductor ecosystem)	✓ Strong focus on web-based accessibility

(6)

Page 11: The authors state that they used limma for statistical testing, including for the analysis of exometabolomics data, where the values appear to represent log2-transformed distances or ratios rather than normally distributed intensities. Since limma assumes approximately normal residuals, please provide evidence or justification that this assumption holds for these data types. If the distributions deviate substantially from normality, a non-parametric alternative might be more appropriate.

For exometabolomics data we use data normalised to media blank and growth factor (formula **(1)**). Limma is performed on those data, not on the log2-transformed distances. The Log2(Distance) is calculated separately to the statistical results using the normalised exometabolomics data. In addition, we always perform the Shapiro-Wilk test as part of MetaProViz differential analysis function on each metabolite to understand the distribution. In this particular case we have the following distributions:

**Cell line**	**Metabolites normal distribution [%]**	**Metabolites not-normal distribution [%]**
HK2	82.35	17.65
786-O	95.71	4.29
786-M1A	97.14	2.86
786-M2A	88.57	11.43
OSRC2	92.86	7.14

OSLM1B	85.71	14.29
RFX631	97.14	2.86

If a user would have distributions that deviate substantially from normality, non-parametric alternatives are also available in MetaProViz (see methods section for all options).

7)

Page 13: why were young and old defined this way? Authors should provide their reasoning and/or citations for this grouping.

We thank the reviewer for pointing this out. The explanation of our choices of the age groups is purely based on the literature:

First, ccRCC can be sporadic (>96%) or familial (<4%) by inheriting a mutation in VHL leading to ccRCC at an average age of 37 years (https://www.ncbi.nlm.nih.gov/pmc/articles/PMC3308682/pdf/nihms362390.pdf). This was also observed in other cohorts, where of 1233 patients only 93 were under 40 years of age (%, whilst 1140 (%) were older than 40 years (https://www.europeanurology.com/article/S0302-2838(06)01316-9/fulltext). Second, given the high frequency of sporadic cases it is unsurprising that ccRCC incidences were found to peak in patients aged 60 to 79 years with more male than female incidences (https://journals.lww.com/md-journal/Fulltext/2019/08020/Frequency,_incidence_and_survival_outcomes_of.49.aspx). Third, it was shown that sex impacts on the renal cancer-specific mortality and is modified by age, which is a proxy for hormonal status with premenopausal period below 42 years and postmenopausal period above 58 years (https://www.ncbi.nlm.nih.gov/pmc/articles/PMC4361860/pdf/srep09160.pdf). Putting all of this information together, we decided on our age groups of young (<42 years), middle (42-58 years) and old (>58years) following the hormonal period in order to account for sex impact. Additionally, our young age group is representative of the age of familial ccRCC, whilst our old age group summarises the age group where incidences were found to peak.

To make this clear in the manuscript we have extended the method section of the manuscript (Line 691-692):

“For the patient's ccRCC data, we compared tumour versus normal of two patient subset, “young” (<42 years) and “old” (>58years).”

(8)

Figure 4e: It may help with interpretation to have these Sankey-like graph edges be proportional to the number of metabolites.

We thank the reviewer for this suggestion, which we also pondered. When we tested this visualisation, the plot became convoluted, hard to interpret and not all potential flows exist in the data. This is why we have opted to create an overview graph of each potential flow, with each edge representing a potentially existing flow. The number of times a flow exists is shown in **Fig. 4f**.

(9)

Figure 4h: The values appear to be on an intensity scale (e.g., on the order of 3e10), yet some of them are negative, which would not be expected for raw or log-transformed mass spectrometry intensities. It is unclear whether these represent normalized abundance values, distances, or some other transformation. In addition, for the comparison of tumour versus normal tissue, it is not specified what statistical test was applied. Since mass spectrometry data are typically log2-transformed to approximate a log-normal distribution before performing t-tests or similar parametric methods, clarification is needed on how these data were processed.

Thanks for pointing this out, it made us realize that we need to extend our figure legend for clarity for **Fig. 4h** (Line 459-463). In both cases we show normalized intensities following the workflow described in **Fig. 3a**. In case of the left graph labelled "CoRe", we are plotting an exometabolomics experiment, were additionally normalised using both media blanks (samples where no cells were cultured in) and growth factor (accounts for cell growth during experiment) as growth rate (accounts for variations in cell proliferation) has not been available (see also formula (1) in methods section). A result has a negative value if the metabolite has been consumed from the media, or a positive value if the metabolite has been released from the cell into the culture media.

In addition, the reviewer refers to the comparison of tumour versus normal (**Fig. 4a** and **4d**) and the missing description of the chosen statistical test. We have added the details to the figure legend (Lines 334 and 453).

Adapted legend **Fig. 4**: "***a)*** *Differential metabolite analysis results for exometabolomics data comparing 786-O versus HK2 cells using Annova and false discovery rate (FDR) for p-value adjustment (Shapiro-Wilk test: 96% (786-O) / 82% (HK2) of the metabolites follow a normal distribution).* ***b)*** *Heatmap of mean consumption-release of the measured metabolites across cell lines.* ***c)*** *Heatmap of the biological replicates of the normalised ccRCC cell line exometabolomics data for the selected metabolites of amino acid metabolism for a sample subset.* ***d)*** *Differential metabolite analysis results for intracellular data comparing 786-O versus HK2 cells using Annova and false discovery rate (FDR) for p-value adjustment (Shapiro-Wilk test: 95% (786-O) / 96% (HK2) of the metabolites follow a normal distribution).* ***e)*** *Schematics of bioRCM process to integrate exometabolomics with intracellular metabolomics and* ***f)*** *number of metabolites by their combined change patterns in intracellular- and exometabolomics in 786-M1A versus HK2.* ***g)*** *Heatmap of the biological replicates of the cell lines metabolite abundances in the "Both_DOWN (Released/Comsumed)" cluster.* ***h)*** *Bar graphs of normalised methionine intensity for exometabolomics (CoRe: negative value, if the metabolite has been consumed from the media, or a positive value, if the metabolite has been released from the cell into the culture media) and intracellular metabolomics (Intra). Dots represent biological replicates and p-adjusted values were calculated using Annova and false discovery rate (FDR).*"

(10)

Figure 5: "Tukey's p.adj < 0.05" . Was this a Tukey's post-hoc test? This should be explicitly stated.

We thank the reviewer for pointing this out. We have used the TukeyHSD (Tukey's Honestly Significant Difference) test in R on the Anova results. We have added more details into the figure legend (Line 517): "(Tukey's post-doc test after anova p.adj < 0.05)"

(11)

The potential for multi-omics is mentioned. Please clarify how generalizable this framework is. Can it readily accommodate transcriptomics, proteomics, or fluxomics data, or does it require custom logic or formatting for each new data type?

Thanks for raising this question. MetaProViz can readily accommodate transcriptomics and proteomics data for combined enrichment analysis using for example MetalinksDB metabolite-receptor pairs. Yet, MetaProViz does not support modelling fluxomics data into metabolic networks. We state in the discussion that this could be future development ("Beyond current capabilities, future developments could also incorporate mechanistic modeling to capture metabolic fluxes, subcellular compartmentalization, enzyme kinetics, regulatory feedback loops, and thermodynamic constraints to dissect metabolic response under perturbations."). To clarify on the availability of multi-omics integration for combined enrichment analysis, we have added some more details into the discussion section.

Line 594-596: "*In addition, providing knowledge of receptor-, transporter- and enzyme-metabolite pairs, MetaProViz can readily accommodate transcriptomics and proteomics data for combined enrichment analysis.*"

(12)

Please clarify if/how enrichment analyses account for varying set sizes and redundant metabolite memberships across pathways, which can bias over-representation analysis results.

This is a very relevant point, which we have already been working on. Indeed, we agree that enrichment results from enrichment analyses can be biased due to varying set sizes and redundant metabolite memberships across pathways. MetaProViz explicitly accounts for varying set sizes when running over representation analysis (functions `standard_ora()` and `cluster_ora()`), which uses a model that computes the p-value under a hypergeometric distribution. Thereby, larger pathways are penalized unless the overlap is proportionally large, while smaller pathways can be significant with fewer overlaps. Hence, the test quantifies whether the observed overlap between the query set and a pathway is larger than would be expected under random sampling. In addition, we explicitly filter by gene-set size using min_gssize/max_gssize, which further controls for extreme small or large sets. So both the statistical test itself and the size filters incorporate gene-set size variation.

Regarding the redundant metabolite-set (i.e. pathways) memberships, we have now implemented a new function (`cluster_pk()`) to cluster metabolite-sets like pathways based on overlapping metabolites. Thereby we allow investigation of enrichment results in regard to redundancy and similarity. For given metabolite-sets, the function calculates pathway similarities via either overlap- or correlation-based metrics. After optional thresholding to remove weak similarities, we implemented three clustering algorithms (connected-components clustering, Louvain community detection and hierarchical clustering) to group similar pathways. We then visualize the clustering results as a network graph using the new function viz_graph based on igraph. We have added all

information into our methods section “Metabolite-set clustering” (Lines 830-845). In addition, we have also added the results of the clustering into **Fig. 5f**.

New **Fig. 5f**:”***f)*** *Network graph of top enriched pathways (p.adjusted < 0.05) of tumour vs normal in young patients, generated using cluster_pk(). We used Jaccard similarity metrics, a similarity threshold of 0.6 and Louvain community detection for clustering. Node sizes represent the percentage of metabolites detected in each enriched pathway and pathway similarity defines edge weights.*”

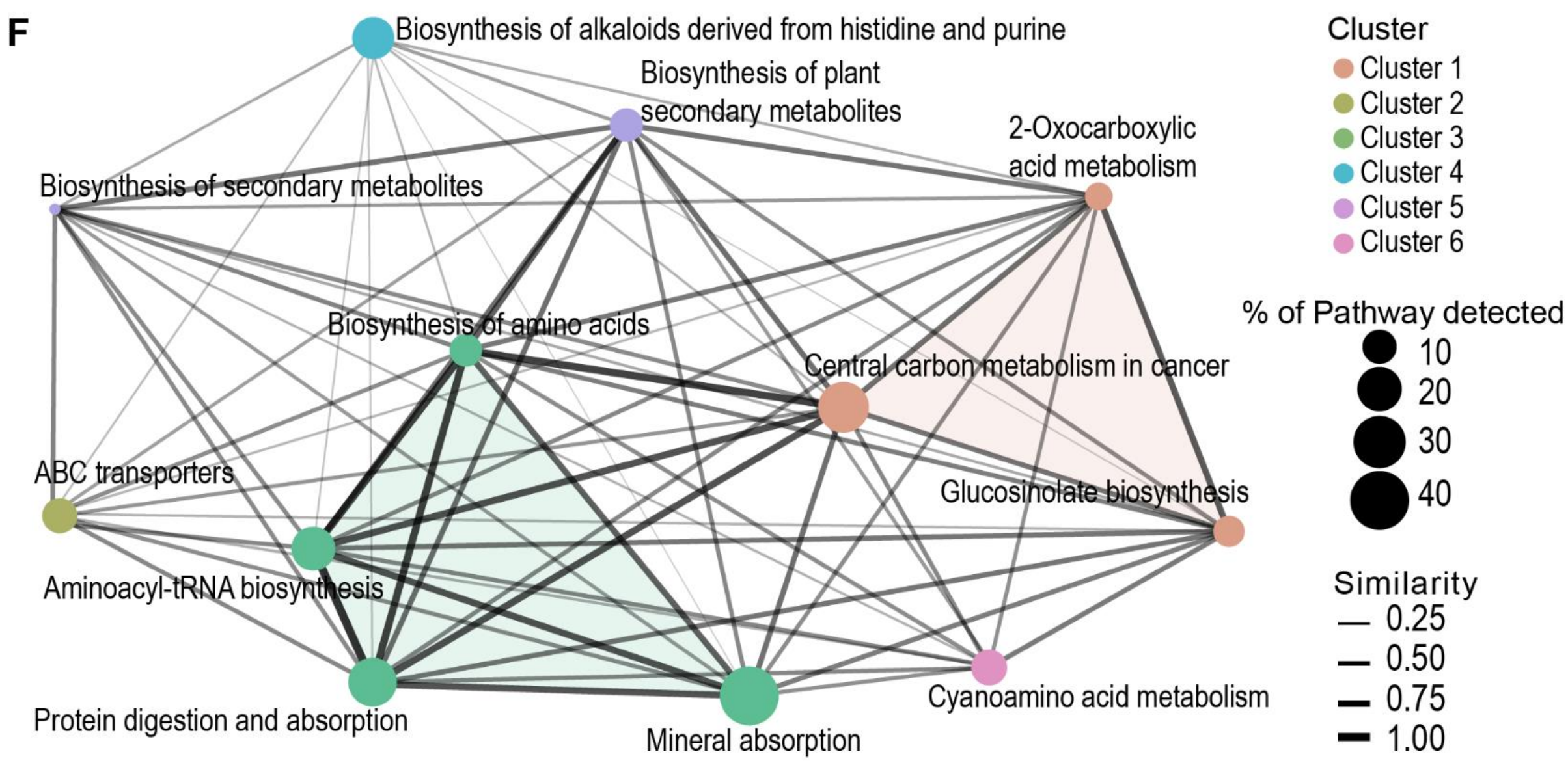

Reviewer #2

Evidence, reproducibility and clarity

Schmidt et al report the development of MetaProViz, an integrated R package to process, analyze and visualize metabolomics data, including integration with prior knowledge. The authors then go on to demonstrate utility by analyzing several metabolomes of cell lines, media and patient samples from kidney cancer. The manuscript provides a concise description of key challenges in metabolomics that the authors identify and address in their software. The examples are helpful and illustrative, although I should point out that I lack the expertise to evaluate the R package itself. I only have a few very minor comments.

Significance

This is a very significant advance from one of the leading groups in the field that is likely to enhance metabolomics data analysis in the wider community.

We thank the reviewer for this positive feedback on our package. We appreciate that there are no major comments from the reviewer.

Minor comments:

(1)

1. Figure 2D, E: While the schematics are fairly intuitive, a brief figure legend description of what the different scenarios etc. represent would make this easier to grasp.

We thank the reviewer for pointing this out and we acknowledge that this is a complex problem we try to convey. We have extended the figure legend and results section to specifically explain each displayed case and its meaning.
Figure legend (Line 304-317):

*"**d-e)** Schematics of possible mapping cases between metabolite IDs (= each circle corresponds to one ID) of a pathway-metabolite set (e.g. KEGG) to metabolites IDs of a different database (e.g. HMDB) with **(d)** showing many-to-many mappings that can occur within and across pathway-metabolite sets and **(e)** additionally showing the mapping to metabolite IDs that were assigned to the detected peaks within and across pathway-metabolite sets. Different scenarios within pathways (scenario 1-5) and across pathways (scenario 6-8) are displayed, highlighting problematic scenarios (4-7) that require further actions. Problematic scenarios should be addressed on a case-by-case basis as they also require biological information to be taken into account."*

Results section (Line 256-277):

"*This mapping ambiguity can lead to problems when set enrichment analysis is performed since it can inflate or deflate the pathway-metabolite sets. Here we can have cases where both, the prior knowledge metabolite-sets and the measured data, or one of these two have multiple metabolite IDs per feature by applying ID translation/traversion. For all those cases~~Therefore~~, MetaProViz maps the detected metabolites to the pathway-metabolite sets and flags ambiguous or problematic scenarios to enable manual review. In detail, addition of metabolite IDs to a metabolite-set by translation or traversion can lead to special cases such as many-to-one mappings (**Fig.2d**, Pathway 1), where for example the original resource used the ID for L-Alanine (Pathway 1, green) and D-Alanine (Pathway 1, yellow) in the amino-acid pathway, whilst the translated resources only has an entry for Alanine zwitterion (Pathway 1, blue). Additionally, many-to-one mappings can also occur across pathways (**Fig.2d**, Pathway 2-4), where this mapping is only detected when mappings are analysed taking all pathways into account. Both of these cases deflate the pathways, which can also happen for one-to-none mappings (**Fig.2d,** Pathway 1, white). There are also cases that inflate the pathway such as one-to-many mappings (**Fig.2d,** e.g. Pathway 2-4, orange mapping to pink and violet). It is important to~~This~~ ensure~~s~~ that the number of features assigned to each pathway matches the original composition defined in the prior knowledge resource (**Fig. 2e**). In detail, there are scenarios that will require a reduction in features per pathway (**Fig. 2e**, Scenario 4) or the merge of two detected features by sum or mean (**Fig. 2e**, Scenario 5). Additionally, there are scenarios that only become apparent across pathways (**Fig. 2e**, Scenario 6-8) where decisions on a case-by-case basis are required.*"

We have also rearranged the Scenarios in **Fig. 2e**. We hope that together with the extended figure legend this is now clear.

(2)

2. Fig. 4: The authors briefly state that they integrate prior knowledge to identify the changes in methionine metabolism in kidney cancer, but it is not clear how exactly they contribute to this

conclusion. It could be helpful to expand a bit on this to better illustrate how MetaProViz can be used to integrate prior knowledge into the analysis workflow.

We think the reviewer refers to this section in the text (Line 484-496):

*"Next, we focused on the cluster "Both_DOWN (Released-Consumed)" and found that several amino acids are consumed by the ccRCC cell line 786-M1A but released by healthy HK2 cells. At the same time, intracellular levels are significantly lower than in HK2 (Log2FC = -0.9, p.adj = 4.4e-5) (**Fig. 4g**). To explore the role of these metabolites in signaling, we queried the prior knowledge resource MetalinksDB, which includes metabolite-receptor, metabolite-transporter and metabolite-enzyme relationships, for their known upstream and downstream protein interactors for the measured metabolites (**Table EV3**). This approach is especially valuable for exometabolomics, as it allows us to generate hypotheses about cell–cell communication. Notably, we identified links involving methionine (**Fig. 4h**), enzymes such as BHMT, and transporters such as SLC43A2 that were previously shown to be important in ccRCC[25,42] (**Table EV3**)."*

We have now extended this part to clearly state that here MetalinkDB is the prior knowledge resource we used to identify the links for methionine (Line 484-496; blue text in paragraph above). In addition we have extended our summary statement to ensure clarity for the reader that we combine the biological clustering, which revealed the amino acid changes, with prior knowledge for the mechanistic insight (Line 501-504):

"*In summary, calculating consumption-release and combining it with intracellular metabolomics via biological regulated clustering reveals metabolites of interest. Further combining these results with prior knowledge using the MetaproViz toolkit facilitates biological interpretation of the data.*"

(3)

3. Given the functional diversity among metabolites -central to diverse pathways, are key signaling molecules, restricted functions, co-variation within a pathway - I wonder how informative approaches such as PCA or enrichment analyses are for identifying metabolic drivers of a (patho)physiological state. To some extent, this can be addressed by integrating prior knowledge, and it would be helpful if the authors could comment on (and if applicable explain) whether/how this is integrated into MetaProViz.

The reviewer is correct in stating the functional diversity of metabolites, which is also why prior knowledge is needed to add mechanistic interpretation to the finding from the metadata analysis (as we showcased by focusing on the separation of age (**Fig. 5c-d**)). We think that approaches such as PCA or enrichment can be helpful, even if admittedly limited. For example, in the metadata analysis presented in **Fig. 5b** and the subsequent enrichment analysis presented in **Fig. 5**, we used PCA to extract the eigenvector and the loading, which act as weights indicating the contribution of each original metabolite to that specific principal components separation. Hence, the eigenvector of PCA shows the metabolite drivers of the separation. This does not necessarily mean that those metabolites are drivers of a (patho)physiological state - the (patho)physiological state can equally be the reason for those metabolites driving the separation on the Eigenvectors. Thus, the metadata analysis presented in **Fig. 5b** enables us to extract the metadata variables (patho)physiological states separated on a PC with the explained variance. This can also lead to co-variation, when multiple (patho)physiological states are separated on the same PC, as the reviewer correctly points out. Regarding the enrichment analysis, we provide

different types of prior knowledge for classical mapping, but also the prior knowledge we used to create the biological regulated clustering, which together help to identify key metabolic groups as we can first cluster the metabolites and afterwards perform functional enrichment. Yet, this does not account for the technical issues of enrichment analysis. In this context multi-omics integration building metabolic-centric networks could further elucidate the diversity of metabolic pathways and connection to signalling and co-variation, yet this is not the scope of MetaProViz. To sum up, we are aware of the limitations of this analysis and the constraints on the downstream interpretation.

To capture the functional diversity amongst metabolites, which leads to metabolites being present in multiple pathways of metabolite-pathways sets, we have implemented a new function to cluster metabolite-sets like pathways based on overlapping metabolites and visualize redundant metabolite-set (i.e. pathways) memberships (**Fig. 5f**). For more details also see our response to Reviewer 1, Comment 12. We hope this will help to avoid miss- or over-interpretation of the enrichment results.

In addition, we have extended the text to include the analysis pitfalls explicitly (Line 533-537): "*Another variable explaining the same amount of variance in PC1 is the tumour stage, which could point to adjacent normal tissue metabolic rewiring that happens in relation to stage and showcases that biological data harbour co-variations, which can not be disentangled by this method.*"

Reviewer #3

Evidence, reproducibility and clarity

This manuscript introduces an R package MetaProViz for metabolomics data analysis (post anotation), aiming to solve a poor-analysis-choices problem and enable more people to do the analysis. MetaProViz not only guides people to select the best statistical method, but also enables to solve previously unsolved problems: e.g. multiple and variable metabolite names in different databases and their connections to prior knowledge. They also created exometabolomics analysis and the needed steps to visualise intra-cell / media processes. The authors demonstrated their new package via kidney cancer (clear-cell renal cell carcinoma dataset, steping one step closer to improve biological interpretability of omics data analysis.

Significance

This is a great tool and I can't wait to use it on many upcoming metabolomics projects! Authors tackle multiple ongoing issues within the field: from poor selection of statistical methods (they provide guidance or have default safer options) to the messiness of data annotation between databases and improving data interpretability. The field is still evolving quickly, and it's impossible to solve all problems with one package; thus some limitations within the package could be seen as a bit rigid. Nonetheless, this fully steps toward filling an existing methodological gap. All

bioinformaticians doing metabolomic analysis, or those learning how to do it, will greatly benefit from this knowledge.

I myself lead a team of 6 bioinformaticians, and we do analysis for researchers, clinicians, drug discovery, and various companies. We run internal metabolomics pipelines every day and fully sympathise with the problems addressed by the authors.

Major comments affecting conclusions

none.

We thank the reviewer for this positive feedback on evidence, reproducibility and clarity as well as significance of our work given the reviewers experience with metabolomics data analysis mentioned. We appreciate that there are no major comments from the reviewer.

Minor comments

Minor comments, important issues that could be addressed and possibly improve the clarity or generally presentation of the tool. Please see all below.

(1)

1- You start with separating and talking about metabolomics and lipidomics, but lipidomics quickly dissapears (especially beyond abstract/intro) - no real need to discuss lipidomics.

Thanks, that's a good note and we have removed it from the abstract and introduction.

(2)

2- You refer to the MetImp4 imputation web tool, but I cannot find an active website, manuscript, or R package for it, and the cited link does not load. This raises doubts about whether the tool is currently usable. Additionally, imputation choice should be guided by biological context and study design, not just by testing a few methods and selecting the one that performs best.

We fully agree with the reviewer on imputation handling. The manuscript we cite from Wei et. al. (https://doi.org/10.1038/s41598-017-19120-0) compared a multitude of missing value imputation methods and made this comparison strategy available as a web-based tool not as any code-based package such as an R-package. Yet, the reviewer is right, the web-tool is no longer reachable. Hence, we have adapted the statement in our introduction (Line 62-67): "~~*Moreover, t*~~*There are tools that focus on specific steps of feature annotation (i.e. CAMERA (Kuhl et al., 2012), XCMS2 (Smith et al., 2006), and CliqueMS (Senan et al., 2019)) or the pre-processing of feature intensities, which encompasses feature selection, missing value imputation (MVI) (Wei et al., 2018) and data normalisation.* ~~*For example, MetImp4 is a web-tool that includes and compares multiple MVI methods(Wei et al., 2018).*~~ "

(3)

3- The authors address key metabolomics issues such as ambiguous metabolite names and isoforms, and their focus on resolving mapping ambiguities and translating between database identifiers is highly valuable. However, the larger challenge of de novo identification and the "dark matter" of unannotated metabolites remains unresolved (initiatives as MassIVE might help in the

future https://massive.ucsd.edu/ProteoSAFe/), and readers may benefit from clearer acknowledgement that MetaProViz does not operate on raw spectral data. The introduction currently emphasizes annotation, but since MetaProViz requires already annotated metabolite tables (and then deals with all the messiness), this space might be better used to frame the interpretability and pathway-analysis challenges that the tool directly addresses.

We appreciate the comment and have highlighted this in the abstract and introduction: "*MetaProViz operates on annotated intensity values…*" (Line 29 and 98).

Given the newest advancements in metabolite identification using AI-based methods, MetaProViz toolkit with a focus on connecting metabolite IDs to prior knowledge becomes increasingly valuable. We added this to our discussion (Line 615-619): "*Given the imminent shift in metabolite identification through AI-based approaches, including language model-guided[48] methods and self-supervised learning[49], the growing number of identified metabolites will make the MetaProViz toolkit increasingly valuable for the community to gain functional insights.*"

In regards to the introduction, where we mention some tools for peak annotation: The reason why we have this paragraph is that we wanted to set the basis by (I) listing the different steps of metabolomics data analysis and (II) pointing to well-known tools of those steps. We also have a dedicated paragraph for pathway-analysis challenges. Yet, we agree with the reviewer and have also minimized the content to focus on the workflow depiction without too much emphasis on peak annotation (Line 53-66):

"*The analysis of metabolomics data encompasses several steps starting with pre-processing (including ~~peak picking,~~ feature assembly and -annotation, feature filtering, etc.), followed by normalisation of intensities and statistical analysis, culminating in functional and mechanistic analyses to ultimately interpret the data (Chen, Li and Xu, 2022). ~~Examples of popular tools include CAMERA (Kuhl et al., 2012), XCMS2 (Smith et al., 2006), and CliqueMS (Senan et al., 2019) for the assembly, visualisation, and annotation of features. Some of them are also organised into workflows by other tools, for example MetaboAnalyst uses XCMS2 (Smith et al., 2006) for peak-integration and CAMERA (Kuhl et al., 2012) for peak-annotation (Pang et al., 2021; Pang, Lu, et al., 2024). Moreover, t~~There are tools that focus on specific steps of feature annotation (i.e. CAMERA (Kuhl et al., 2012), XCMS2 (Smith et al., 2006), and CliqueMS (Senan et al., 2019)) or the pre-processing of feature intensities, which encompasses feature selection, missing value imputation (MVI) (Wei et al., 2018) and data normalisation.*"

(4)

4- I also really enjoyed you touching on the point of user-friendly but then inflexible and problem of reproducibility. We truly need well working packages for other bioinformaticians, rather than expecting wet-lab scientists to do all the analysis within the user interface.

We thank the reviewer for this positive feedback.

(5)

5- It would be helpful to explain why the authors chose cancer/RCC samples for the demonstration. Was it because the dataset included both media and cell measurements? Does the tool perform best when multiple layers of information are available from the same experiment?

We specifically chose the ccRCC cell line data as an example since, for a multitude of cell lines, both media (exometabolomics) and intracellular metabolomics had been performed. The combination of both data types is only used in the biological regulated clustering (Fig. 5e-g), all other analyses do not require additional data modalities. We have not specifically tested how performance differs for this particular case as it would require multiple paired data (exometabolomics and intracellular metabolomics) taken at the same time and at different times.

We have added a sentence to ensure that only for the integration both modalities are needed (line 469-470):

"*If both exometabolomics and intracellular metabolomics has been performed, MetaProViz offers functional clustering to integrate those data modalities.*"

(6)

6- Figure 2B: The upset plots effectively show increased overlap after adaptation, but it would be easier to compare changes if the order of the intersection bars in the "adapted" plot matched the original. For example, while total intersections increased (251→285), the PubChem+KEGG overlap decreased (24→5), likely due to reallocation to the full intersection.

Thanks for raising this point. We initially had ordered the bars based on their intersection size, but we agree with the reviewers that for our point it would have made sense to fix the order in the adapted plot to match the order of the original plot. Yet, since we have now completely reworked this section and also added CHEBI IDs, previously not present, the overlaps now include four IDs. Hence, we did not reorder the plot, but we have added additional plots that show the deltas for each addition to make our point more clear (**see also response to your next minor comment 7**).

Adapted **Fig. 2b**:

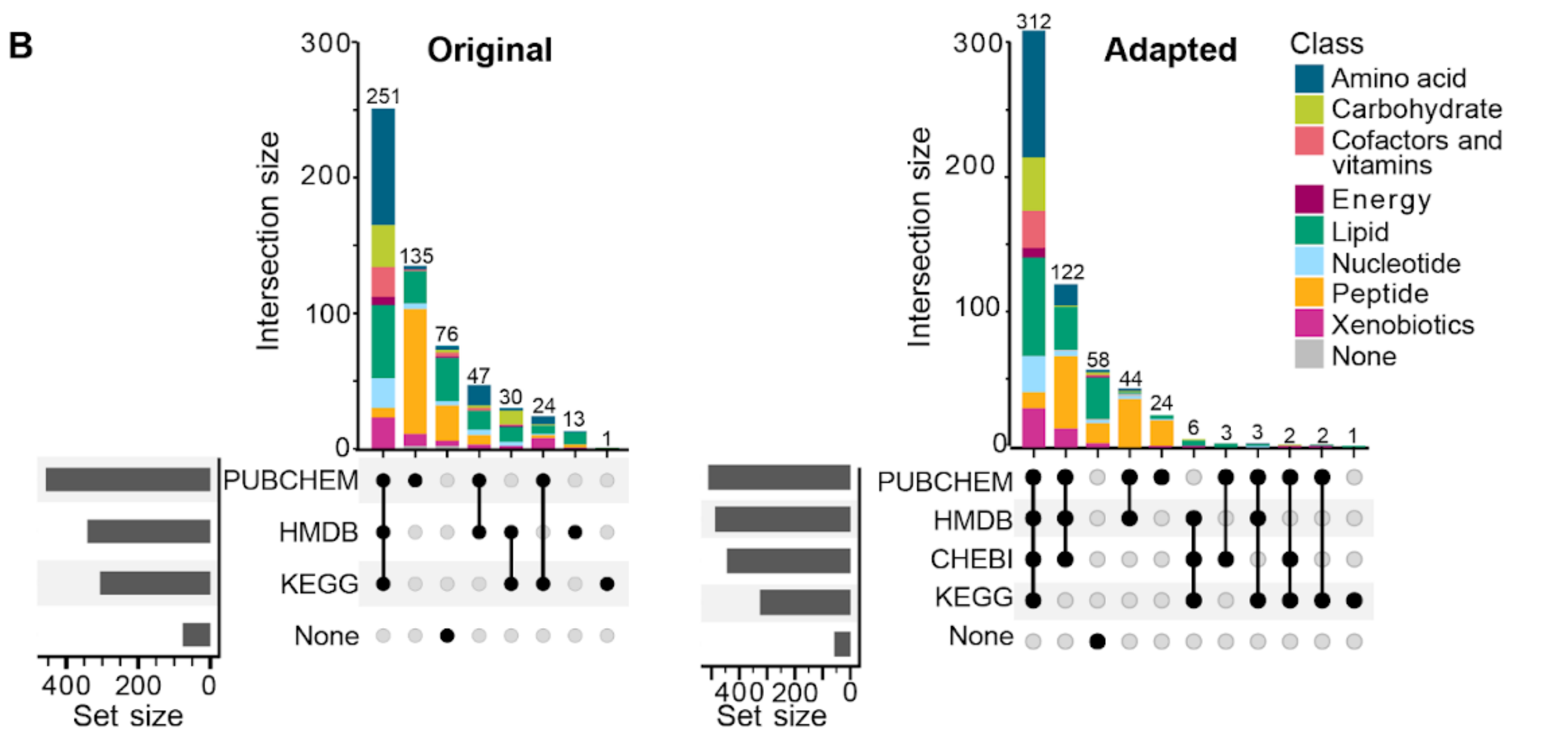

(7)

7- In your example of D-alanine and L-alanine - you mention how chirality is important biological feature, but up to this point it's not clear how do you do translation exactly and in which situations this would be treated just as "alanine" and when the more precise information would be retained? You mention RaMP-DB knowledge and one to X mappings as well as your general guidance in the "methods" part, but it would be useful to describe in this publication how you exactly tackled this problem in the ccRCC case.

We thank the reviewer for this suggestion. Since this is a complex problem, we added a more explicit description to the results section by showcasing more details on how we exactly tackled this problem in the ccRCC example data. Indeed, we have added a workflow overview and calculated the number of metabolite IDs added in each step separately for each ID type (KEGG, HMDB, PUBCHEM, CHEBI) as well as with multiplicity information. For details see response to **Reviewer 1** with **Comment 1** including details to workflow overview and **Comment 4** details to the new delta plots we created.

In regards to D- and L-alanine, even though chirality is an important biological feature, in a standard experiment we can not distinguish if we detect the L- or D-amino acid. This is why we try to assign all possible IDs to increase the overlap with the prior knowledge. In **Figure EV 1a** (previously **Fig. 2b)** we showcase that this can potentially lead to a feature matching different molecular entities in different pathways or within the same pathway. For example, if we measure alanine and assign the PubChem ID for L-Alanine, D-Alanine and Alanine and try to map to metabolite-sets that include L-Alanine and D-Alanine. In turn this could fall into Scenario 6 (**Fig. 2e**), where across pathways there is a D-Alanine specific one (Pathway 1) and a L-Alanine specific one (Pathway 2). Now we can decide, if we want to allow both mapping (many-to-one) or if we decide to exclude D-Alanine because we know our biological system is human and should primarily have L-Alanine.

(8)

8- In one to many mappings, it would be interesting to see quantification how frequently it was happening within a pathway or across pathways. I.e. Would going into pathway analysis "solve" the issue of "lost in translation" or not really?

We have quantified the frequency for the example of translating the KEGG metabolite-set into HMDB IDs (**Figure EV 1b**, originally **Fig. 2c**, left panel). Yet, we are not showcasing the quantification across the KEGG metabolite-sets with this plot. We have now restructured this information by adding additional columns for clarity in **Table EV2, sheet 24** (previously **Extended Data Table 2**).

We also added this to the results text (Line 277-279): *Indeed, mapping the ccRCC feature space to MetSigDB KEGG pathways we found 16 cases that required manual review, removing IDs from 14 of those cases.*

(9)

9- QC: the coefficient of variation (CV) helps identify features with high variability and thus low detection accuracy. Here it's important to acknowledge that if the feature is very variable between

groups it can be extremely important, but if the feature is very variable within the group - only then one would have low trust in the accuracy.

Yes, we totally agree with the reviewer on this. For this reason, we have applied CV only in instances where this is not leading to any condition-driven CV differences, but is truly feature-focused: (1) Function `pool_estimation` performs CV on the pool samples only, which are a homogeneous mixture of all samples, and hence can be used to assess feature variability. (2) Function `processing` performs CV on exometabolomics media samples (=blanks), which are also not impacted by different conditions.

(10)

10- Missing value imputation - while missing not at random is a great way to deal with missingness, it would be great to have options for others (not just MNAR), as missingness is of a complex nature. If a pretty strong decision has been made, it would be good to support this by some supplementary data (i.e. how results change while applying various combinations of missingness and why choosing MNAR seems to be the most robust).

We have decided to only offer support for MNAR, since we would recommend MVI only if there is a biological basis for it.

As mentioned in the response to your minor comment 2, Wei et. al. (https://doi.org/10.1038/s41598-017-19120-0) compared a multitude of missing value imputation methods. They compared six imputation methods (i.e., QRILC, Half-minimum, Zero, RF, kNN, SVD) for MNAR and systematically measured the performance of those imputation methods. They showed that QRILC and Half-Minimum produced much smaller SOR values, showing consistent good performances on data with different numbers of missing variables. This was the reason for us to only provide Half-minimum.

---

(11)

11- In the pre-processing and imputation stages - it would be interesting to see a summary table of how many features are left after each stage.

This is a good suggestion and refers to the steps described in **Fig. 3**. We have now added an additional sheet (sheet 4) to the **Table EV3** (previously **Extended Data Table 3**), which includes all measured features with a CV >30 in the pool estimation (**Fig. 3c**) or in the media blank (**Fig. 3d**) and the features with high missingness applying the modified 80% filtering rule (see methods section for details).

(12)

12- Is there a reason not to do UMAP or PSL-DA graphs for outlier detection? Doing more than PCA would help to have more confidence in removing or retaining outliers in the cases where biological relevance is borderline.

The reason we decided to use PCA was the standardly used combination with the Hotelling T2 outlier testing. Since PCA is a linear dimensionality reduction technique that preserves the overall variance in the data and has a clear mathematical foundation linked to the covariance structure,

it specifically fits the required assumptions of the Hotelling T2 outlier testing. Hotelling T2 relies on the properties of the covariance matrix and the assumption of a multivariate Gaussian distribution. UMAP is a non-linear dimensionality reduction technique, which prioritizes preserving local and global structures in a way that often results in good clustering visualization, but it distorts distances between clusters and does not have the same rigorous statistical underpinnings as PCA. In terms of PLS-DA, which focuses on maximizing the covariance between variables and the class labels, even though not commonly done, one could use the optimal latent variables for discrimination and apply Hotelling's T^2 to those latent variables. Yet, PLS-DA is supervised and actively tries to separate data points in the latent space, which can be misleading for outlier detection where methods like PCA that are unbiased and unsupervised and preserve global variance can be advantageous.
That said, we agree with the reviewer that UMAP and PLS-DA can be useful to evaluate the potential outliers in addition to using PCA alone. PLS-DA can be especially useful if due to the biological context it is relevant to define certain class labels. It is available in other R-packages (e.g. mixOmics https://mixomics.org/methods/spls-da/), that could be combined by users with MetaProviz.

As discussed in the text, the outliers found with Hotelling T2 test do not have to be removed, as in our case we decided against removing MS51-09 (line 416-424):

"*Once the data processing and normalisation is complete, Hotelling's T2 test (Hotelling, 1931) is used to detect outliers across the multidimensional space of PCA (**Fig. 3g, ~~S~~Fig. EV2~~3~~d-e**). It is up to the user to remove any sample outliers, but we recommend removing major outliers identified in the filtering round 1 (**Fig. 3g**). Here it can also be helpful to use biological information such as the experimental conditions of the cell lines in this example (**Fig. 3g** and **Table EV3~~Supplementary Table~~**). In this case, it becomes clear that sample MS51-09, identified as potential outlier in filtering round 2, clusters together with replicates of the same cell line, hence we would opt to not remove it.*"

---

(13)

13- Metadata vs metabolite features - can this be used beyond metabolomics (i.e. proteomics, transcriptomics, etc)? It can be always very useful when there are many metadata features and it's hard to pre-select beforehand which ones are the most biologically relevant.

Yes, definitely. In fact, we have used the metadata analysis strategy also with proteomics data and it will work equally with any omics data type.

(14)

14- While authors discussed what KEGG pathways were significantly deregulated, it would be interesting to see all the pathways that were affected (e.g. aPEAR "bubble" graphs can show this (https://github.com/kerseviciute/aPEAR) , or something similar to NES scores). I appreciate the trickiness of it, but it would be quite interesting to see how authors e.g. Figure5e narrowed it down to the two pathways and how all the others looked like.

We thank the reviewer for the suggestion of the aPEAR graphs. Following this suggestion, we have implemented a new function to enable clustering of the pathways based on overlapping metabolites (`cluster_pk()`). For more details regarding the method see also our response to Reviewer 1 (Comment 12) and our extended method section "Metabolite-set clustering" (Lines 830-845). We visualize the clustering results as a network graph, which we also included into **Fig. 5f**.

The complete result of the KEGG enrichment can be found in Extended Data Table 1, Sheet 13 (Pathway enrichment analysis using KEGG on Young patient subset). The pathways are ranked by p.adjusted value and also include a score (FoldEnrichment) from the fishers exact test (similar to NES scores in GSEA). Here one can find a total of seven pathways with a p.adjusted value < 0.05, of which we showcase two in **Fig. 5e**

For **Fig. 5e** we narrowed down to these two pathways based on the previous findings of dysregulated dipeptides (**Fig. 5d**), as we searched for a potential explanation of this observation.

(15)

15- Could you comment on the runtime of the pipeline? In particular, do the additional translation steps and use of multiple databases substantially affect computational speed?

Downloading and parsing databases takes significant time, especially large ones like RaMP or HMDB might take minutes on a standard laptop. Our local cache speeds up the process by eliminating the need for repeated downloads. In the future, database access will be even faster: according to our plans, all prior knowledge will be accessible in an already parsed format by our own API (omnipathdb.org). The ambiguity analysis, which is a complex data transformation pipeline, and plotting by ggplot2, another key component of MetaProViz, are the slowest parts, especially when performing analysis for the first time when no cache can be used. This means there are a few slow operations which complete in maximum a few dozens of seconds. However, the implementation and speed of these solutions doesn't fall behind what we commonly find in bioinformatics packages, and most importantly, the speed of MetaProViz doesn't pose an obstacle or difficulty regarding an efficient use of it in analysis pipelines.

(16)

16- I clap to the authors for automated checks if selected methods are appropriate!

Thank you, this is something we think is important to ensure correct analysis and circumvent misinterpretation.

(17)

17- My suggestion would be to also look into power calculation or p-value histogram. In your example you saw some clear signal, but very frequently research studies are under-sampled and while effect can be clearly seen, there are just not enough samples to have statistically significant hits.

We fully agree that power calculations are very important. Yet, this should ideally happen prior to the user's experiment. MetaProViz analysis starts at a later time-point and power calculations

should have been done before. In regards to p-value histogram, we have implemented a similar measure, namely a density plot, which is plotted as a quality control measure within MetaProViz differential analysis function. The density plot is a smoothed version of a histogram that represents the distribution as a continuous probability density function and can be used to assess whether the p-values follow a uniform distribution.

(18)

18- Overall functional parts are novel and next step in helping with data interpretability, but I still found it hard to read into functionally clear insights (re to pathways / functional groupings of metabolites) - especially as you have e.g. enzyme-metabolite databases etc. I think clarity there could be improved and would help to get your message more widely across.

Regarding the clarity to the pathway enrichment and their functional insights, we have extended the Figure legends of **Fig. 4** and **5**, clearly stating that for the functional interpretation MetalinkDB is the prior knowledge resource we used to identify the links for methionine (Line 488-492):

"*To explore the role of these metabolites in signaling, we queried the prior knowledge resource MetalinksDB (Farr et al., 2024), which includes metabolite-receptor, metabolite-transporter and metabolite-enzyme relationships, for their known upstream and downstream protein interactors for the measured metabolites (**Table EV3**~~**Supplementary Table 5**~~).*"

We have also extended our summary statement to highlight that we combine the biological clustering with prior knowledge for the mechanistic insight (Line 501-504):

"*In summary, calculating consumption-release and combining it with intracellular metabolomics via biological regulated clustering reveals metabolites of interest. Further combining these results with prior knowledge using the MetaproViz toolkit facilitates biological interpretation of the data*."

28th May 2026

Manuscript Number: MSB-2026-13622R
Title: Integrated metabolomics data analysis to generate mechanistic hypotheses with MetaProViz

Dear Dr. Saez-Rodriguez,

Thank you for the submission of your revised manuscript to Molecular Systems Biology. We have now received the enclosed reports from the referees that were asked to re-assess it. As you will see the reviewers are now globally supportive and I am pleased to inform you that we will be able to accept your manuscript pending the following final amendments:

1) In the "Author Checklist", please include the manuscript ID number.
2) Please provide a 'clean' main manuscript file, without tracked changes.
3) Please include keywords to max. 5.
4) Please rename "Competing Interests" to "Disclosure and competing interests statement". We updated our journal's competing interests policy in January 2022 and request authors to consider both actual and perceived competing interests. Please review the policy https://link.springer.com/partners/embo-press/editorial-policies#Competing%20interest%20disclosures and update your competing interests if necessary.
5) Please correct the reference citation in the reference list such that when there there are more than 10 authors on a paper, only the first 10 should be listed, followed by "et al.". DOIs should only be used for preprints and datasets that have not been published yet. Please check "Author Guidelines" for more information: https://link.springer.com/journal/44320/submission-guidelines#cms-Reference-guidelines
6) Our journal encourages inclusion of *data citations in the reference list* to directly cite datasets that were re-used and obtained from public databases. Data citations in the article text are distinct from normal bibliographical citations and should directly link to the database records from which the data can be accessed. In the main text, data citations are formatted as follows: "Data ref: Smith et al, 2001" or "Data ref: NCBI Sequence Read Archive PRJNA342805, 2017". In the Reference list, data citations must be labeled with "[DATASET]". A data reference must provide the database name, accession number/identifiers and a resolvable link to the landing page from which the data can be accessed at the end of the reference. Further instructions are available at: https://link.springer.com/journal/44320/submission-guidelines#cms-Reference-guidelines
7) In the Methods, please take care of the following:
- The Materials and Methods section should be renamed to "Methods".
- Please ensure that a statement on whether or not blinding was done is included in the Methods even if no blinding was done. Please also be sure to update the Author Checklist with this information and where it can be found in the manuscript.
8) Please remove the Reagents and Tools table from the manuscript, as you have already provided it as a separate file. This table should not be identified as Table 1, therefore all subsequent tables will need to be renumbered and their callouts updated in the manuscript.
9) Please place individual sections of the manuscript in the following order: Title page - Abstract & Keywords - Introduction - Results - Discussion - Methods - Data Availability - Acknowledgements - Disclosure and Competing Interests Statement - References - Figure Legends - (Main Tables with legends if applicable) - Expanded View Figure Legends.
10) For the figures and figure legends, please take care of the following:
- Please make sure to update the callouts of all figures in the main manuscript text. Currently figure callouts are missing for Figures 4G and 5D-F.
- Please note that the box plots need to be defined in terms of minima, maxima, centre, bounds of box and whiskers, and percentile in the legends of figures 3F.
- Please note that information related to n is missing in the legends of 3F; 4H; 5D,E.
- Please note that the error bars are not defined in the legends of figure 4H.
11) Please rename Table EV1-EV4 to Dataset EV1-EV4 and ensure that the legend is included the corresponding file in a separate tab. Please also be sure to update their callouts in main manuscript text.
12) The individual figure file for Appendix Figure S1 is not needed, only the Appendix PDF is sufficient. Please remove tracked changes from Appendix file.
13) Please ensure that all funding sources are entered correctly into the manuscript submission system. 'Landesinstitut für Bioinformatikinfrastruktur in Baden-Württemberg' should be updated to be correctly inserted in the system.
14) Please reformat the synopsis image to our required dimensions of 550 pixels wide x 300 pixels high.
15) Source Data should be organized as a single source data file (zipped) per figure for main figures (all EV and/or Appendix figure Source Data can be included in a single folder), with the panels clearly visible in the folder structure e.g. all the Source data files for figure 1 need to be saved in a single folder and this needs to be zipped and then uploaded as "SD figure 1.zip" file.
16)
17) As part of the EMBO Publications transparent editorial process initiative (see our policy here: https://www.embopress.org/transparent-process#Review_Process), Molecular Systems Biology will publish online a Peer Review File (PRF) to accompany accepted manuscripts. This file will be published in conjunction with your paper and will include the anonymous referee reports, your point-by-point response and all pertinent correspondence relating to the manuscript. Let us know whether you agree with the publication of the PRF and as here, if you want to remove or not any figures from it prior to

publication. Please note that the Authors checklist will be published at the end of the PRF.
18) After your paper is published, we may promote it on social media. If you have any handles or hashtags for Bluesky you would like included, please let us know.
19) Please provide a point-by-point letter INCLUDING my comments and your detailed responses (as Word file).

I look forward to reading a new revised version of your manuscript as soon as possible.

Yours sincerely,

Poonam Bheda, PhD
Scientific Editor
Molecular Systems Biology

Reviewer #1:

Authors have addressed my concerns and I believe the manuscript is now suitable for publication.

Reviewer #2:

The authors have fully addressed my comments.

Reviewer #3:

MetaProViz is a long needed resource and tool for the metabolomics data analysis, combining knowledge from multiple databases and assisting the deeper interpretation of the analysis results nad insights.

Since my comments have been addressed and answered in detailed manner (from the previous round of the review), I suggest to process with the publication of this manuscript, as there is always further possible developments (e.g. benchmarking with other available commonly used workflows) possible and, as a researcher, I much rather the community gets to read and use this tool asap.

Point-by-point letter

Point-by-point letter including the editors' comments and our detailed responses:

- Editor: plain text highlighted in grey
- Authors: Blue text

Comments:

1) In the "Author Checklist", please include the manuscript ID number.

We have added the Manuscript ID number to the "Authors Checklist"

2) Please provide a 'clean' main manuscript file, without tracked changes.

We have removed tracked changes and line numbers from the manuscript file.

3) Please include keywords to max. 5.

We have included keywords within the Manuscript file below the Abstract:

"Metabolomics, Exometabolomics, Prior Knowledge Integration, Functional Analysis, Data Visualisation"

4) Please rename "Competing Interests" to "Disclosure and competing interests statement". We updated our journal's competing interests policy in January 2022 and request authors to consider both actual and perceived competing interests. Please review the policy https://link.springer.com/partners/embo-press/editorial-policies#Competing%20interest%20disclosures and update your competing interests if necessary.

We have renamed "Competing Interests" to "Disclosure and competing interests statement" within the Manuscript file. We have reviewed the policy.

5) Please correct the reference citation in the reference list such that when there there are more than 10 authors on a paper, only the first 10 should be listed, followed by "et al.". DOIs should only be used for preprints and datasets that have not been published yet. Please check "Author Guidelines" for more information: https://link.springer.com/journal/44320/submission-guidelines#cms-Reference-guidelines

We have updated our references accordingly following the guidelines.

6) Our journal encourages inclusion of *data citations in the reference list* to directly cite datasets that were re-used and obtained from public databases. Data citations in the article text are distinct from normal bibliographical citations and should directly link to the database records from which the data can be accessed. In the main text, data citations are formatted as follows:

"Data ref: Smith et al, 2001" or "Data ref: NCBI Sequence Read Archive PRJNA342805, 2017". In the Reference list, data citations must be labeled with "[DATASET]". A data reference must provide the database name, accession number/identifiers and a resolvable link to the landing page from which the data can be accessed at the end of the reference. Further instructions are available at: https://link.springer.com/journal/44320/submission-guidelines#cms-Reference-guidelines

Thanks for pointing us to this guidelines again, as we have initially misunderstood how to do this correctly. In the case where data was accessed via accession number, we have created a data citation. For the cases where we have used the supplementary table provided as part of a journal publication we have not used a dedicated "DATASET" citation.

*"Example Data. MetaProViz provides access to diverse example datasets from kidney cancer studies. Cell-line data include intracellular metabolomics of human renal epithelial cells (HK2) and renal cell carcinoma (ccRCC) cell lines 786-O, 786-M1A, and 786-M2A and corresponding cell culture media profiling (exometabolomics), which additionally includes ccRCC cell lines OS-RC-2, OS-LM1, and RFX-631 were downloaded from metabolomics workbench project PR001418 including study ST002224 and ST002226 (**Data ref: Yang, 2022**; Sciacovelli et al., 2022). Patient tissue metabolomics from 138 matched ccRCC and adjacent normal tissue pairs were downloaded from Hakimi et al. "supplementary table 2", which includes median normalized data(Hakimi et al., 2016). Patient tissue processed transcriptomics and proteomics data from ccRCC tissue were downloaded from Mora&Schmidt et al table "13073_2024_1415_MOESM3_ESM.xlsx" originally from proteomics Data Commons accession number PDC000127 (Clark et al., 2019; Mora et al., 2024; **Data ref: Clark et al., 2019**). The detailed code can be found on the "ExampleData" branch of the MetaProViz package (https://github.com/saezlab/MetaProViz/tree/ExampleData)."*

- Clark DJ, Dhanasekaran SM, Petralia F, Pan J, Song X, Hu Y, da Veiga Leprevost F, Reva B, Lih TM, Chang H, et.al (2019),(https://pdc.cancer.gov/pdc/study/PDC000127). [DATASET]
- Yang M (2022) Metabolomics Workbench Project PR001418 (http://dx.doi.org/10.21228/M8F409). [DATASET]

7) In the Methods, please take care of the following: The Materials and Methods section should be renamed to "Methods". Please ensure that a statement on whether or not blinding was done is included in the Methods even if no blinding was done. Please also be sure to update the Author Checklist with this information and where it can be found in the manuscript.

We have renamed "Materials and Methods" to "Methods" within our Manuscript file.

We have now added an explicit blinding statement to the Methods section and refer to this within the Author Checklist:

*"**Blinding.** No blinding was performed in this study. This work is a secondary analysis of publicly available datasets, and no experimental allocation, intervention, or outcome assessment requiring blinding was conducted by the authors."*

8) Please remove the Reagents and Tools table from the manuscript, as you have already provided it as a separate file. This table should not be identified as Table 1, therefore all subsequent tables will need to be renumbered and their callouts updated in the manuscript.

We have removed the "Reagents and Tools table" from the Manuscript and renamed the subsequent tables as well as their callouts within the text.

9) Please place individual sections of the manuscript in the following order: Title page - Abstract & Keywords - Introduction - Results - Discussion - Methods - Data Availability - Acknowledgements - Disclosure and Competing Interests Statement - References - Figure Legends - (Main Tables with legends if applicable) - Expanded View Figure Legends.

We have reordered our Manuscript following this provided order.

10) For the figures and figure legends, please take care of the following:

- Please make sure to update the callouts of all figures in the main manuscript text. Currently figure callouts are missing for Figures 4G and 5D-F.
- Please note that the box plots need to be defined in terms of minima, maxima, centre, bounds of box and whiskers, and percentile in the legends of figures 3F.
- Please note that information related to n is missing in the legends of 3F; 4H; 5D,E.
- Please note that the error bars are not defined in the legends of figure 4H.

We have adapted the figure legends accordingly:

- Figure callouts for Figures 4G and 5D-F were added to the text.
- Information related to the box plots of minima, maxima, centre, bounds of box and whiskers, and percentile was added to the figure legend of 3F: *"The centre line indicates the median, box boundaries represent the 25th and 75th percentiles, and whiskers extend to the most extreme observations within 1.5 times interquartile range. Values beyond the whiskers are shown as outliers."*
- Information related to n was added to figures 3F, 4H, 5D,5E:
    - 3F: *"Boxplots of feature intensities before and after TIC normalisation (media blank = lightblue; HK2 = blue; 786-O, OSRC2, RFX631= light to darkpink; 786-M1A, 786-M2A, OSLM1B= light to darkgreen) (**n=5 biological replicates per condition**)."*
    - 4H: *"Bar graphs of normalised methionine intensity for exometabolomics with **n=5 biological replicates per condition** (CoRe: negative value, if the metabolite has been consumed from the media, or a positive value, if the metabolite has been released from the cell into the culture media) and intracellular metabolomics with **n=3 biological replicates per condition** (Intra)."*
    - 5D: *"Volcano plot of tumour versus normal metabolite changes in old (triangle, **n=88 patients**) and young (square, **n=8 patients**) ccRCC patients colour coded by biological clusters using limma and false discovery rate (FDR) for p-value adjustment."*

- 5H: *"Volcano plots of tumour versus normal metabolite changes in young patients (**n=8 patients**) (limma and false discovery rate (FDR) for p-value adjustment) within two KEGG pathways that were significantly deregulated based on over representation analysis."*
- Information related to the error bars were added in the legends of figure 4H *"Bars represent the mean and error bars indicate ± SD."*

11) Please rename Table EV1-EV4 to Dataset EV1-EV4 and ensure that the legend is included the corresponding file in a separate tab. Please also be sure to update their callouts in main manuscript text.

We have renamed our Tables to Datasets and their callout within the Manuscript file. We added a legend onto the first sheet of each dataset (EV1-4).

12) The individual figure file for Appendix Figure S1 is not needed, only the Appendix PDF is sufficient. Please remove tracked changes from Appendix file.

We have removed track changes from the Appendix file and we will only upload the PDF.

13) Please ensure that all funding sources are entered correctly into the manuscript submission system. 'Landesinstitut für Bioinformatikinfrastruktur in Baden-Württemberg' should be updated to be correctly inserted in the system.

This funding source is correct and the official name of the funding. We are unsure how to proceed in this regard.

14) Please reformat the synopsis image to our required dimensions of 550 pixels wide x 300 pixels high.

We have reformatted the synopsis image to the required pixels:

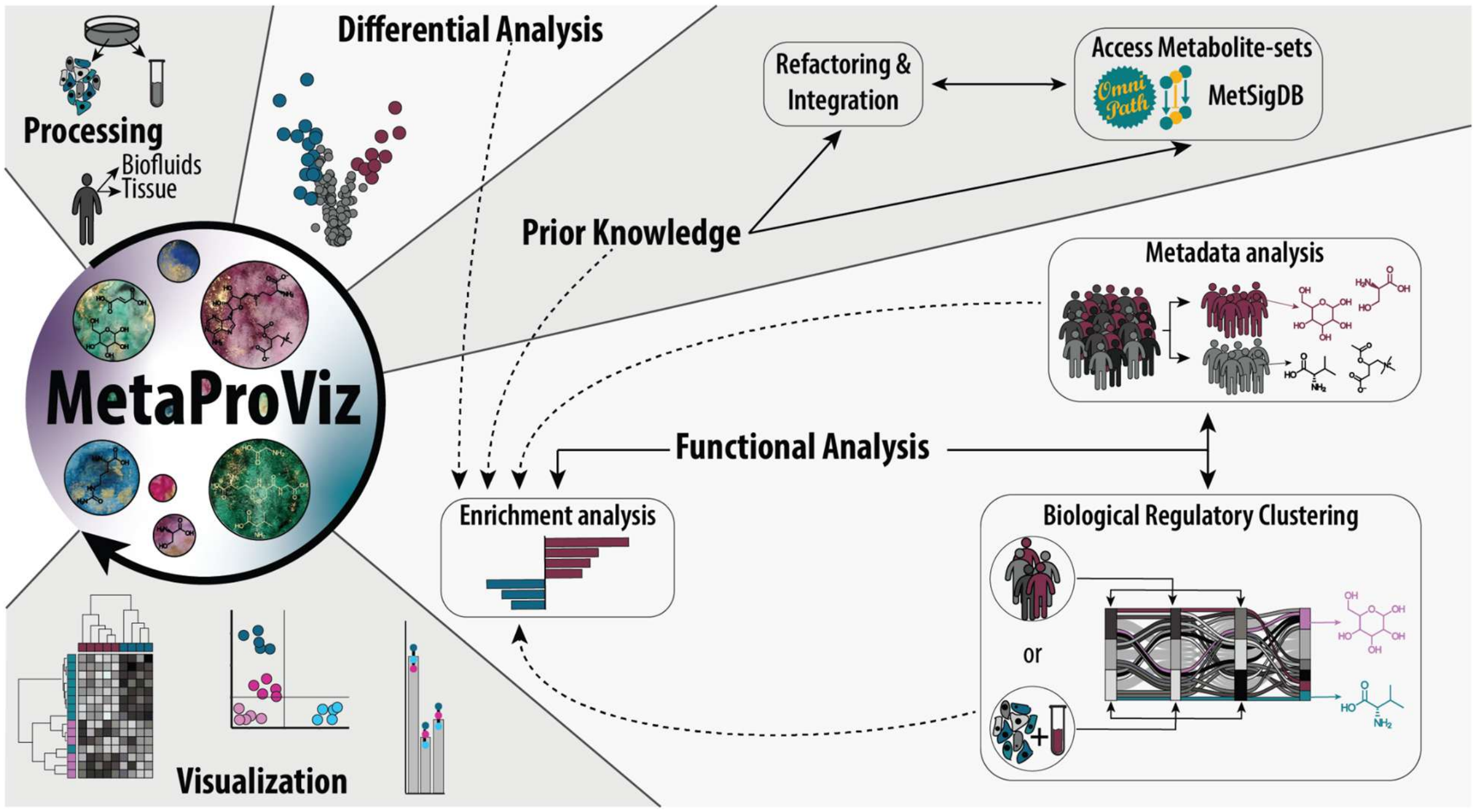

15) Source Data should be organized as a single source data file (zipped) per figure for main figures (all EV and/or Appendix figure Source Data can be included in a single folder), with the panels clearly visible in the folder structure e.g. all the Source data files for figure 1 need to be saved in a single folder and this needs to be zipped and then uploaded as "SD figure 1.zip" file.

We have now revised and reorganized all Source Data files to match the journal's instructions. Specifically, we are now providing:

1. One zipped Source Data package per main figure, named as requested (for example, SD figure 1.zip, SD figure 2.zip, etc.).
2. Within each main figure package, all panel-level source data CSV files are placed directly in a single folder for that figure, with panel identity clearly indicated in the filenames.
3. Extended/EV figure Source Data organized in a single combined package.

In other words, all source files for each main figure are now consolidated into one figure-specific folder and zipped as one file per figure, as requested.

16) .

Point 16 had no content, so we ignored it.

17) As part of the EMBO Publications transparent editorial process initiative (see our policy here: https://www.embopress.org/transparent-process#Review_Process), Molecular Systems Biology will publish online a Peer Review File (PRF) to accompany accepted manuscripts. This file will be published in conjunction with your paper and will include the anonymous referee reports, your point-by-point response and all pertinent correspondence relating to the manuscript. Let us know whether you agree with the publication of the PRF and as here, if you want to remove or not any figures from it prior to publication. Please note that the Authors checklist will be published at the end of the PRF.

We agree with the publication of the PRF.

18) After your paper is published, we may promote it on social media. If you have any handles or hashtags for Bluesky you would like included, please let us know.

Yes, we do have handles for Bluesky:

- Shared corresponding authors:
    1. Julio Saez-Rodriguez leading the Saez-Rodriguez Lab: **@saezlab.bsky.social**
    2. Christian Frezza leading the Frezza Lab: **@frezzalab.bsky.social**
- First author:
    1. Christina Schmidt: **@christinaschmidt.bsky.social**

Here are some hashtags that could be used:

#metabolomics

#exometabolomics

#Prior Knowledge Integration

#Functional Analysis

#Visualisation

19) Please provide a point-by-point letter INCLUDING my comments and your detailed responses (as Word file).

With this file, we are providing our point-to-point response to the comments of the Editor.

24th Jun 2026
Manuscript number: MSB-2026-13622RR
Title: Integrated metabolomics data analysis to generate mechanistic hypotheses with MetaProViz

Dear Dr. Saez-Rodriguez,

Thank you again for sending us your revised manuscript. We are now satisfied with the modifications made and I am pleased to inform you that your paper has been accepted for publication.

Your manuscript will be processed for publication by EMBO Press. It will be copy edited and you will receive page proofs prior to publication. Please note that you will be contacted by Springer Nature Author Services to complete licensing and payment information.

You may qualify for financial assistance for your publication charges - either via a Springer Nature fully open access agreement or an EMBO initiative. Check your eligibility: https://link.springer.com/journal/44320/how-to-publish-with-us

Should you be planning a Press Release on your article, please notify the production team via this form in order to coordinate publication and release dates: https://forms.cloud.microsoft/e/eH4A8JVP6g

If you have any questions, please do not hesitate to contact the Editorial Office. Thank you for your contribution to Molecular Systems Biology.

Yours sincerely,

Poonam Bheda, PhD
Scientific Editor
Molecular Systems Biology

>>> Please note that it is Molecular Systems Biology policy for the transcript of the editorial process (containing referee reports and your response letter) to be published as an online supplement to each paper. If you do NOT want this, you will need to inform the Editorial Office via email immediately. More information is available here: https://link.springer.com/partners/embo-press/editorial-policies#Peer%20review

EMBO Press Author Checklist

Corresponding Author Name: Christian Frezza and Julio Saez-Rodriguez	
Journal Submitted to: Molecular Systems Biology	
Manuscript Number: MSB-2026-13622-T	

USEFUL LINKS FOR COMPLETING THIS FORM
The EMBO Journal - Author Guidelines
EMBO Reports - Author Guidelines
Molecular Systems Biology - Author Guidelines
EMBO Molecular Medicine - Author Guidelines

Reporting Checklist for Life Science Articles (updated January
This checklist is adapted from Materials Design Analysis Reporting (MDAR) Checklist for Authors. MDAR establishes a minimum set of requirements in transparent reporting in the life sciences (see Statement of Task: 10.31222/osf.io/9sm4x). Please follow the journal's guidelines in preparing your manuscript.
Please note that a copy of this checklist will be published alongside your article.

Abridged guidelines for figures
1. Data
The data shown in figures should satisfy the following conditions:
- the data were obtained and processed according to the field's best practice and are presented to reflect the results of the experiments in an accurate and unbiased manner.
- ideally, figure panels should include only measurements that are directly comparable to each other and obtained with the same assay.
- plots include clearly labeled error bars for independent experiments and sample sizes. Unless justified, error bars should not be shown for technical
- if n<5, the individual data points from each experiment should be plotted. Any statistical test employed should be justified.
- Source Data should be included to report the data underlying figures according to the guidelines set out in the authorship guidelines on Data

2. Captions
Each figure caption should contain the following information, for each panel where they are relevant:
- a specification of the experimental system investigated (eg cell line, species name).
- the assay(s) and method(s) used to carry out the reported observations and measurements.
- an explicit mention of the biological and chemical entity(ies) that are being measured.
- an explicit mention of the biological and chemical entity(ies) that are altered/varied/perturbed in a controlled manner.
- the exact sample size (n) for each experimental group/condition, given as a number, not a range;
- a description of the sample collection allowing the reader to understand whether the samples represent technical or biological replicates (including how many animals, litters, cultures, etc.).
- a statement of how many times the experiment shown was independently replicated in the laboratory.
- definitions of statistical methods and measures:

- common tests, such as t-test (please specify whether paired vs. unpaired), simple χ2 tests, Wilcoxon and Mann-Whitney tests, can be unambiguously identified by name only, but more complex techniques should be described in the methods section;

- are tests one-sided or two-sided?
- are there adjustments for multiple comparisons?
- exact statistical test results, e.g., P values = x but not P values < x;
- definition of 'center values' as median or average;
- definition of error bars as s.d. or s.e.m.

Please complete ALL of the questions below.
Select "Not Applicable" only when the requested information is not relevant for your study.

Materials

Newly Created Materials	Information included in the manuscript?	In which section is the information available? (Reagents and Tools Table, Materials and Methods, Figures, Data Availability Section)
New materials and reagents need to be available; do any restrictions apply?	Not Applicable	

Antibodies	Information included in the manuscript?	In which section is the information available? (Reagents and Tools Table, Materials and Methods, Figures, Data Availability Section)
For **antibodies** provide the following information:<br>- Commercial antibodies: RRID (if possible) or supplier name, catalogue number and or/clone number<br>- Non-commercial: RRID or citation	Not Applicable	

DNA and RNA sequences	Information included in the manuscript?	In which section is the information available? (Reagents and Tools Table, Materials and Methods, Figures, Data Availability Section)
**Short novel DNA or RNA including primers, probes**: provide the sequences.	Not Applicable	

Cell materials	Information included in the manuscript?	In which section is the information available? (Reagents and Tools Table, Materials and Methods, Figures, Data Availability Section)
**Cell lines:** Provide species information, strain. Provide accession number in repository **OR** supplier name, catalog number, clone number, and/**OR** RRID.	Not Applicable	
**Primary cultures:** Provide species, strain, sex of origin, genetic modification status.	Not Applicable	
Report if the cell lines were recently **authenticated** (e.g., by STR profiling) and tested for mycoplasma contamination.	Not Applicable	

Experimental animals	Information included in the manuscript?	In which section is the information available? (Reagents and Tools Table, Materials and Methods, Figures, Data Availability Section)
**Laboratory animals or Model organisms:** Provide species, strain, sex, age, genetic modification status. Provide accession number in repository **OR** supplier name, catalog number, clone number, **OR** RRID.	Not Applicable	
**Animal observed in or captured from the field:** Provide species, sex, and age where possible.	Not Applicable	
Please detail **housing and husbandry conditions**.	Not Applicable	

Plants and microbes	Information included in the manuscript?	In which section is the information available? (Reagents and Tools Table, Materials and Methods, Figures, Data Availability Section)
**Plants:** provide species and strain, ecotype and cultivar where relevant, unique accession number if available, and source (including location for collected wild specimens).	Not Applicable	
**Microbes:** provide species and strain, unique accession number if available, and source.	Not Applicable	

Human research participants	Information included in the manuscript?	In which section is the information available? (Reagents and Tools Table, Materials and Methods, Figures, Data Availability Section)
If collected and within the bounds of privacy constraints report on age, sex and gender or ethnicity for all study participants.	Not Applicable	

Core facilities	Information included in the manuscript?	In which section is the information available? (Reagents and Tools Table, Materials and Methods, Figures, Data Availability Section)
If your work benefited from core facilities, was their service mentioned in the acknowledgments section?	Not Applicable	

Design

Study protocol	Information included in the manuscript?	In which section is the information available? (Reagents and Tools Table, Materials and Methods, Figures, Data Availability Section)
If study protocol has been **pre-registered, provide DOI in the manuscript**. For clinical trials, provide the trial registration number **OR** cite DOI.	Not Applicable	
Report the **clinical trial registration number** (at ClinicalTrials.gov or equivalent), where applicable.	Not Applicable	

Laboratory protocol	Information included in the manuscript?	In which section is the information available? (Reagents and Tools Table, Materials and Methods, Figures, Data Availability Section)
Provide DOI OR other citation details if **external detailed step-by-step protocols** are available.	Not Applicable	

Experimental study design and statistics	Information included in the manuscript?	In which section is the information available? (Reagents and Tools Table, Materials and Methods, Figures, Data Availability Section)
Include a statement about **sample size** estimate even if no statistical methods were used.	Not Applicable	
Were any steps taken to minimize the effects of subjective bias when allocating animals/samples to treatment (e.g. **randomization procedure**)? If yes, have they been described?	Not Applicable	
Include a statement about **blinding** even if no blinding was done.	Yes	At the end of the methods section we included the following statement: "No blinding was performed in this study. This work is a secondary analysis of publicly available datasets, and no experimental allocation, intervention, or outcome assessment requiring blinding was conducted by the authors."
Describe **inclusion/exclusion criteria** if samples or animals were excluded from the analysis. Were the criteria pre-established?<br><br>If sample or data points were omitted from analysis, report if this was due to attrition or intentional exclusion and provide justification.	Yes	Samples that were excluded from the study were detected as outliers using the Hotellins T2 test. We have specifically stated the removal in the methods section under "Outlier detection".
For every figure, are **statistical tests** justified as appropriate? Do the data meet the assumptions of the tests (e.g., normal distribution)? Describe any methods used to assess it. Is there an estimate of variation within each group of data? Is the variance similar between the groups that are being statistically compared?	Yes	Figures, Materials and Methods

Sample definition and in-laboratory replication	Information included in the manuscript?	In which section is the information available? (Reagents and Tools Table, Materials and Methods, Figures, Data Availability Section)
In the figure legends: state number of times the experiment was **replicated** in laboratory.	Not Applicable	
In the figure legends: define whether data describe **technical or biological replicates**.	Yes	Figures

Ethics

Ethics	Information included in the manuscript?	In which section is the information available? (Reagents and Tools Table, Materials and Methods, Figures, Data Availability Section)
Studies involving **human participants**: State details of **authority granting ethics approval** (IRB or equivalent committee(s), provide reference number for approval.	Not Applicable	
Studies involving **human participants**: Include a statement confirming that **informed consent** was obtained from all subjects and that the experiments conformed to the principles set out in the WMA Declaration of Helsinki and the Department of Health and Human Services Belmont Report.	Not Applicable	
Studies involving **human participants:** For publication of **patient photos**, include a statement confirming that consent to publish was obtained.	Not Applicable	
Studies involving experimental **animals**: State details of **authority granting ethics approva**l (IRB or equivalent committee(s), provide reference number for approval. Include a statement of compliance with ethical regulations.	Not Applicable	
Studies involving **specimen and field samples:** State if relevant **permits** obtained, provide details of authority approving study; if none were required, explain why.	Not Applicable	

Dual Use Research of Concern (DURC)	Information included in the manuscript?	In which section is the information available? (Reagents and Tools Table, Materials and Methods, Figures, Data Availability Section)
Could your study fall under dual use research restrictions? Please check biosecurity documents and list of **select agents and toxins** (CDC): https://www.selectagents.gov/sat/list.htm	Not Applicable	
If you used a select agent, is the security level of the lab appropriate and reported in the manuscript?	Not Applicable	
If a study is subject to dual use research of concern regulations, is the name of the **authority granting approval and reference number** for the regulatory approval provided in the manuscript?	Not Applicable	

Reporting

The MDAR framework recommends adoption of discipline-specific guidelines, established and endorsed through community initiatives. Journals have their own policy about requiring specific guidelines and recommendations to complement MDAR.

Adherence to community standards	Information included in the manuscript?	In which section is the information available? (Reagents and Tools Table, Materials and Methods, Figures, Data Availability Section)
State if relevant guidelines or checklists (e.g., **ICMJE, MIBBI, ARRIVE, PRISMA**) have been followed or provided.	Not Applicable	
For **tumor marker prognostic studies,** we recommend that you follow the **REMARK** reporting guidelines (see link list at top right). See author guidelines, under 'Reporting Guidelines'. Please confirm you have followed these guidelines.	Not Applicable	
For **phase II and III randomized controlled trials**, please refer to the **CONSORT** flow diagram (see link list at top right) and submit the CONSORT checklist (see link list at top right) with your submission. See author guidelines, under 'Reporting Guidelines'. Please confirm you have submitted this list.	Not Applicable	

Data Availability

Data availability	Information included in the manuscript?	In which section is the information available? (Reagents and Tools Table, Materials and Methods, Figures, Data Availability Section)
Have **primary datasets** been deposited according to the journal's guidelines (see 'Data Deposition' section) and the respective accession numbers provided in the Data Availability Section?	Not Applicable	
Were **human clinical and genomic datasets** deposited in a public access-controlled repository in accordance to ethical obligations to the patients and to the applicable consent agreement?	Not Applicable	
Are **computational models** that are central and integral to a study available without restrictions in a machine-readable form? Were the relevant accession numbers or links provided?	Yes	Material and Methods, Data Availability Statement and Code Availability Statement
If publicly available data were reused, provide the respective **data citations in the reference list**.	Yes	Data Avaialbility Section
